# Supplementary material for: How, in what contexts, and why do quality dashboards lead to improvements in care quality in acute hospitals? Protocol for a realist feasibility evaluation
Source: BMJ Open. 2020 Feb 25;10(2):e033208. doi: 10.1136/bmjopen-2019-033208 (PMC7044920; doi:10.1136/bmjopen-2019-033208)
Supplement: Supplementary data [file bmjopen-2019-033208supp002.pdf]

How, in what contexts, and why do quality dashboards lead to improvements in care quality in acute hospitals? Protocol for a realist feasibility evaluation (Randell et al.)

Additional file 2

#	Context	+	Mechanism: Resource	Mechanism: Response	=	Outcome
**1.**	Teams previously constrained in their ability to use NCA data for monitoring service performance because data not considered to be timely, accurate, and/or complete	**+**	QualDash offers easy access to key metrics	Teams are able to see whether the data displayed are timely, accurate, and/or complete and, where they are not, adjust their data collection processes in order to benefit from QualDash	**=**	Improvement in data quality in terms of timeliness, accuracy, and completeness – as data quality improves, use of QualDash increases
				Teams use QualDash to embed NCA data within their monitoring processes e.g. in clinical governance meetings where data is presented visually via screens.	**=**	Increased routine use of NCA data in performance monitoring, providing opportunities for its use in quality improvement
**2.**	Teams previously using NCA data to monitor service performance routinely by extracting raw data and producing reports for review in meetings and by individuals	**+**	QualDash visualises key metrics in ways that clearly show whether service performance is within an expected range and provides functions to interrogate that data	Teams use QualDash to facilitate their existing processes for monitoring service performance using NCA data	**=**	Reduced time spent in accessing, and preparing visualisations of, NCA data

Randell R, *et al. BMJ Open* 2020; 10:e033208. doi: 10.1136/bmjopen-2019-033208

#	Context	+	Mechanism		=	Outcome
			Resource	Response		
**3.**	Teams who want to use NCA data but were previously constrained by data quality and existing systems did not provide functions to easily access and interact with the data	**+**	QualDash provides functions that enable users to interact with NCA data and explore relationships between variables	Teams will use these functions to interrogate anomalies in the data, which will help them to understand what has impacted performance, thereby enabling them to identify appropriate strategies for improving performance	**=**	Introduction of QI initiatives in relation to metrics that teams consider important and where performance is not in line with expectations<br><br>Over time, improvement in metrics that QI initiatives target
**4.**	Performance in key metrics, such as the Best Practice Tariff, is in line with expectations<br><br>Relevant audit/IT support staff have time and willingness to support use of QualDash	**+**	QualDash offers teams the ability to quickly and easily add new QualCards (within NCA parameters)	Teams add new QualCards to be able to monitor and interrogate metrics they have chosen as important	**=**	Introduction of QI initiatives in relation to metrics shown on new QualCards when performance is not in line with expectations<br><br>Over time, improvement in metrics that QI initiatives target

#	Context	+	Mechanism		=	Outcome
			Resource	Response		
**5.**	Teams who previously did not, or were not able to, monitor key metrics routinely<br><br>Performance is not in line with expectations in key metrics<br><br>Teams are resourced to make practice changes	**+**	QualDash provides quick and easy access to key metrics	Teams will become aware of discrepancies between performance and targets in key metrics, which they will take action to address	**=**	Introduction of QI initiatives in relation to key metrics<br><br>Over time, improvement in those metrics
**6.**	Teams are asked to produce reports and recommendations for managers and other groups about service performance, e.g. at the time of publication of NCA annual report	**+**	QualDash offers easy access to NCA data and visualisations that can be exported into reports	Teams will use QualDash to produce performance reports requested by other groups	**=**	Reduced time spent in report preparation<br><br>Increased use of NCA data at divisional and corporate levels via outputs produced by QualDash<br><br>Over time, use of QualDash at divisional and/or corporate levels, due to increased awareness of NCA data

#	Context	+	Mechanism		=	Outcome
			**Resource**	**Response**		
**7.**	Teams receive data requests from service managers	+	QualDash can be easily accessed via the web by multiple users	Service managers will use QualDash to access the information they need quickly and easily	=	Streamlines the use of NCA data for clinical managers<br><br>Reduced time spent by audit support staff/clinical team in producing data reports for managers
**8.**	Teams need to evidence their performance to managers and other groups in order to support a case for practice change e.g. in business meetings with managers or in the NCA annual report summary	+	QualDash visualises performance metrics, which can also be exported into reports and presentations	Teams will use these functions to evidence service performance, in order to convince other Trust groups that change is needed	=	Other Trust groups, who are able to offer additional resource to teams, are convinced of the need for change based on the evidence provided. However, this is likely to be where those outputs are clearly associated with Trust priorities, e.g. relating to Trust reputation or avoiding penalties/gaining incentives.

Randell R, *et al. BMJ Open* 2020; 10:e033208. doi: 10.1136/bmjopen-2019-033208
