# Supplementary material for: How, in what contexts, and why do quality dashboards lead to improvements in care quality in acute hospitals? Protocol for a realist feasibility evaluation
Source: BMJ Open. 2020 Feb 25;10(2):e033208. doi: 10.1136/bmjopen-2019-033208 (PMC7044920; doi:10.1136/bmjopen-2019-033208)
Supplement: Supplementary data [file bmjopen-2019-033208supp003.pdf]

How, in what contexts, and why do quality dashboards lead to improvements in care quality in acute hospitals? Protocol for a realist feasibility evaluation (Randell et al.)
Additional file 3

The average CMOC for patients on a ward will be averaged for each month, so that there are 36 observations clustered within each of 15 hospitals. Taking the intra-class correlation to be 0.15, this yields a design effect of 6.25. Hence the effective number of observations is 15*36/6.25=86.4. Using Cohen's approach to sample size calculation means an effect size of 0.17 can be estimated with 80% given that there are six parameters in the model (including the coefficient for QualDash). Converting this to the percentage of variation that can be explained by the model, this yields 20.5%. Translating this back to CMOC, currently 49.6% of patients are discharged from hospital without missing any of the nine opportunities for care, and we would be powered at the 80% level to detect an improvement from an average of 8.33 opportunities achieved to 8.46. Thus our study has good power to detect small but meaningful clinical improvements. For PICANet, 10% of the admitted population receive non-invasive ventilation first [1]. On average there are 5.25 ventilation cases per month per hospital. With a further design effect from patients clustered within hospitals, based on the reported intraclass correlation coefficient of 0.065 giving a design effect of 1.276, the actual anticipated number of patients is 1701 giving an effective number of 213: 71 exposed to QualDash and 142 controls. This yields 80% power to detect a change from 32% to 53%.

References

1. Morris JV, Ramnarayan P, Parslow RC, Fleming SJ. Outcomes for Children Receiving Noninvasive Ventilation as the First-Line Mode of Mechanical Ventilation at Intensive Care Admission: A Propensity Score-Matched Cohort Study. Crit. Care Med. 2017.
