# Supplementary material for: How, in what contexts, and why do quality dashboards lead to improvements in care quality in acute hospitals? Protocol for a realist feasibility evaluation
Source: BMJ Open. 2020 Feb 25;10(2):e033208. doi: 10.1136/bmjopen-2019-033208 (PMC7044920; doi:10.1136/bmjopen-2019-033208)
Supplement: Author's manuscript [file bmjopen-2019-033208.draft_revisions.pdf]

BMJ Open is committed to open peer review. As part of this commitment we make the peer review history of every article we publish publicly available.

When an article is published we post the peer reviewers' comments and the authors' responses online. We also post the versions of the paper that were used during peer review. These are the versions that the peer review comments apply to.

The versions of the paper that follow are the versions that were submitted during the peer review process. They are not the versions of record or the final published versions. They should not be cited or distributed as the published version of this manuscript.

BMJ Open is an open access journal and the full, final, typeset and author-corrected version of record of the manuscript is available on our site with no access controls, subscription charges or pay-per-view fees (http://bmjopen.bmj.com).

If you have any questions on BMJ Open's open peer review process please email info.bmjopen@bmj.com

BMJ Open

How, in what contexts, and why do quality dashboards lead to improvements in care quality? Protocol for a realist feasibility evaluation

Journal:	*BMJ Open*
Manuscript ID	bmjopen-2019-033208
Article Type:	Protocol
Date Submitted by the Author:	25-Jul-2019
Complete List of Authors:	Randell, Rebecca; University of Leeds, School of Healthcare<br>Alvarado, Natasha; University of Leeds, School of Healthcare<br>McVey, Lynn; University of Leeds, School of Healthcare<br>Greenhalgh, Joanne; University of Leeds, Sociology and Social Policy<br>West, Robert; University of Leeds, Leeds Institute of Health Sciences<br>Farrin, Amanda; University of Leeds, Clinical Trials Research Unit<br>Gale, Chris; University of Leeds<br>Parslow, Roger; University of Leeds, Leeds Institute if Genetics, Health and Therapeutics<br>Keen, Justin; University of Leeds, Leeds Institute of Health Sciences<br>Elshehaly, Mai; University of Leeds, School of Computing<br>Ruddle, Roy; University of Leeds, School of Computing<br>Lake, Julia; Leeds Teaching Hospitals NHS Trust<br>Mamas, Mamas; Royal Stoke University Hospital<br>Feltbower, Richard; University of Leeds,<br>Dowding, Dawn; University of Manchester
Keywords:	AUDIT, Health informatics < BIOTECHNOLOGY & BIOINFORMATICS, Clinical audit < HEALTH SERVICES ADMINISTRATION & MANAGEMENT, Quality in health care < HEALTH SERVICES ADMINISTRATION & MANAGEMENT, QUALITATIVE RESEARCH

SCHOLARONE™
Manuscripts

BMJ

I, the Submitting Author has the right to grant and does grant on behalf of all authors of the Work (as defined in the below author licence), an exclusive licence and/or a non-exclusive licence for contributions from authors who are: i) UK Crown employees; ii) where BMJ has agreed a CC-BY licence shall apply, and/or iii) in accordance with the terms applicable for US Federal Government officers or employees acting as part of their official duties; on a worldwide, perpetual, irrevocable, royalty-free basis to BMJ Publishing Group Ltd ("BMJ") its licensees and where the relevant Journal is co-owned by BMJ to the co-owners of the Journal, to publish the Work in this journal and any other BMJ products and to exploit all rights, as set out in our licence.

The Submitting Author accepts and understands that any supply made under these terms is made by BMJ to the Submitting Author unless you are acting as an employee on behalf of your employer or a postgraduate student of an affiliated institution which is paying any applicable article publishing charge ("APC") for Open Access articles. Where the Submitting Author wishes to make the Work available on an Open Access basis (and intends to pay the relevant APC), the terms of reuse of such Open Access shall be governed by a Creative Commons licence – details of these licences and which Creative Commons licence will apply to this Work are set out in our licence referred to above.

Other than as permitted in any relevant BMJ Author's Self Archiving Policies, I confirm this Work has not been accepted for publication elsewhere, is not being considered for publication elsewhere and does not duplicate material already published. I confirm all authors consent to publication of this Work and authorise the granting of this licence.

For peer review only

Title: How, in what contexts, and why do quality dashboards lead to improvements in care quality? Protocol for a realist feasibility evaluation

Authors

Corresponding author: Rebecca Randell, School of Healthcare, University of Leeds, Leeds, UK, r.randell@leeds.ac.uk

Natasha Alvarado, of Healthcare, University of Leeds, Leeds, UK, n.alvarado@leeds.ac.uk

Lynn McVey, School of Healthcare, University of Leeds, Leeds, UK, l.mcvey@leeds.ac.uk

Joanne Greenhalgh, School of Sociology and Social Policy, University of Leeds, Leeds, UK, j.greenhalgh@leeds.ac.uk

Robert West, Leeds Institute of Health Sciences, University of Leeds, Leeds, UK, r.m.west@leeds.ac.uk

Amanda Farrin, Leeds Institute of Clinical Trials Research, University of Leeds, Leeds, UK, a.j.farrin@leeds.ac.uk

Chris P. Gale, School of Medicine, University of Leeds, Leeds, UK, c.p.gale@leeds.ac.uk

Roger Parslow, School of Medicine, University of Leeds, Leeds, UK, r.c.parslow@leeds.ac.uk

Justin Keen, Leeds Institute of Health Sciences, University of Leeds, Leeds, UK, j.keen@leeds.ac.uk

Mai Elshehaly, School of Computing, University of Leeds, Leeds, UK, m.h.elshehaly@leeds.ac.uk

Roy A. Ruddle, School of Computing, University of Leeds, Leeds, UK, r.a.ruddle@leeds.ac.uk

Julia Lake, Leeds Teaching Hospitals NHS Trust, Leeds, UK, julia.lake3@nhs.net

Mamas A. Mamas, Royal Stoke Hospital, Stoke on Trent, UK, mamasmamas1@yahoo.co.uk

Richard Feltbower, School of Medicine, University of Leeds, Leeds, UK, r.g.feltbower@leeds.ac.uk

Dawn Dowding, School of Health Sciences, University of Manchester, Manchester, UK, dawn.dowding@lmanchester.ac.uk

For peer review only

I, the Submitting Author has the right to grant and does grant on behalf of all authors of the Work (as defined in the below author licence), an exclusive licence and/or a non-exclusive licence for contributions from authors who are: i) UK Crown employees; ii) where BMJ has agreed a CC-BY licence shall apply, and/or iii) in accordance with the terms applicable for US Federal Government officers or employees acting as part of their official duties; on a worldwide, perpetual, irrevocable, royalty-free basis to BMJ Publishing Group Ltd ("BMJ") its licensees and where the relevant Journal is co-owned by BMJ to the co-owners of the Journal, to publish the Work in BMJ Open and any other BMJ products and to exploit all rights, as set out in our licence.

The Submitting Author accepts and understands that any supply made under these terms is made by BMJ to the Submitting Author unless you are acting as an employee on behalf of your employer or a postgraduate student of an affiliated institution which is paying any applicable article publishing charge ("APC") for Open Access articles. Where the Submitting Author wishes to make the Work available on an Open Access basis (and intends to pay the relevant APC), the terms of reuse of such Open Access shall be governed by a Creative Commons licence – details of these licences and which Creative Commons licence will apply to this Work are set out in our licence referred to above.

For peer review only

ABSTRACT

Introduction: National audits are used to monitor care quality and safety and are anticipated to reduce unexplained variations in quality by stimulating quality improvement. However, variation within and between providers in the extent to which they engage with national audits mean that the potential for national audit data to inform quality improvement is not being realised. This study aims to undertake a feasibility evaluation of QualDash, a quality dashboard designed to support clinical teams and managers to explore data from two national audits, the Myocardial Ischaemia National Audit Project (MINAP) and the Paediatric Intensive Care Audit Network (PICANet).

Methods and analysis: Realist evaluation, which involves building, testing, and refining theories of how an intervention is supposed to work, provides an overall framework. Realist hypotheses that describe how, in what contexts, and why QualDash is expected to provide benefit will be tested across five hospitals. A controlled interrupted time series analysis will investigate impacts of QualDash using key MINAP and PICANet measures. Ethnographic observations and interviews over 12 months will provide insight into contexts and mechanisms that lead to those impacts. Feasibility outcomes include the extent to which MINAP and PICANet data are used, data completeness in the audits, and the extent to which participants perceive QualDash to be useful and express the intention to continue using it after the study period.

Ethics and dissemination: The study has been approved by University of Leeds School of Healthcare Research Ethics Committee. Study results will provide an initial understanding of how, in what contexts, and why quality dashboards may lead to improvements in care quality. These will be disseminated to academic audiences, study participants, hospital IT departments, and national audits. If results show a trial of QualDash is feasible, we will disseminate the QualDash software through a stepped wedge cluster randomised trial.

Trial registration: ISRCTN18289782

Keywords: Dashboard, audit and feedback, quality improvement, realist evaluation

Word count: 3,984

For peer review only

For peer review only - http://bmjopen.bmj.com/site/about/guidelines.xhtml

ARTICLE SUMMARY

Strengths and limitations of this study

- This study combines a controlled interrupted time series study with a qualitative multi-site case study in order to provide an understanding of not only whether use of a quality dashboard leads to quality improvement but also how, in what contexts, and why.
- In addition to assessing the feasibility of a trial, the study will determine the components of QualDash to be preserved in a definitive trial, appropriate outcome measures, and the contexts in which a definitive trial should be undertaken.
- The study will contribute to understanding of how realist methods can contribute to feasibility studies and the design of trials.

INTRODUCTION

National clinical audits (NCAs), which provide comparative data on the performance of healthcare providers, are one means by which health systems around the world monitor care quality and safety. In England, a programme of over 60 NCAs is managed by the Healthcare Quality Improvement Partnership (HQIP) and all healthcare providers that contribute to delivery of the National Health Service (NHS) are required to participate. Such audits are anticipated to reduce unexplained variations in healthcare quality by stimulating quality improvement (QI) [1 2]. While there is evidence of positive impacts of NCAs [3-5], variation within and between providers in the extent to which they engage with NCAs mean that the potential for NCA data to inform QI is not being realised [6 7].

Quality dashboards are a form of audit and feedback (A&F) that provide visualisations of audit data with the aim of informing QI efforts [8]. Healthcare providers are increasingly using quality dashboards. For example, use of quality dashboards has been reported in Canada [9], the UK [10], and the Netherlands [11]. While quality dashboards have been shown to have positive effects on some performance indicators [9], empirical evidence regarding their impact remains limited [12].

For peer review only - http://bmjopen.bmj.com/site/about/guidelines.xhtml

QualDash

QualDash is an interactive web-based quality dashboard designed to support clinical teams and managers to explore data from two national audits, the Myocardial Ischaemia National Audit Project (MINAP) and the Paediatric Intensive Care Audit Network (PICANet), for the purpose of QI (Fig. 1). Information used to inform the design of QualDash was collected using a combination of methods, including: interviews, with 50 clinicians and managers across five NHS Trusts (providers) and four healthcare commissioners, that explored what supports and constrains their use of NCA data for QI; observations of meetings at different levels of the Trusts where audit data are discussed; a workshop with suppliers of NCAs; and two co-design workshops with clinicians and managers from one Trust.

Fig. 1: Prototype of main dashboard view for the Paediatric Intensive Care Audit Network (using simulated data).

[Figure 1 should go approximately here]

The interviews revealed that use of NCA data is largely at the clinical team level, with more limited use of NCA data at divisional and corporate (Board and sub-committees that report to the Board, such as Quality and Safety Committees) levels. At all levels, a key constraint in use of NCA data for QI is lack of access to timely data. QualDash seeks to overcome this constraint, providing users with a means to visualise their own data which they upload to the NCAs, rather than having to wait for data to be returned from the NCAs. There is also variation between Trusts in the extent to which NCA data are currently used, often related to resources; Trusts that make greater use of NCA data tend to have local databases from which they can generate visualisations of the data (e.g. bar charts) and audit support staff who have the time and skills to be able to generate such visualisations. Therefore, QualDash provides visualisations of key metrics, each metric being represented within a 'QualCard' (Fig. 2), enabling Trusts to use NCA data for QI, regardless of existing resources. Sites are also able to create additional QualCards, to reflect local priorities. However, the benefits perceived from using QualDash may vary between sites, with under-resourced sites that previously made little use of NCA data for QI perceiving greater impact than those sites that already have the means to use NCA data for this purpose. There are also constraints on use of NCA data for QI that it may be difficult for QualDash to address. For example, in some Trusts, clinical team members perceive that the relevant managers will not agree to

For peer review only - http://bmjopen.bmj.com/site/about/guidelines.xhtml

provide the resources necessary for QI initiatives, which reduces motivation to engage with NCA data and may affect the extent to which QualDash is used. However, QualDash provides means for visualisations to be downloaded and incorporated into presentations and reports, which may support clinical teams in making a stronger case for QI initiatives.

Fig. 2: Prototype of main dashboard view for the Paediatric Intensive Care Audit Network with the Mortality QualCard expanded (using simulated data).

[Figure 2 should go approximately here]

In this paper, we describe the methods for a realist feasibility evaluation of QualDash, informed by our understanding of how, in what contexts, and why NCA data are used and our expectations of how, in what contexts, and why QualDash will be used. The objectives of the study are:

1. To understand how, in what contexts, and why use of QualDash leads to QI; and
2. To assess the feasibility of conducting a randomised controlled trial (RCT) of QualDash.

As no checklists exist for the reporting of realist evaluation protocols, in presenting this protocol we draw on the RAMESES II reporting standards for realist evaluations [13] (Additional file 1).

METHODS AND ANALYSIS

Study design

Use of theory is needed for design and evaluation of A&F interventions [14-16], and QI initiatives more generally [17-19]. This project draws on Realist Evaluation (RE), which involves building, testing, and refining the underlying assumptions or theories of how an intervention is supposed to work [20]. These theories are expressed in the form of Context Mechanism Outcome (CMO) configurations, where C+M=O, reflecting the realist understanding that it is recipients' reasoning about and responses to the resources that the intervention provides (the intervention mechanisms) that determine the impact of the intervention, and such responses are highly influenced by context [21]. Consequently, RE seeks to answer not only the question of 'what works?' but 'what works for whom, in what circumstances, and why?' [22]. It is concerned with both intended and unintended outcomes. RE is recommended for

For peer review only - http://bmjopen.bmj.com/site/about/guidelines.xhtml

studying QI [23] and has been used successfully for studying the implementation and impact of large-scale QI programmes [24].

There is increasing interest in use of realist methods in feasibility evaluations [25-27]. By understanding the relationship between contexts, mechanisms, and outcomes, we aim to identify those components of QualDash associated with mechanisms that produce the desired outcomes in order for them to be preserved in a definitive trial, whereas other components may be adapted to suit the local context. By understanding both intended and unintended consequences, appropriate outcome measures can also be determined. Additionally, findings regarding contexts can be used to inform the decision about contexts in which the definitive trial should be undertaken, in terms of level of the organisation (clinical team, division, and/or corporate) and clinical area. This understanding will consequently inform which NCAs will be included in the trial.

We have drawn on a range of sources to develop CMO configurations which describe how, in what contexts, and why use of QualDash is anticipated to lead to QI (see Additional file 2). Data generated from the interviews, observations, and workshops described above have been essential to this, as have discussions with the designers of QualDash (ME and RAR) who, drawing on their expertise in information visualisation, have their own literature-informed theories regarding why certain features of QualDash will provide benefit to users [28 29]. We have also drawn on substantive theories regarding how A&F lead to QI at the individual micro level (Contextual Feedback Intervention Theory [30] and the model of actionable feedback [31]), the organisational meso level (Van Helden and Tilemma's model of benchmarking [32]), and the macro level (institutional theory [33 34]). The CMO configurations focus on use of QualDash by clinical teams, as this is where NCA data are most actively used, but also suggest how outputs produced via QualDash may become integrated in division and corporate quality monitoring processes.

Data collection is designed to enable testing of the CMO configurations. Outcome data, in the form of key MINAP and PICANet measures, will be collected and analysed in a controlled interrupted time series (CITS) study, while a multi-site case study [35] will provide insight into the contexts and mechanisms that lead to those outcomes, as well as providing data on intermediate outcomes such as increased use of

For peer review only - http://bmjopen.bmj.com/site/about/guidelines.xhtml

NCA data. A&F interventions, and QI interventions more generally, require longitudinal evaluation to allow sufficient time for staff to implement changes and incorporate them into their practice [36-38]. Similarly, evaluation of health IT (HIT) should allow time for staff to integrate the technology into their practices and evolve those practices to take advantage of the functionality offered by the technology [39]. Therefore, data will be collected over a 12 month period, from August 2019.

Public and patient involvement

A Lay Advisory Group has been established, who have contributed to the design of QualDash by reviewing the topic guide for the interviews that were conducted, providing their perspective on the findings of the interview study, and participating in the usability evaluation of QualDash. For the realist feasibility evaluation, they have provided advice on aspects to pay attention to when undertaking observations. They will contribute to analysis of a sample of the qualitative data, to provide a patient perspective. They will advise on dissemination of findings to relevant interest groups and will contribute to the creation of outputs by reviewing them for comprehensibility.

Setting/context

QualDash will be evaluated in the five NHS acute Trusts in which the interview study that informed the design of QualDash was undertaken. Three Trusts are teaching hospitals that participate in both MINAP and PICANet and have been selected to ensure variation in key outcome measures (MINAP: 30-day mortality for patients hospitalised with ST-elevation myocardial infarction; PICANet: risk adjusted standardised mortality ratio). Two Trusts are District General Hospitals (DGHs) that participate in MINAP but do not have a PICU and so do not participate in PICANet. These have been selected to ensure variation in the same key MINAP measure.

Multi-site case study

In the multi-site case study, data will be collected through ethnographic observation and interviews. Ethnographic methods, such as non-participant observation, have been argued as essential for studying the implementation of QI interventions [19] and the introduction of HIT [40]. Ethnography is well suited to RE because it involves observing phenomena in context, supporting understanding of how context influences the response to an intervention [41]. We will follow the Biography of Artefacts

For peer review only

approach [42], which is concerned with capturing the way in which particular contexts and appropriations of a technology lead to different processes and generate different outcomes, a parallel to RE's concern with contexts, mechanisms, and outcomes [43]. It involves longitudinal 'strategic ethnography' [42], where data collection is guided by a provisional understanding of the moments and locales in which a technology and associated practices evolve [43].

Data collection

In the three teaching hospitals, we will undertake a minimum of 24 periods of observation per Trust, to be split across activities related to cardiology and the PICU, and in the two DGHs we will undertake a minimum of 12 periods of observation per Trust, to be spent observing activities related to cardiology. Each period of observation will be a minimum of four hours (total n=384 hours). While the researchers will return to each Trust monthly, to understand how use of QualDash changes over time, more time will be spent in the first few months following the introduction of QualDash, because this is when users are most likely to engage with and explore the affordances of QualDash and establish new practices around it, generating information with implications for system enhancement [43]. Observations will be scheduled to take place at different times of day and on different days of the week, to ensure that the account of what is observed is as complete and representative as possible [44].

At each case site, an initial phase of general observation will provide an opportunity for researchers to become familiar with the setting and for those in the setting to become familiar with the presence of the researchers. Following a previous study of dashboards [10], observations will be undertaken in clinical areas to understand clinical teams' working practices and capture 'corridor committees' where issues of quality and safety are discussed more informally [45]. In the PICUs, initial observations will take place on the PICU, for example with the researchers positioning themselves by the nurses' station, as well as observing handovers, safety huddles, and ward rounds. Because activities related to cardiology tend to be more dispersed across hospitals, researchers will first shadow clinical team members (consultant cardiologists and acute chest pain nurses) to determine where it is most appropriate to conduct subsequent observations. These initial observations will also provide the opportunity to record general details of the setting that may influence use of QualDash, such as staffing levels and availability of computers.

For peer review only - http://bmjopen.bmj.com/site/about/guidelines.xhtml

After this initial phase, observation will be guided by the CMO configurations under investigation. In addition to observing formal meetings where quality and safety are discussed, predominantly at ward level but also at divisional and corporate level, observation will involve shadowing staff members as they undertake particular activities: collection and entry of NCA data, to see if and how this changes over time; accessing and interrogating NCA data, whether using QualDash or some other means; preparation of reports and/or presentations using NCA data, again whether using QualDash or some other means. Where visualisations from QualDash are incorporated into presentations and written reports, we will follow the path of those documents, to identify staff members who may not use QualDash directly but are receiving QualDash outputs. Attention will be paid to how, in what contexts, and why QualDash and QualDash outputs are used or not, understood in the context of broader practices and use of other sources of information for monitoring care quality, and how this changes over time. We will also follow local QI initiatives, recording data on, for example, when and how the need for the QI initiative was identified, contextual factors that appear to support and constrain its introduction, how the impact of the QI initiative is monitored, and other contextual factors that appear to influence the metric that the QI initiative is targeting.

Researchers will record observations in fieldnotes. The scope of the notes will be kept wide on the basis that what previously seemed insignificant may come to take on new meaning in light of subsequent events [44]. In addition, the researchers will record incidents of observer effects (e.g. participants asking 'What are you writing?') to allow analysis of whether participants' awareness of the researchers' presence changed over time [46]. Fieldnotes will be written up in detail as soon after data collection as possible.

Brief interviews will be undertaken opportunistically during the course of conducting observations to clarify aspects of practice that are not immediately intelligible to an observer, with participant responses recorded in fieldnotes [47]. As data collection progresses, longer semi-structured interviews will be used to discuss revisions to our CMO configurations. These interviews will be undertaken using a particular approach from RE, referred to as the teacher-learner cycle, whereby the theories under investigation are made explicit to the interviewee so that the interviewee can use their experiences to refine the

For peer review only - http://bmjopen.bmj.com/site/about/guidelines.xhtml

researcher's understanding [48]. Additionally, being concerned with the reasoning of intervention recipients, mechanisms are often not observable [21] and so these longer interviews will also provide the opportunity to explore staff reasoning about QualDash. These longer interviews will be audio recorded and transcribed verbatim.

Logfiles are widely used to evaluate visualisation tools [49] and with QualDash will record information about a user (job title, etc.), data used (audit, year, variables displayed, etc.), overall time spent using QualDash, functionality used, and whether the user downloaded the QualDash visualisations. In addition to providing important data regarding extent of QualDash use, how QualDash is used and by whom (e.g. whether the most frequent users are nurses, consultants, or audit support staff), and how this changes over time, information from logfiles will be used to inform qualitative data collection (e.g. asking in interviews why participants use particular QualCards and not others).

At the end of the data collection period, we will ask participants to complete a questionnaire based on the Technology Acceptance Model, using well validated items that have been used in numerous evaluations of HIT [50], including dashboards [51]. This will provide participants' perceptions of the usefulness of QualDash and data on whether they intend to continue using QualDash after the study period.

Analysis

An iterative approach to data collection and analysis will be taken, to enable: ongoing testing and refinement of the CMO configurations; gathering of further data in light of such revisions; and refinement of QualDash in response to participants' feedback. Fieldnotes and interview transcripts will be entered into NVivo 11. Narrative analysis will be undertaken to develop a 'biography' of QualDash, which will describe use of QualDash and its outputs by a range of stakeholders at different levels (clinical team, divisional, and corporate) and the interconnections between them [10]. Narrative analysis is consistent with a realist approach due to its emphasis on preserving connections within the data, thereby helping to understand causality [52]. This analysis will be supplemented with analysis of the logfiles and questionnaire data. Findings will be compared with the CMO configurations, to determine whether they support, refute, or suggest a revision or addition to the CMO configurations.

For peer review only - http://bmjopen.bmj.com/site/about/guidelines.xhtml

Controlled interrupted time series study

CITS studies provide a robust method of assessing the effect of an intervention and have been used to assess the effectiveness of a variety of complex interventions [53]. Data will be collected across the five Trusts, with two control Trusts per intervention Trust. Control Trusts will be matched according to their size and outcomes pre-intervention.

Given the study intention to determine the feasibility of and inform the design of a trial, a range of measures will be considered. Initially, we selected two process measures, one for MINAP and one for PICANet. For MINAP, we selected the composite process measure Cumulative Missed Opportunities for Care (CMOC). This has nine components (pre-hospital ECG, acute use of aspirin, timely perfusion, referral for cardiac rehabilitation, and prescription at hospital discharge of what are considered to be the gold standard drugs – aspirin, thienopyridine inhibitor, ACE-inhibitor, HMG-CoA reductase inhibitor, and beta blockers) and is inversely associated with mortality [54]. As some of these components, such as pre-hospital ECG, are outside the direct control of the Trust, we will also explore the impact of QualDash on the individual measures that make up CMOC. On the basis of the measures that cardiology clinicians described in the interview study as being important for measuring care quality, we will also look at the percentage of patients who receive an angiogram within 72 hours from first admission to hospital, which is part of the Best Practice Tariff financial incentive scheme, and, for those hospitals that provide percutaneous coronary intervention (PCI), the proportion of patients who have a door-to-balloon time (the time from arrival at the hospital to PCI) of less than 60 minutes. Our CMO configurations (Additional file 2) suggest that improvement will be seen in measures if: clinical teams perceive them as being important indicators of care and/or they relate to financial incentives; performance is not in line with expectations; they perceive the measure as being within their control; and the team is resourced to introduce QI initiatives in relation to these measures.

For PICANet, we initially selected use of non-invasive ventilation first for patients requiring ventilation, which has been shown to be associated with reduced mortality [55]. However, this was not raised as an area of concern in our interviews with PICU clinicians. On the basis of this and two additional considerations – it would require loading additional data into QualDash which would reduce the

For peer review only - http://bmjopen.bmj.com/site/about/guidelines.xhtml

performance of QualDash in terms of speed and it requires computation of the data, while the focus of QualDash is on visualising the data – a QualCard has not been created for this metric. Therefore, we do not hypothesise that this measure will change, unless other sources of information, such as the PICANet annual report, draw a PICU team's attention to it. However, accidental extubation was identified in our interviews with PICU clinicians as being an important indicator of care quality; QualDash includes a QualCard for accidental extubation, which displays the number of patients receiving invasive ventilation, and we will include this as a measure. Unplanned readmission within 48 hours was also identified in our interviews as being an important indicator of care quality, so a QualCard for this has been created and we will include this as a measure. On the basis of our CMO configurations (Additional file 2), we would expect to see an improvement in these measures in sites where performance is not in line with expectations, if the team is resourced to introduce QI initiatives in relation to these measures.

Sample size considerations

A CITS study requires data for a minimum of three time points pre-intervention and three time points post-intervention and must also allow for any seasonal effect on the outcomes [56]. Monthly data will be obtained for 24 months pre-intervention and 12 months post-intervention. Consequently, for each intervention Trust, there will be 72 data points prior to introduction (24 for the intervention Trust and 48 for the control Trusts) and 36 data points post intervention (12 for the intervention Trust and 24 for the control Trusts).

Analysis

Monthly MINAP and PICANet data will be extracted to spreadsheets for analysis with R software [57]. For both NCAs, each outcome will be regressed upon time and the intervention. The time component will include a seasonal effect (quarterly effect) and will allow for a (linear) time trend. To account for clustering of monthly observations within hospitals a random intercept will be fitted, although a fixed effect for hospital as a sensitivity analysis will be explored. Although the intervention is abrupt, its impact may well be 'phased in' over a few months, perhaps three. The timing of the bedding in of the intervention will be reported from the multi-site case study. Then a partial effect can be considered for this period with the interaction effect stepping up in a linear fashion.

For peer review only - http://bmjopen.bmj.com/site/about/guidelines.xhtml

The results of the CITS analysis will be incorporated into the biography of QualDash, the analysis of the data from the multi-site case study describing how contextual factors shape the evolution of practices around QualDash and how this leads to the resulting outcome pattern.

Assessment of trial feasibility

Criteria for progression to an RCT are: (i) the number of people who engage with either MINAP or PICANet data (via QualDash or some other means) is the same or higher than the number of people who engaged with either MINAP or PICANet data prior to QualDash's introduction; (ii) data completeness in the national audit improves or remains the same; (iii) 50% or more of participants in the questionnaire survey perceive QualDash to be useful and express the intention to continue using it after the study period. Criteria (i) and (ii) are concerned with ensuring that the intervention does not have unintended negative consequences which would affect the success of the intervention. Criterion (ii) is also concerned with the feasibility of outcome assessment. Criterion (iii) is concerned with acceptability and uptake of the intervention, and therefore has implications for recruitment to a trial, as well as being concerned with participants' perceptions of the impact of QualDash on care. While not formally assessed as part of the progression criteria, the impact of QualDash on processes of care as identified in the CITS will also be considered in determining whether a future cluster randomised controlled trial is justified. A traffic light system will be used to determine if a trial is feasible (green), feasible with modifications to QualDash (amber), or not feasible (red) [58 59].

ETHICS AND DISSEMINATION

Ethics approval has been received from the University of Leeds School of Healthcare Research Ethics Committee (Approval no.HREC16-044). Written consent will be obtained from participants for interviews and for meeting observations.

Study results will provide an initial understanding of how, in what contexts, and why quality dashboards may lead to improvements in care quality. We will disseminate these results to academic audiences, study participants, hospital IT departments, and National Clinical Audits. If the results show a trial of QualDash is feasible, we will design a stepped wedge cluster randomised trial, which will, in addition to

For peer review only - http://bmjopen.bmj.com/site/about/guidelines.xhtml

providing further understanding of the impact of quality dashboards on care quality, result in wider dissemination of the QualDash software.

Acknowledgements

This research is funded by the National Institute for Health Research (NIHR) Health Services and Delivery Research (HS&DR) Programme (project number 16/04/06). The views and opinions expressed are those of the presenter and do not necessarily reflect those of the HS&DR programme, NIHR, NHS or the Department of Health.

Data statement

The qualitative data gathered during the course of this evaluation will be kept until June 2030 and can be accessed by other researchers during this time, subject to the necessary ethical approvals being obtained. Requests for access to this data should be addressed to the corresponding author.

Authors' contributions

RR is Principal Investigator for the study. She conceived, designed, and secured funding for the study in collaboration with JG, RW, AF, CPG, RP, JK, RAR, JL, MM, and DD. NA and LM led the qualitative data collection and analysis that informed the design of QualDash and the design of evaluation. ME developed the QualDash software and contributed to the design of the evaluation. RP and RF provided data for the testing of QualDash and provided significant feedback on its design. All authors provided input into various aspects of the evaluation design and revised drafts of the protocol. RR led the writing of this protocol manuscript. All authors read and approved the final manuscript.

Funding statement

This research is funded by the National Institute for Health Research (NIHR) Health Services and Delivery Research (HS&DR) Programme (project number 16/04/06).

For peer review only

Competing interests statement

Chris Gale is a member of the MINAP Academic and Steering Groups. Richard Feltbower is Principal Investigator for PICANet and Roger Parslow was previously Principal Investigator for PICANet. The authors have no other competing interests to declare.

Additional files

Additional file 1: Checklist of RAMESES II reporting standards for realist evaluations (PDF)

Additional file 2: Context Mechanism Outcome configurations to be tested in the realist feasibility evaluation (PDF)

For peer review only

REFERENCES

1. Phekoo KJ, Clements J, Bell D. National Clinical Audit Quality Assessment - Overview of the self-assessment survey: "audit of audits". London: Healthcare Quality Improvement Partnership, 2014.
2. Miller T, Leatherman S. The National Quality Forum: a'me-too'or a breakthrough in quality measurement and reporting? Health Aff. (Millwood) 1999;**18**(6):233-37.
3. Birkhead JS, Walker L, Pearson M, Weston C, Cunningham AD, Rickards AF. Improving care for patients with acute coronary syndromes: initial results from the National Audit of Myocardial Infarction Project (MINAP). Heart 2004;**90**(9):1004-09.
4. Bridgewater B, Grayson AD, Brooks N, et al. Has the publication of cardiac surgery outcome data been associated with changes in practice in northwest England: an analysis of 25 730 patients undergoing CABG surgery under 30 surgeons over eight years. Heart 2007;**93**(6):744-48.
5. Neuburger J, Currie C, Wakeman R, et al. The Impact of a National Clinician-led Audit Initiative on Care and Mortality after Hip Fracture in England: An External Evaluation using Time Trends in Non-audit Data. Med. Care 2015;**53**(8):686-91.
6. Allwood D. Engaging Clinicians in Quality Improvement through National Clinical Audit. London: Healthcare Quality Improvement Partnership, 2014.
7. Taylor A, Neuburger J, Walker K, Cromwell D, Groene O. How is feedback from national clinical audits used? Views from English National Health Service trust audit leads. J. Health Serv. Res. Policy 2016;**21**(2):91-100.
8. Pauwels K, Ambler T, Clark BH, et al. Dashboards as a Service Why, What, How, and What Research Is Needed? Journal of Service Research 2009;**12**(2):175-89.
9. Weiss D, Dunn SI, Sprague AE, et al. Effect of a population-level performance dashboard intervention on maternal-newborn outcomes: an interrupted time series study. BMJ Quality & Safety 2018;**27**(6):425-36.
10. Keen J, Nicklin E, Long A, et al. Quality and safety between ward and board: a biography of artefacts study. Health Services and Delivery Research 2018;**6**(22).
11. Weggelaar-Jansen AMJWM, Broekharst DSE, de Bruijne M. Developing a hospital-wide quality and safety dashboard: a qualitative research study. BMJ Quality & Safety 2018.
12. Dowding D, Randell R, Gardner P, et al. Dashboards for improving patient care: Review of the literature. International Journal of Medical Informatics 2015;**84**(2):87-100.
13. Wong G, Westhorp G, Manzano A, Greenhalgh J, Jagosh J, Greenhalgh T. RAMESES II reporting standards for realist evaluations. BMC Med. 2016;**14**(1):96.

For peer review only - http://bmjopen.bmj.com/site/about/guidelines.xhtml

14. Foy R, Eccles MP, Jamtvedt G, Young J, Grimshaw JM, Baker R. What do we know about how to do audit and feedback? Pitfalls in applying evidence from a systematic review. BMC Health Serv. Res. 2005;**5**:50.
15. Ivers NM, Grimshaw JM, Jamtvedt G, et al. Growing Literature, Stagnant Science? Systematic Review, Meta-Regression and Cumulative Analysis of Audit and Feedback Interventions in Health Care. J. Gen. Intern. Med. 2014;**29**(11):1534-41.
16. Ivers NM, Sales A, Colquhoun H, et al. No more 'business as usual' with audit and feedback interventions: towards an agenda for a reinvigorated intervention. Implementation Science 2014;**9**(1):1-8.
17. Grol RPTM, Bosch MC, Hulscher MEJL, Eccles MP, Wensing M. Planning and Studying Improvement in Patient Care: The Use of Theoretical Perspectives. Milbank Q. 2007;**85**(1):93-138.
18. Davidoff F, Dixon-Woods M, Leviton L, Michie S. Demystifying theory and its use in improvement. BMJ Quality & Safety 2015.
19. Dixon-Woods M, Bosk CL, Aveling EL, Goeschel CA, Pronovost PJ. Explaining Michigan: Developing an Ex Post Theory of a Quality Improvement Program. Milbank Q. 2011;**89**(2):167-205.
20. Pawson R, Tilley N. *Realistic Evaluation*. London: SAGE Publications, 1997.
21. Dalkin S, Greenhalgh J, Jones D, Cunningham B, Lhussier M. What's in a mechanism? Development of a key concept in realist evaluation. Implementation Science 2015;**10**(1):49.
22. Pawson R. *The science of evaluation: a realist manifesto*. London: SAGE, 2013.
23. Robert G, Fulop N. The role of context in successful improvement. Perspectives on context. A selection of essays considering the role of context in successful quality improvement. London: Health Foundation 2014;**31**.
24. Greenhalgh T, Humphrey C, Hughes J, Macfarlane F, Butler C, Pawson R. How Do You Modernize a Health Service? A Realist Evaluation of Whole-Scale Transformation in London. Milbank Q. 2009;**87**(2):391-416.
25. Fletcher A, Jamal F, Moore G, Evans RE, Murphy S, Bonell C. Realist complex intervention science: Applying realist principles across all phases of the Medical Research Council framework for developing and evaluating complex interventions. Evaluation 2016;**22**(3):286-303.
26. Brand SL, Quinn C, Pearson M, et al. Building programme theory to develop more adaptable and scalable complex interventions: Realist formative process evaluation prior to full trial. Evaluation 2019;**25**(2):149-70.
27. Randell R, Honey S, Hindmarsh J, et al. A realist process evaluation of robot-assisted surgery: integration into routine practice and impacts on communication, collaboration and decision-making. Health Services and Delivery Research 2017;**5**(20).
28. Andrienko N, Andrienko G. *Exploratory analysis of spatial and temporal data: a systematic approach*: Springer Science & Business Media, 2006.
29. Brehmer M, Munzner T. A multi-level typology of abstract visualization tasks. IEEE transactions on visualization and computer graphics 2013;**19**(12):2376-85.
30. Sapyta J, Riemer M, Bickman L. Feedback to clinicians: Theory, research, and practice. J. Clin. Psychol. 2005;**61**(2):145-53.
31. Hysong SJ, Best RG, Pugh JA. Audit and feedback and clinical practice guideline adherence: Making feedback actionable. Implementation Science 2006;**1**(1):1-10.
32. Van Helden GJ, Tillema S. In Search of a Benchmarking Theory for the Public Sector. Financial Accountability & Management 2005;**21**(3):337-61.
33. Scott RW. *Institutions and Organizations*. 2nd Edition ed. London: Sage, 2001.
34. Furusten S. *Institutional Theory and Organizational Change*. Cheltenham, UK: Edward Elgar, 2013.
35. Yin RK. *Case study research: design and methods*. 3rd ed. Thousand Oaks, California: SAGE, 2003.
36. Øvretveit J, Gustafson D. Evaluation of quality improvement programmes. Quality and Safety in Health Care 2002;**11**(3):270-75.

For peer review only - http://bmjopen.bmj.com/site/about/guidelines.xhtml

For peer review only

37. Ramsay AIG, Turner S, Cavell G, et al. Governing patient safety: lessons learned from a mixed methods evaluation of implementing a ward-level medication safety scorecard in two English NHS hospitals. BMJ Quality & Safety 2013;**23**:136-46.
38. Benn J, Burnett S, Parand A, Pinto A, Iskander S, Vincent C. Studying large-scale programmes to improve patient safety in whole care systems: Challenges for research. Soc. Sci. Med. 2009;**69**(12):1767-76.
39. Hyysalo S. *Health technology development and use: from practice-bound imagination to evolving impacts*. New York: Routledge, 2010.
40. Greenhalgh T, Swinglehurst D. Studying technology use as social practice: the untapped potential of ethnography. BMC Med. 2011;**9**(1):45.
41. Dainty KN, Golden BR, Hannam R, et al. A realist evaluation of value-based care delivery in home care: The influence of actors, autonomy and accountability. Soc. Sci. Med. 2018;**206**:100-09.
42. Pollock N, Williams R. *Software and organisations: The biography of the enterprise-wide system or how SAP conquered the world*. New York: Routledge, 2008.
43. Pollock N, Williams R. e-Infrastructures: How Do We Know and Understand Them? Strategic Ethnography and the Biography of Artefacts. Computer Supported Cooperative Work (CSCW) 2010;**19**(6):521-56.
44. Hammersley M, Atkinson P. *Ethnography: principles in practice*. London: Routledge, 1995.
45. Waring J. Adaptive regulation or governmentality: patient safety and the changing regulation of medicine. Sociol. Health Illn. 2007;**29**(2):163-79.
46. McDonald S. Studying actions in context: a qualitative shadowing method for organizational research. Qualitative Research 2005;**5**(4):455-73.
47. Goodwin D, Pope C, Mort M, Smith A. Access, boundaries and their effects: legitimate participation in anaesthesia. Sociol. Health Illn. 2005;**27**(6):855-71.
48. Pawson R. Theorizing the Interview. The British Journal of Sociology 1996;**47**(2):295-314.
49. Harrison DG, Efford ND, Fisher QJ, Ruddle RA. PETMiner—A Visual Analysis Tool for Petrophysical Properties of Core Sample Data. IEEE transactions on visualization and computer graphics 2017;**24**(5):1728-41.
50. Holden RJ, Karsh B-T. The Technology Acceptance Model: Its past and its future in health care. Journal of Biomedical Informatics 2010;**43**(1):159-72.
51. Lee K, Jung SY, Hwang H, et al. A novel concept for integrating and delivering health information using a comprehensive digital dashboard: An analysis of healthcare professionals' intention to adopt a new system and the trend of its real usage. Int. J. Med. Inform. 2017;**97**:98-108.
52. Maxwell JA. *A realist approach for qualitative research*. London: SAGE Publications, 2012.
53. Hu Y. Stepped wedge, natural experiments and interrupted time series analysis designs. In: Richards DA, Hallberg IR, eds. Complex interventions in health: An overview of research methods. Abingdon: Routledge, 2015:200-12.
54. Simms AD, Baxter PD, Cattle BA, et al. An assessment of composite measures of hospital performance and associated mortality for patients with acute myocardial infarction. Analysis of individual hospital performance and outcome for the National Institute for Cardiovascular Outcomes Research (NICOR). European Heart Journal: Acute Cardiovascular Care 2013;**2**(1):9-18.
55. Morris JV, Ramnarayan P, Parslow RC, Fleming SJ. Outcomes for Children Receiving Noninvasive Ventilation as the First-Line Mode of Mechanical Ventilation at Intensive Care Admission: A Propensity Score-Matched Cohort Study. Crit. Care Med. 2017.
56. Cochrane Effective Practice and Organisation of Care Group (EPOC). What study designs should be inlcuded in an EPOC review and what should they be called? Oslo: Norwegian Knowledge Centre for the Health Services, 2013.
57. R Core Team. R: A language and environment for statistical computing. Vienna, Austria: R Foundation for Statistical Computing, 2014.

For peer review only - http://bmjopen.bmj.com/site/about/guidelines.xhtml

58. Bugge C, Williams B, Hagen S, et al. A process for Decision-making after Pilot and feasibility Trials (ADePT): development following a feasibility study of a complex intervention for pelvic organ prolapse. Trials 2013;**14**(1):353.
59. Thabane L, Ma J, Chu R, et al. A tutorial on pilot studies: the what, why and how. BMC Med. Res. Methodol. 2010;**10**(1):1-10.

For peer review only

For peer review only - http://bmjopen.bmj.com/site/about/guidelines.xhtml

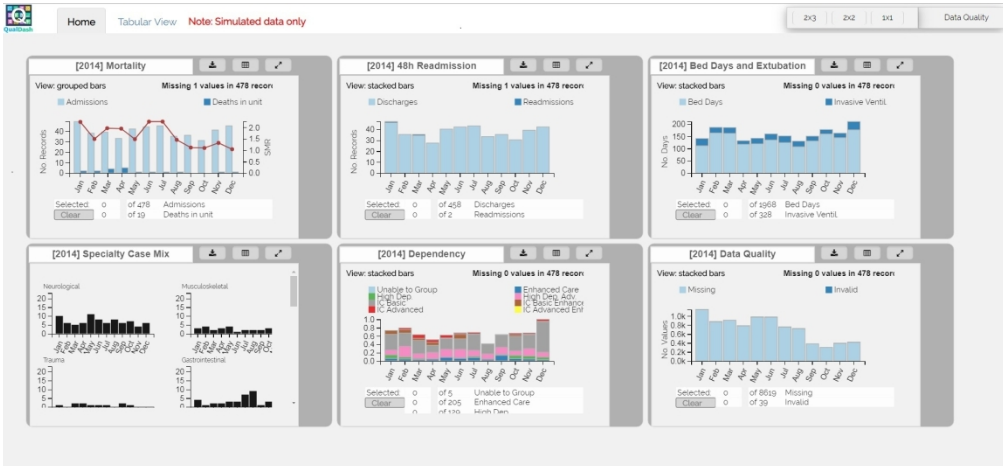

Fig. 1: Prototype of main dashboard view for the Paediatric Intensive Care Audit Network (using simulated data).

161x74mm (300 x 300 DPI)

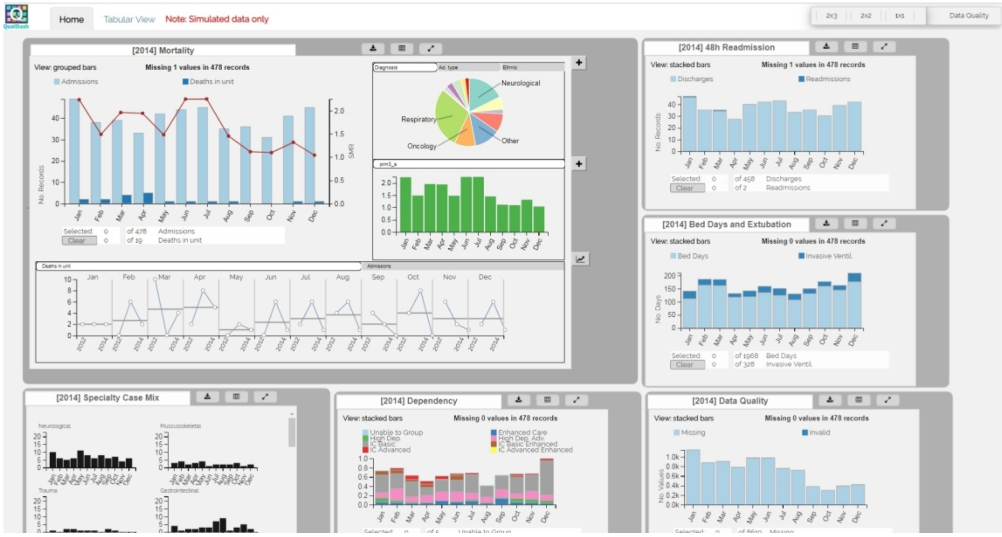

Fig. 2: Prototype of main dashboard view for the Paediatric Intensive Care Audit Network with the Mortality QualCard expanded (using simulated data).

161x85mm (300 x 300 DPI)

How, in what contexts, and why do quality dashboards lead to improvements in care quality? Protocol for a realist feasibility evaluation (Randell et al.)

Additional file 2

#	Context	+	Mechanism: Resource	Mechanism: Response	=	Outcome
**1.**	Teams previously constrained in their ability to use NCA data for monitoring service performance because data not considered to be timely, accurate, and/or complete	**+**	QualDash offers easy access to key metrics	Teams are able to see whether the data displayed are timely, accurate, and/or complete and, where they are not, adjust their data collection processes in order to benefit from QualDash	**=**	Improvement in data quality in terms of timeliness, accuracy, and completeness – as data quality improves, use of QualDash increases
				Teams use QualDash to embed NCA data within their monitoring processes e.g. in clinical governance meetings where data is presented visually via screens.	**=**	Increased routine use of NCA data in performance monitoring, providing opportunities for its use in quality improvement
**2.**	Teams previously using NCA data to monitor service performance routinely by extracting raw data and producing reports for review in meetings and by individuals	**+**	QualDash visualises key metrics in ways that clearly show whether service performance is within an expected range and provides functions to interrogate that data	Teams use QualDash to facilitate their existing processes for monitoring service performance using NCA data	**=**	Reduced time spent in accessing, and preparing visualisations of, NCA data

For peer review only

#	Context	+	Mechanism		=	Outcome
			Resource	Response		
**3.**	Teams who want to use NCA data but were previously constrained by data quality and existing systems did not provide functions to easily access and interact with the data	+	QualDash provides functions that enable users to interact with NCA data and explore relationships between variables	Teams will use these functions to interrogate anomalies in the data, which will help them to understand what has impacted performance, thereby enabling them to identify appropriate strategies for improving performance	=	Introduction of QI initiatives in relation to metrics that teams consider important and where performance is not in line with expectations<br><br>Over time, improvement in metrics that QI initiatives target
**4.**	Performance in key metrics, such as the Best Practice Tariff, is in line with expectations<br><br>Relevant audit/IT support staff have time and willingness to support use of QualDash	+	QualDash offers teams the ability to quickly and easily add new QualCards (within NCA parameters)	Teams add new QualCards to be able to monitor and interrogate metrics they have chosen as important	=	Introduction of QI initiatives in relation to metrics shown on new QualCards when performance is not in line with expectations<br><br>Over time, improvement in metrics that QI initiatives target

For peer review only

#	Context	+	Mechanism		=	Outcome
			Resource	Response		
**5.**	Teams who previously did not, or were not able to, monitor key metrics routinely<br><br>Performance is not in line with expectations in key metrics<br><br>Teams are resourced to make practice changes	+	QualDash provides quick and easy access to key metrics	Teams will become aware of discrepancies between performance and targets in key metrics, which they will take action to address	=	Introduction of QI initiatives in relation to key metrics<br><br>Over time, improvement in those metrics
**6.**	Teams are asked to produce reports and recommendations for managers and other groups about service performance, e.g. at the time of publication of NCA annual report	+	QualDash offers easy access to NCA data and visualisations that can be exported into reports	Teams will use QualDash to produce performance reports requested by other groups	=	Reduced time spent in report preparation<br><br>Increased use of NCA data at divisional and corporate levels via outputs produced by QualDash<br><br>Over time, use of QualDash at divisional and/or corporate levels, due to increased awareness of NCA data

For peer review only

For peer review only - http://bmjopen.bmj.com/site/about/guidelines.xhtml

#	Context	+	Mechanism		=	Outcome
			Resource	Response		
**7.**	Teams receive data requests from service managers	**+**	QualDash can be easily accessed via the web by multiple users	Service managers will use QualDash to access the information they need quickly and easily	**=**	Streamlines the use of NCA data for clinical managers<br><br>Reduced time spent by audit support staff/clinical team in producing data reports for managers
**8.**	Teams need to evidence their performance to managers and other groups in order to support a case for practice change e.g. in business meetings with managers or in the NCA annual report summary	**+**	QualDash visualises performance metrics, which can also be exported into reports and presentations	Teams will use these functions to evidence service performance, in order to convince other Trust groups that change is needed	**=**	Other Trust groups, who are able to offer additional resource to teams, are convinced of the need for change based on the evidence provided. However, this is likely to be where those outputs are clearly associated with Trust priorities, e.g. relating to Trust reputation or avoiding penalties/gaining incentives.

For peer review only - http://bmjopen.bmj.com/site/about/guidelines.xhtml

RAMESES II reporting standards for realist evaluations

How, in what contexts, and why do quality dashboards lead to improvements in care quality? Protocol for a realist feasibility evaluation			Reported in document Y/N/NA	Page(s) in document	Comment
1		In the title, identify the document as a realist evaluation	Y	1	
SUMMARY OR ABSTRACT					
2		Journal articles will usually require an abstract, while reports and other forms of publication will usually benefit from a short summary. The abstract or summary should include brief details on: the policy, programme or initiative under evaluation; programme setting; purpose of the evaluation; evaluation question(s) and/or objective(s); evaluation strategy; data collection, documentation and analysis methods; key findings and conclusions<br><br>Where journals require it and the nature of the study is appropriate, brief details of respondents to the	Y	2	

For peer review only

		evaluation and recruitment and sampling processes may also be included<br><br>Sufficient detail should be provided to identify that a realist approach was used and that realist programme theory was developed and/or refined			
INTRODUCTION					
3	Rationale for evaluation	Explain the purpose of the evaluation and the implications for its focus and design	Y	6	
4	Programme theory	Describe the initial programme theory (or theories) that underpin the programme, policy or initiative	Y	7-8 and Additional file 1	Placed in body of article, rather than Introduction, as more appropriate for protocol
5	Evaluation questions, objectives and focus	State the evaluation question(s) and specify the objectives for the evaluation. Describe whether and how the programme theory was used to define the scope and focus of the evaluation	Y	6	
6	Ethical approval	State whether the realist evaluation required and has gained ethical approval from the relevant	Y	15	Stated under declarations as required by journal

For peer review only

		authorities, providing details as appropriate. If ethical approval was deemed unnecessary, explain why			
METHODS					
7	Rationale for using realist evaluation	Explain why a realist evaluation approach was chosen and (if relevant) adapted	Y	7	
8	Environment surrounding the evaluation	Describe the environment in which the evaluation took place	Y	8	
9	Describe the programme policy, initiative or product evaluated	Provide relevant details on the programme, policy or initiative evaluated	Y	4-5	Description of intervention placed in Background as this seemed more appropriate in providing the context for the protocol
10	Describe and justify the evaluation design	A description and justification of the evaluation design (i.e. the account of what was planned, done and why) should be included, at least in summary form or as an appendix, in the document which presents the main findings. If this is not done, the omission should be justified and a reference or link to the evaluation	Y	7-14	

		design given. It may also be useful to publish or make freely available (e.g. online on a website) any original evaluation design document or protocol, where they exist			
11	Data collection methods	Describe and justify the data collection methods – which ones were used, why and how they fed into developing, supporting, refuting or refining programme theory<br><br>Provide details of the steps taken to enhance the trustworthiness of data collection and documentation	Y	9-12	
12	Recruitment process and sampling strategy	Describe how respondents to the evaluation were recruited or engaged and how the sample contributed to the development, support, refutation or refinement of programme theory	Y	8	Sampling of sites, rather than individuals, is described; Recruitment will be described when reporting the results of the study
13	Data analysis	Describe in detail how data were analysed. This section should include information on the constructs that were identified, the process of	Y	12, 14	

For peer review only

		analysis, how the programme theory was further developed, supported, refuted and refined, and (where relevant) how analysis changed as the evaluation unfolded			
RESULTS					
14	Details of participants	Report (if applicable) who took part in the evaluation, the details of the data they provided and how the data was used to develop, support, refute or refine programme theory	NA		Protocol so no results to report
15	Main findings	Present the key findings, linking them to contexts, mechanisms and outcome configurations. Show how they were used to further develop, test or refine the programme theory	NA		Protocol so no results to report
DISCUSSION					
16	Summary of findings	Summarise the main findings with attention to the evaluation questions, purpose of the evaluation, programme theory and intended audience	NA		Protocol so no results to report
17	Strengths, limitations and future directions	Discuss both the strengths of the evaluation and its limitations. These should include (but need not be	NA		Discussion of the strengths and limitations will be covered when

For peer review only

		limited to): (1) consideration of all the steps in the evaluation processes; and (2) comment on the adequacy, trustworthiness and value of the explanatory insights which emerged<br><br>In many evaluations, there will be an expectation to provide guidance on future directions for the programme, policy or initiative, its implementation and/or design. The particular implications arising from the realist nature of the findings should be reflected in these discussions			reporting the results of the study
18	Comparison with existing literature	Where appropriate, compare and contrast the evaluation's findings with the existing literature on similar programmes, policies or initiatives	NA		Protocol so no results to compare with existing literature
19	Conclusion and recommendations	List the main conclusions that are justified by the analyses of the data. If appropriate, offer recommendations consistent with a realist approach	NA		Protocol so no results on which to base recommendations
20	Funding and conflict of interest	State the funding source (if any) for the evaluation, the role played by the	Y	16	

For peer review only

		funder (if any) and any conflicts of interests of the evaluators			

For peer review only

BMJ Open

How, in what contexts, and why do quality dashboards lead to improvements in care quality in acute hospitals? Protocol for a realist feasibility evaluation

Journal:	*BMJ Open*
Manuscript ID	bmjopen-2019-033208.R1
Article Type:	Protocol
Date Submitted by the Author:	21-Oct-2019
Complete List of Authors:	Randell, Rebecca; University of Leeds, School of Healthcare<br>Alvarado, Natasha; University of Leeds, School of Healthcare<br>McVey, Lynn; University of Leeds, School of Healthcare<br>Greenhalgh, Joanne; University of Leeds, Sociology and Social Policy<br>West, Robert; University of Leeds, Leeds Institute of Health Sciences<br>Farrin, Amanda; University of Leeds, Clinical Trials Research Unit<br>Gale, Chris; University of Leeds<br>Parslow, Roger; University of Leeds, Leeds Institute if Genetics, Health and Therapeutics<br>Keen, Justin; University of Leeds, Leeds Institute of Health Sciences<br>Elshehaly, Mai; University of Leeds, School of Computing<br>Ruddle, Roy; University of Leeds, School of Computing<br>Lake, Julia; Leeds Teaching Hospitals NHS Trust<br>Mamas, Mamas; Royal Stoke University Hospital<br>Feltbower, Richard; University of Leeds,<br>Dowding, Dawn; University of Manchester
**Primary Subject Heading**:	Health informatics
Secondary Subject Heading:	Health services research
Keywords:	AUDIT, Health informatics < BIOTECHNOLOGY & BIOINFORMATICS, Clinical audit < HEALTH SERVICES ADMINISTRATION & MANAGEMENT, Quality in health care < HEALTH SERVICES ADMINISTRATION & MANAGEMENT, QUALITATIVE RESEARCH

SCHOLARONE™
Manuscripts

I, the Submitting Author has the right to grant and does grant on behalf of all authors of the Work (as defined in the below author licence), an exclusive licence and/or a non-exclusive licence for contributions from authors who are: i) UK Crown employees; ii) where BMJ has agreed a CC-BY licence shall apply, and/or iii) in accordance with the terms applicable for US Federal Government officers or employees acting as part of their official duties; on a worldwide, perpetual, irrevocable, royalty-free basis to BMJ Publishing Group Ltd ("BMJ") its licensees and where the relevant Journal is co-owned by BMJ to the co-owners of the Journal, to publish the Work in this journal and any other BMJ products and to exploit all rights, as set out in our licence.

The Submitting Author accepts and understands that any supply made under these terms is made by BMJ to the Submitting Author unless you are acting as an employee on behalf of your employer or a postgraduate student of an affiliated institution which is paying any applicable article publishing charge ("APC") for Open Access articles. Where the Submitting Author wishes to make the Work available on an Open Access basis (and intends to pay the relevant APC), the terms of reuse of such Open Access shall be governed by a Creative Commons licence – details of these licences and which Creative Commons licence will apply to this Work are set out in our licence referred to above.

Other than as permitted in any relevant BMJ Author's Self Archiving Policies, I confirm this Work has not been accepted for publication elsewhere, is not being considered for publication elsewhere and does not duplicate material already published. I confirm all authors consent to publication of this Work and authorise the granting of this licence.

For peer review only

Title: How, in what contexts, and why do quality dashboards lead to improvements in care quality in acute hospitals? Protocol for a realist feasibility evaluation

Authors

Corresponding author: Rebecca Randell, School of Healthcare, University of Leeds, Leeds, UK, r.randell@leeds.ac.uk

Natasha Alvarado, of Healthcare, University of Leeds, Leeds, UK, n.alvarado@leeds.ac.uk

Lynn McVey, School of Healthcare, University of Leeds, Leeds, UK, l.mcvey@leeds.ac.uk

Joanne Greenhalgh, School of Sociology and Social Policy, University of Leeds, Leeds, UK, j.greenhalgh@leeds.ac.uk

Robert West, Leeds Institute of Health Sciences, University of Leeds, Leeds, UK, r.m.west@leeds.ac.uk

Amanda Farrin, Leeds Institute of Clinical Trials Research, University of Leeds, Leeds, UK, a.j.farrin@leeds.ac.uk

Chris P. Gale, School of Medicine, University of Leeds, Leeds, UK, c.p.gale@leeds.ac.uk

Roger Parslow, School of Medicine, University of Leeds, Leeds, UK, r.c.parslow@leeds.ac.uk

Justin Keen, Leeds Institute of Health Sciences, University of Leeds, Leeds, UK, j.keen@leeds.ac.uk

Mai Elshehaly, School of Computing, University of Leeds, Leeds, UK, m.h.elshehaly@leeds.ac.uk

Roy A. Ruddle, School of Computing, University of Leeds, Leeds, UK, r.a.ruddle@leeds.ac.uk

Julia Lake, Leeds Teaching Hospitals NHS Trust, Leeds, UK, julia.lake3@nhs.net

Mamas A. Mamas, Royal Stoke Hospital, Stoke on Trent, UK, mamasmamas1@yahoo.co.uk

Richard Feltbower, School of Medicine, University of Leeds, Leeds, UK, r.g.feltbower@leeds.ac.uk

Dawn Dowding, School of Health Sciences, University of Manchester, Manchester, UK, dawn.dowding@lmanchester.ac.uk

For peer review only - http://bmjopen.bmj.com/site/about/guidelines.xhtml

I, the Submitting Author has the right to grant and does grant on behalf of all authors of the Work (as defined in the below author licence), an exclusive licence and/or a non-exclusive licence for contributions from authors who are: i) UK Crown employees; ii) where BMJ has agreed a CC-BY licence shall apply, and/or iii) in accordance with the terms applicable for US Federal Government officers or employees acting as part of their official duties; on a worldwide, perpetual, irrevocable, royalty-free basis to BMJ Publishing Group Ltd ("BMJ") its licensees and where the relevant Journal is co-owned by BMJ to the co-owners of the Journal, to publish the Work in BMJ Open and any other BMJ products and to exploit all rights, as set out in our licence.

The Submitting Author accepts and understands that any supply made under these terms is made by BMJ to the Submitting Author unless you are acting as an employee on behalf of your employer or a postgraduate student of an affiliated institution which is paying any applicable article publishing charge ("APC") for Open Access articles. Where the Submitting Author wishes to make the Work available on an Open Access basis (and intends to pay the relevant APC), the terms of reuse of such Open Access shall be governed by a Creative Commons licence – details of these licences and which Creative Commons licence will apply to this Work are set out in our licence referred to above.

For peer review only

ABSTRACT

Introduction: National audits are used to monitor care quality and safety and are anticipated to reduce unexplained variations in quality by stimulating quality improvement. However, variation within and between providers in the extent to which they engage with national audits mean that the potential for national audit data to inform quality improvement is not being realised. This study aims to undertake a feasibility evaluation of QualDash, a quality dashboard designed to support clinical teams and managers to explore data from two national audits, the Myocardial Ischaemia National Audit Project (MINAP) and the Paediatric Intensive Care Audit Network (PICANet).

Methods and analysis: Realist evaluation, which involves building, testing, and refining theories of how an intervention is supposed to work, provides an overall framework. Realist hypotheses that describe how, in what contexts, and why QualDash is expected to provide benefit will be tested across five hospitals. A controlled interrupted time series analysis will investigate impacts of QualDash using key MINAP and PICANet measures. Ethnographic observations and interviews over 12 months will provide insight into contexts and mechanisms that lead to those impacts. Feasibility outcomes include the extent to which MINAP and PICANet data are used, data completeness in the audits, and the extent to which participants perceive QualDash to be useful and express the intention to continue using it after the study period.

Ethics and dissemination: The study has been approved by University of Leeds School of Healthcare Research Ethics Committee. Study results will provide an initial understanding of how, in what contexts, and why quality dashboards may lead to improvements in care quality. These will be disseminated to academic audiences, study participants, hospital IT departments, and national audits. If results show a trial of QualDash is feasible, we will disseminate the QualDash software through a stepped wedge cluster randomised trial.

Trial registration: ISRCTN18289782

Keywords: Dashboard, audit and feedback, quality improvement, realist evaluation

Word count: 4,078

For peer review only

ARTICLE SUMMARY

Strengths and limitations of this study

- This study combines a controlled interrupted time series study with a qualitative multi-site case study in order to provide an understanding of not only whether use of a quality dashboard leads to quality improvement but also how, in what contexts, and why.
- In addition to assessing the feasibility of a trial, the study will determine the components of QualDash to be preserved in a definitive trial, appropriate outcome measures, and the contexts in which a definitive trial should be undertaken.
- The study will contribute to understanding of how realist methods can contribute to feasibility studies and the design of trials.
- Issues of data quality may be a limitation of the CITS; data completeness, and whether this changes over the course of the study, will be assessed,

INTRODUCTION

National clinical audits (NCAs), which provide comparative data on the performance of healthcare providers, are one means by which health systems around the world monitor care quality and safety. In England, a programme of over 30 NCAs is managed by the Healthcare Quality Improvement Partnership (HQIP) and all healthcare providers that contribute to delivery of the National Health Service (NHS) are required to participate. Such audits are anticipated to reduce unexplained variations in healthcare quality by stimulating quality improvement (QI) [1 2]. While there is evidence of positive impacts of NCAs [3-5], variation within and between providers in the extent to which they engage with NCAs mean the potential for NCA data to inform QI is not being realised [6 7].

Quality dashboards are a form of audit and feedback (A&F) that provide visualisations of audit data with the aim of informing QI efforts [8]. Healthcare providers are increasingly using quality dashboards. For example, quality dashboard use has been reported in Canada [9], the UK [10], and the Netherlands [11]. While quality dashboards have been shown to have positive effects on some performance indicators [9], empirical evidence regarding their impact remains limited [12].

For peer review only

For peer review only - http://bmjopen.bmj.com/site/about/guidelines.xhtml

QualDash

QualDash is an interactive web-based quality dashboard designed to support clinical teams and managers to explore data from two national audits, the Myocardial Ischaemia National Audit Project (MINAP) and the Paediatric Intensive Care Audit Network (PICANet), for the purpose of QI (Fig. 1). Information used to inform design of QualDash was collected through interviews with 50 clinicians and managers across five NHS Trusts (providers) and four healthcare commissioners, observations of meetings where audit data are discussed, a workshop with NCA suppliers, and two co-design workshops with clinicians and managers from one Trust.

Fig. 1: Prototype of main dashboard view for the Paediatric Intensive Care Audit Network (using simulated data).

[Figure 1 should go approximately here]

The interviews revealed that use of NCA data is largely at the clinical team level, with more limited use at divisional and corporate (Board and sub-committees that report to the Board, such as Quality and Safety Committees) levels. At all levels, a key constraint in use of NCA data for QI is lack of access to timely data; there was consensus among interviewees that data should not be more than three months old. QualDash seeks to improve access to timely data, providing users with a means to visualise the data they collect for the NCAs, without having to wait for data to be returned to them from the NCAs. There is variation between Trusts in the extent to which NCA data are used, often related to resources, which in turn impacts on timeliness of data; Trusts that make greater use of NCA data tend to have local databases from which they can generate visualisations of the data (e.g. bar charts) and audit support staff who have the time and skills to be able to generate such visualisations. In contrast, where such resources are not available, Trusts rely on the NCA annual reports, where data may be 15 months old (e.g. one annual report published in June 2017 reported data from April 2015 to March 2016). QualDash provides visualisations of key metrics, each metric being represented within a 'QualCard' (Fig. 2), enabling Trusts to use NCA data for QI, regardless of existing resources. QualCards for MINAP and PICANet are listed in Table 1; while there is only one set of QualCards for PICANet, for MINAP an

For peer review only

additional QualCard is provided for teaching hospitals, as discussions with sites revealed that the metrics of interest are different between teaching hospitals and District General Hospitals (DGHs). Sites are also able to create additional QualCards, to reflect local priorities.

Fig. 2: Prototype of main dashboard view for the Paediatric Intensive Care Audit Network with the Mortality QualCard expanded (using simulated data).

[Figure 2 should go approximately here]

Table 1: QualCards

	Metric
MINAP – all sites	Mortality<br>Door (arrival in Accident and Emergency) to angiogram time<br>Gold standard drugs on discharge<br>Referral for cardiac rehabilitation<br>Acute use of aspirin
MINAP – teaching hospital specific	Call (by patient/relative to emergency services) to balloon (percutaneous coronary intervention) time
PICANet – all sites	Mortality<br>48 hour unplanned readmission<br>Bed days and accidental extubation<br>Specialty case mix<br>Data quality (number of records with a missing value)<br>Patient dependency

To load new data into QualDash, NCA data are either extracted from the site's database or downloaded from the NCA website and then fed to a small script (written in R), which in turn updates the dashboard. Users can add new data as often as they want, but at a minimum they will load data into QualDash at the same time as uploading to the NCAs (typically every three months).

The benefits perceived from using QualDash may vary between sites, with under-resourced sites that previously made little use of NCA data for QI perceiving greater impact than those that already have the means to use NCA data for QI. There are also constraints on use of NCA data for QI that it may be difficult for QualDash to address. For example, in some Trusts, clinical team members perceive that relevant managers will not agree to provide the resources necessary for QI initiatives, which reduces motivation to engage with NCA data and may affect the extent to which QualDash is used. However, QualDash provides means for visualisations to be downloaded and incorporated into presentations and

For peer review only - http://bmjopen.bmj.com/site/about/guidelines.xhtml

For peer review only

reports, which may support clinical teams in making a stronger case for QI initiatives. Another constraint on use of NCA data for QI relates to clinicians' trust in the quality of the data. Interviews revealed variations across sites in processes for ensuring data quality. However, some interviewees also suggested that having the means to make more use of NCA data via QualDash would motivate them to improve their processes for ensuring data quality, although this will be dependent on local resources.

In this paper, we describe the methods for a realist feasibility evaluation of QualDash. The study objectives are:

1. To understand how, in what contexts, and why use of QualDash leads to QI; and
2. To assess the feasibility of conducting a trial of QualDash.

As no checklists exist for reporting of realist evaluation protocols, in presenting this protocol we draw on the RAMESES II reporting standards for realist evaluations [13] (Additional file 1).

METHODS AND ANALYSIS

Study design

Use of theory is needed for design and evaluation of A&F interventions [14-16], and QI initiatives more generally [17-19]. This project draws on Realist Evaluation (RE), which involves building, testing, and refining theories about how an intervention is supposed to work [20]. These theories are expressed in the form of Context Mechanism Outcome (CMO) configurations, where C+M=O, reflecting the realist understanding that it is recipients' responses to the resources that an intervention provides (the intervention mechanisms) that determine the impact of the intervention, and such responses are highly influenced by context [21]. Consequently, RE seeks to answer not only the question of 'what works?' but 'what works for whom, in what circumstances, and why?' [22]. It is concerned with both intended and unintended outcomes. RE is recommended for studying QI [23] and has been used for studying the implementation and impact of large-scale QI programmes [24]. There is increasing interest in use of realist methods in feasibility evaluations [25-27].

We have drawn on a range of sources to develop CMO configurations which describe how, in what contexts, and why use of QualDash is anticipated to lead to QI (see Additional file 2). Data generated from the interviews, observations, and workshops described above have been essential to this, as have

For peer review only - http://bmjopen.bmj.com/site/about/guidelines.xhtml

discussions with the designers of QualDash (ME and RAR) who, drawing on their expertise in information visualisation, have their own literature-informed theories regarding why certain features of QualDash will provide benefit to users [28 29]. We have also drawn on substantive theories regarding how A&F lead to QI at the micro [30 31], meso [32], and macro level [33 34].

Data collection is designed to enable testing of the CMO configurations. Outcome data, in the form of key MINAP and PICANet measures, will be collected and analysed in a controlled interrupted time series (CITS) study, while a multi-site case study [35] will provide insight into the contexts and mechanisms that lead to those outcomes, as well as providing data on intermediate outcomes such as increased use of NCA data. A&F interventions, and QI interventions more generally, require longitudinal evaluation to allow sufficient time for staff to implement changes and incorporate them into their practice [36-38]. Similarly, evaluation of health IT (HIT) should allow time for staff to integrate the technology into their practices and evolve those practices to take advantage of the functionality offered by the technology [39]. Therefore, data will be collected over a 12 month period, from August 2019.

Public and patient involvement

A Lay Advisory Group has been established, which has contributed to the design of QualDash by reviewing the topic guide for the interviews that were conducted, providing their perspective on the findings of the interview study, and participating in the usability evaluation of QualDash. For the realist feasibility evaluation, they have provided advice on aspects to pay attention to when undertaking observations. They will contribute to analysis of a sample of the qualitative data, to provide a patient perspective. They will advise on dissemination of findings to relevant interest groups and will review outputs for comprehensibility.

Setting/context

QualDash will be evaluated in the five NHS acute Trusts in which the interview study that informed the design of QualDash was undertaken. Three Trusts are teaching hospitals that participate in both MINAP and PICANet and have been selected to ensure variation in key outcome measures (MINAP: 30-day mortality for patients hospitalised with ST-elevation myocardial infarction; PICANet: risk adjusted standardised mortality ratio). Two Trusts are DGHs that participate in MINAP but do not have a PICU

For peer review only - http://bmjopen.bmj.com/site/about/guidelines.xhtml

For peer review only

and so do not participate in PICANet. These have been selected to ensure variation in the same key MINAP measure.

Multi-site case study

In the multi-site case study, data will be collected through ethnographic observation and interviews. Ethnographic methods have been argued as essential for studying implementation of QI interventions [19] and introduction of HIT [40]. Ethnography is well suited to RE because it involves observing phenomena in context, supporting understanding of how context influences the response to an intervention [41]. We will follow the Biography of Artefacts approach [42], which is concerned with capturing how particular contexts and appropriations of a technology lead to different processes and generate different outcomes, a parallel to RE's concern with contexts, mechanisms, and outcomes [43]. It involves longitudinal 'strategic ethnography' [42], where data collection is guided by a provisional understanding of the moments and locales in which a technology and associated practices evolve [43].

Data collection

In the three teaching hospitals, we will undertake a minimum of 24 periods of observation per Trust, to be split across activities related to cardiology and the PICU, and in the two DGHs we will undertake a minimum of 12 periods of observation per Trust, to be spent observing activities related to cardiology. Each period of observation will be a minimum of four hours (total n=384 hours). While researchers will return to each Trust monthly, to understand how use of QualDash changes over time, more time will be spent in the first few months following the introduction of QualDash, because this is when users are most likely to engage with and explore the affordances of QualDash and establish new practices around it, generating information with implications for system enhancement [43]. Observations will be scheduled to take place at different times of day and on different days of the week, to ensure the account of what is observed is as complete and representative as possible [44].

At each case site, an initial phase of general observation will provide an opportunity for researchers to become familiar with the setting and for those in the setting to become familiar with the presence of the researchers. Following a previous study of dashboards [10], observations will be undertaken in clinical areas to understand clinical teams' working practices and capture 'corridor committees' where issues

For peer review only - http://bmjopen.bmj.com/site/about/guidelines.xhtml

of quality and safety are discussed more informally [45]. In the PICUs, initial observations will take place on the PICU, e.g. with the researchers positioning themselves by the nurses' station, as well as observing handovers, safety huddles, and ward rounds. Because activities related to cardiology tend to be more dispersed across hospitals, researchers will first shadow clinical team members (consultant cardiologists and acute chest pain nurses) to determine where it is most appropriate to conduct subsequent observations. These initial observations will also be used to record general details of the setting that may influence QualDash use, such as staffing levels and availability of computers.

After this initial phase, observation will be guided by the CMO configurations under investigation. In addition to observing formal meetings where quality and safety are discussed, predominantly at ward level but also at divisional and corporate level, observation will involve shadowing staff members as they undertake particular activities: collection and entry of NCA data, to see if and how this changes over time; accessing and interrogating NCA data, whether using QualDash or some other means; preparation of reports and/or presentations using NCA data, again whether using QualDash or some other means. Where visualisations from QualDash are incorporated into presentations and written reports, we will follow the path of those documents, to identify staff members who may not use QualDash directly but are receiving QualDash outputs. Attention will be paid to how, in what contexts, and why QualDash and QualDash outputs are used or not, understood in the context of broader practices and use of other sources of information for monitoring care quality, and how this changes over time. We will also follow local QI initiatives, recording data on, for example, when and how the need for the QI initiative was identified, contextual factors that appear to support and constrain its introduction, how the impact of the QI initiative is monitored, and other contextual factors that appear to influence the metric that the QI initiative is targeting. Researchers will record observations in fieldnotes, which will be written up in detail as soon after data collection as possible.

Brief interviews will be undertaken opportunistically during the course of conducting observations to clarify aspects of practice that are not immediately intelligible to an observer, with participant responses recorded in fieldnotes [46]. As data collection progresses, longer semi-structured interviews will be used to discuss revisions to our CMO configurations. These will be undertaken using a particular approach from RE, referred to as the teacher-learner cycle, whereby the theories under investigation are made

For peer review only - http://bmjopen.bmj.com/site/about/guidelines.xhtml

explicit to the interviewee so that the interviewee can use their experiences to refine the researcher's understanding [47]. Being concerned with the reasoning of intervention recipients, mechanisms are often not observable [21], so these longer interviews will also provide the opportunity to explore staff reasoning about QualDash. These longer interviews will be audio recorded and transcribed verbatim.

Logfiles are widely used to evaluate visualisation tools [48]. QualDash logfiles will record information about the user (job title, etc.), data used (audit, year), overall time spent using QualDash, time spent interacting with different QualCards (including new QualCards that have been created), functionality used, and whether QualDash visualisations were downloaded. In addition to providing data regarding extent of QualDash use, how QualDash is used and by whom, and how this changes over time, information from logfiles will be used to inform qualitative data collection (e.g. asking in interviews why participants use particular QualCards and not others and the motivation behind the creation of new QualCards).

At the end of the data collection period, we will ask participants to complete a questionnaire based on the Technology Acceptance Model, using well validated items that have been used in numerous evaluations of HIT [49], including dashboards [50]. This will provide participants' perceptions of the usefulness of QualDash and data on whether they intend to continue using QualDash after the study period.

Analysis

An iterative approach to data collection and analysis will be taken, to enable: ongoing testing and refinement of the CMO configurations; gathering of further data in light of such revisions; and refinement of QualDash in response to participants' feedback. Fieldnotes and interview transcripts will be entered into NVivo 11. Narrative analysis will be undertaken to develop a 'biography' of QualDash, which will describe use of QualDash and its outputs by a range of stakeholders at different levels (clinical team, divisional, and corporate) and the interconnections between them [10]. Narrative analysis is consistent with a realist approach due to its emphasis on preserving connections within the data, thereby helping to understand causality [51]. This analysis will be supplemented with analysis of the logfiles and

For peer review only

For peer review only - http://bmjopen.bmj.com/site/about/guidelines.xhtml

questionnaire data. Findings will be compared with the CMO configurations, to determine whether they support, refute, or suggest a revision or addition to the CMO configurations.

Controlled interrupted time series study

Interrupted time series studies provide a robust method of assessing the effect of an intervention and have been used to assess effectiveness of a variety of complex interventions [52]. In a CITS, the addition of a control group enhances causal inference because the presence of seasonal trends and other potential time-varying confounders can be assessed [53]. Data will be collected across the five Trusts, with two control Trusts per intervention Trust, providing a total of 10 control Trusts. Control Trusts will be matched according to their size and outcomes pre-intervention. Having more than one control site per intervention site increases power but, as the number of control sites per intervention site increases, quality of matching decreases. Therefore, we have chosen to have two control Trusts per intervention Trust to increase power while maintaining quality of the matching.

Given the study intention to determine the feasibility of and inform the design of a trial, a range of measures will be considered. Initially, we selected two process measures, one for MINAP and one for PICANet. For MINAP, we selected the composite process measure Cumulative Missed Opportunities for Care (CMOC). This has nine components (pre-hospital ECG, acute use of aspirin, timely perfusion, referral for cardiac rehabilitation, and prescription at hospital discharge of what are considered to be the gold standard drugs – aspirin, thienopyridine inhibitor, ACE-inhibitor, HMG-CoA reductase inhibitor, and beta blockers) and is inversely associated with mortality [54]. As some of these components, such as pre-hospital ECG, are outside the direct control of the Trust, we will also explore the impact of QualDash on the individual measures that make up CMOC. On the basis of the measures that cardiology clinicians described in the interviews as being important for measuring care quality, we will also look at the percentage of patients who receive an angiogram within 72 hours from first admission to hospital, which is part of the Best Practice Tariff financial incentive scheme, and, for those hospitals that provide percutaneous coronary intervention (PCI), the proportion of patients who have a door-to-balloon time (the time from arrival at the hospital to PCI) of less than 60 minutes. Our CMO configurations (Additional file 2) suggest improvement will be seen in measures if: clinical teams perceive them as being important indicators of care and/or they relate to financial incentives;

For peer review only - http://bmjopen.bmj.com/site/about/guidelines.xhtml

performance is not in line with expectations; they perceive the measure as being within their control; and the team is resourced to introduce QI initiatives in relation to these measures.

For PICANet, we initially selected use of non-invasive ventilation first for patients requiring ventilation, which has been shown to be associated with reduced mortality [55]. However, this was not raised as an area of concern in our interviews with PICU clinicians. On the basis of this and two additional considerations – it would require loading additional data into QualDash which would reduce the performance of QualDash in terms of speed and it requires computation of the data, while the focus of QualDash is on visualising the data – a QualCard has not been created for this metric. Therefore, we do not hypothesise that this measure will change, unless other sources of information, such as the PICANet annual report, draw a PICU team's attention to it. However, accidental extubation and unplanned readmission within 48 hours were identified in our interviews with PICU clinicians as being important indicators of care quality, so we will include these two measures in the CITS. On the basis of our CMO configurations (Additional file 2), we would expect to see an improvement in these measures in sites where performance is not in line with expectations, if the team is resourced to introduce QI initiatives in relation to these measures.

Sample size considerations

A CITS study requires data for a minimum of three time points pre-intervention and three time points post-intervention and must also allow for any seasonal effect on the outcomes [56]. Monthly data will be obtained for 24 months pre-intervention and 12 months post-intervention. Consequently, for each intervention Trust, there will be 72 data points prior to introduction (24 for the intervention Trust and 48 for the control Trusts) and 36 data points post intervention (12 for the intervention Trust and 24 for the control Trusts). Sample size calculations were undertaken based on our two initial measures, CMOC for MINAP and use of non-invasive ventilation first for patients requiring ventilation for PICANet; full details are provided in Additional file 3.

Analysis

Monthly MINAP and PICANet data will be extracted to spreadsheets for analysis with R software [57]. For both NCAs, each outcome will be regressed upon time and the intervention. The time component

For peer review only - http://bmjopen.bmj.com/site/about/guidelines.xhtml

will include a seasonal effect (quarterly effect) and will allow for a (linear) time trend. To account for clustering of monthly observations within hospitals, a random intercept will be fitted, although a fixed effect for hospital as a sensitivity analysis will be explored. Although the intervention is abrupt, its impact may well be 'phased in' over a few months, perhaps three. The timing of the bedding in of the intervention will be reported from the multi-site case study. Then a partial effect can be considered for this period with the interaction effect stepping up in a linear fashion.

Results of the CITS analysis will be incorporated into the biography of QualDash, the analysis of the data from the multi-site case study describing how contextual factors shape the evolution of practices around QualDash and how this leads to the resulting outcome pattern.

Trial feasibility assessment and design

Our trial progression criteria are: (i) the number of people who engage with either MINAP or PICANet data (via QualDash or some other means) is the same or higher than the number of people who engaged with either MINAP or PICANet data prior to QualDash's introduction; (ii) data completeness in the national audit improves or remains the same; (iii) 50% or more of participants in the questionnaire survey perceive QualDash to be useful and express the intention to continue using it after the study period. Criteria (i) and (ii) are concerned with ensuring the intervention does not have unintended negative consequences which would affect success of the intervention. Criterion (ii) is also concerned with feasibility of outcome assessment. Criterion (iii) is concerned with acceptability and uptake of the intervention, and therefore has implications for recruitment to a trial, as well as being concerned with participants' perceptions of the impact of QualDash on care. While not formally assessed as part of the progression criteria, the impact of QualDash on care as identified in the CITS will be considered in determining whether a future trial is justified. A traffic light system will be used to determine if a trial is feasible (green), feasible with modifications to QualDash (amber), or not feasible (red) [58 59].

If the results show a trial of QualDash is feasible, we will design a stepped wedge cluster randomised trial. Data from the CITS will be used to inform the selection of NCAs to be included in the trial (MINAP and/or PICANet) and will provide information about variability of outcomes and about how long a trial intervention period would need to be. Findings from the multi-site case study will be used to inform the

For peer review only - http://bmjopen.bmj.com/site/about/guidelines.xhtml

selection of categories of user to be included in the trial and, associated with this, the level of randomisation (Trust, hospital, or ward). Using the understanding of the relationship between contexts, mechanisms, and outcomes provided by the study, we will identify QualDash components associated with mechanisms that produce the desired outcomes in order for them to be preserved in the trial, while other components can be adapted to suit the local context.

ETHICS AND DISSEMINATION

Ethics approval has been received from the University of Leeds School of Healthcare Research Ethics Committee (Approval no.HREC16-044). Written consent will be obtained from participants for interviews and for meeting observations.

Study results will provide initial understanding of how and in what contexts quality dashboards may lead to improvements in care quality. We will disseminate these results to academic audiences, study participants, hospital IT departments, and NCAs. If we progress to a trial, in addition to providing further understanding of the impact of quality dashboards on care quality, this will result in wider dissemination of the QualDash software.

Acknowledgements

This research is funded by the National Institute for Health Research (NIHR) Health Services and Delivery Research (HS&DR) Programme (project number 16/04/06). The views and opinions expressed are those of the presenter and do not necessarily reflect those of the HS&DR programme, NIHR, NHS or the Department of Health.

Data statement

The qualitative data gathered during the course of this evaluation will be kept until June 2030 and can be accessed by other researchers during this time, subject to the necessary ethical approvals being obtained. Requests for access to this data should be addressed to the corresponding author.

For peer review only

Authors' contributions

RR is Principal Investigator for the study. She conceived, designed, and secured funding for the study in collaboration with JG, RW, AF, CPG, RP, JK, RAR, JL, MM, and DD. NA and LM led the qualitative data collection and analysis that informed the design of QualDash and the design of evaluation. ME developed the QualDash software and contributed to the design of the evaluation. RP and RF provided data for the testing of QualDash and provided significant feedback on its design. All authors provided input into various aspects of the evaluation design and revised drafts of the protocol. RR led the writing of this protocol manuscript. All authors read and approved the final manuscript.

Funding statement

This research is funded by the National Institute for Health Research (NIHR) Health Services and Delivery Research (HS&DR) Programme (project number 16/04/06).

Competing interests statement

Chris Gale is a member of the MINAP Academic and Steering Groups. Richard Feltbower is Principal Investigator for PICANet and Roger Parslow was previously Principal Investigator for PICANet. The authors have no other competing interests to declare.

Additional files

Additional file 1: Checklist of RAMESES II reporting standards for realist evaluations (PDF)

Additional file 2: Context Mechanism Outcome configurations to be tested in the realist feasibility evaluation (PDF)

Additional file 3: Sample size calculations for controlled interrupted time series (PDF)

REFERENCES

1. Phekoo KJ, Clements J, Bell D. National Clinical Audit Quality Assessment - Overview of the self-assessment survey: "audit of audits". London: Healthcare Quality Improvement Partnership, 2014.
2. Miller T, Leatherman S. The National Quality Forum: a'me-too'or a breakthrough in quality measurement and reporting? Health Aff. (Millwood) 1999;**18**(6):233-37.
3. Birkhead JS, Walker L, Pearson M, Weston C, Cunningham AD, Rickards AF. Improving care for patients with acute coronary syndromes: initial results from the National Audit of Myocardial Infarction Project (MINAP). Heart 2004;**90**(9):1004-09.

For peer review only - http://bmjopen.bmj.com/site/about/guidelines.xhtml

4. Bridgewater B, Grayson AD, Brooks N, et al. Has the publication of cardiac surgery outcome data been associated with changes in practice in northwest England: an analysis of 25 730 patients undergoing CABG surgery under 30 surgeons over eight years. Heart 2007;**93**(6):744-48.

5. Neuburger J, Currie C, Wakeman R, et al. The Impact of a National Clinician-led Audit Initiative on Care and Mortality after Hip Fracture in England: An External Evaluation using Time Trends in Non-audit Data. Med. Care 2015;**53**(8):686-91.

6. Allwood D. Engaging Clinicians in Quality Improvement through National Clinical Audit. London: Healthcare Quality Improvement Partnership, 2014.

7. Taylor A, Neuburger J, Walker K, Cromwell D, Groene O. How is feedback from national clinical audits used? Views from English National Health Service trust audit leads. J. Health Serv. Res. Policy 2016;**21**(2):91-100.

8. Pauwels K, Ambler T, Clark BH, et al. Dashboards as a Service Why, What, How, and What Research Is Needed? Journal of Service Research 2009;**12**(2):175-89.

9. Weiss D, Dunn SI, Sprague AE, et al. Effect of a population-level performance dashboard intervention on maternal-newborn outcomes: an interrupted time series study. BMJ Quality & Safety 2018;**27**(6):425-36.

10. Keen J, Nicklin E, Long A, et al. Quality and safety between ward and board: a biography of artefacts study. Health Services and Delivery Research 2018;**6**(22).

11. Weggelaar-Jansen AMJWM, Broekharst DSE, de Bruijne M. Developing a hospital-wide quality and safety dashboard: a qualitative research study. BMJ Quality & Safety 2018.

12. Dowding D, Randell R, Gardner P, et al. Dashboards for improving patient care: Review of the literature. International Journal of Medical Informatics 2015;**84**(2):87-100.

13. Wong G, Westhorp G, Manzano A, Greenhalgh J, Jagosh J, Greenhalgh T. RAMESES II reporting standards for realist evaluations. BMC Med. 2016;**14**(1):96.

14. Foy R, Eccles MP, Jamtvedt G, Young J, Grimshaw JM, Baker R. What do we know about how to do audit and feedback? Pitfalls in applying evidence from a systematic review. BMC Health Serv. Res. 2005;**5**:50.

15. Ivers NM, Grimshaw JM, Jamtvedt G, et al. Growing Literature, Stagnant Science? Systematic Review, Meta-Regression and Cumulative Analysis of Audit and Feedback Interventions in Health Care. J. Gen. Intern. Med. 2014;**29**(11):1534-41.

16. Ivers NM, Sales A, Colquhoun H, et al. No more 'business as usual' with audit and feedback interventions: towards an agenda for a reinvigorated intervention. Implementation Science 2014;**9**(1):1-8.

17. Grol RPTM, Bosch MC, Hulscher MEJL, Eccles MP, Wensing M. Planning and Studying Improvement in Patient Care: The Use of Theoretical Perspectives. Milbank Q. 2007;**85**(1):93-138.

18. Davidoff F, Dixon-Woods M, Leviton L, Michie S. Demystifying theory and its use in improvement. BMJ Quality & Safety 2015.

19. Dixon-Woods M, Bosk CL, Aveling EL, Goeschel CA, Pronovost PJ. Explaining Michigan: Developing an Ex Post Theory of a Quality Improvement Program. Milbank Q. 2011;**89**(2):167-205.

20. Pawson R, Tilley N. *Realistic Evaluation*. London: SAGE Publications, 1997.

21. Dalkin S, Greenhalgh J, Jones D, Cunningham B, Lhussier M. What's in a mechanism? Development of a key concept in realist evaluation. Implementation Science 2015;**10**(1):49.

22. Pawson R. *The science of evaluation: a realist manifesto*. London: SAGE, 2013.

23. Robert G, Fulop N. The role of context in successful improvement. Perspectives on context. A selection of essays considering the role of context in successful quality improvement. London: Health Foundation 2014;**31**.

24. Greenhalgh T, Humphrey C, Hughes J, Macfarlane F, Butler C, Pawson R. How Do You Modernize a Health Service? A Realist Evaluation of Whole-Scale Transformation in London. Milbank Q. 2009;**87**(2):391-416.

For peer review only

For peer review only - http://bmjopen.bmj.com/site/about/guidelines.xhtml

25. Fletcher A, Jamal F, Moore G, Evans RE, Murphy S, Bonell C. Realist complex intervention science: Applying realist principles across all phases of the Medical Research Council framework for developing and evaluating complex interventions. Evaluation 2016;**22**(3):286-303.
26. Brand SL, Quinn C, Pearson M, et al. Building programme theory to develop more adaptable and scalable complex interventions: Realist formative process evaluation prior to full trial. Evaluation 2019;**25**(2):149-70.
27. Randell R, Honey S, Hindmarsh J, et al. A realist process evaluation of robot-assisted surgery: integration into routine practice and impacts on communication, collaboration and decision-making. Health Services and Delivery Research 2017;**5**(20).
28. Andrienko N, Andrienko G. *Exploratory analysis of spatial and temporal data: a systematic approach*: Springer Science & Business Media, 2006.
29. Brehmer M, Munzner T. A multi-level typology of abstract visualization tasks. IEEE transactions on visualization and computer graphics 2013;**19**(12):2376-85.
30. Hysong SJ, Best RG, Pugh JA. Audit and feedback and clinical practice guideline adherence: Making feedback actionable. Implementation Science 2006;**1**(1):1-10.
31. Sapyta J, Riemer M, Bickman L. Feedback to clinicians: Theory, research, and practice. J. Clin. Psychol. 2005;**61**(2):145-53.
32. Van Helden GJ, Tillema S. In Search of a Benchmarking Theory for the Public Sector. Financial Accountability & Management 2005;**21**(3):337-61.
33. Scott RW. *Institutions and Organizations*. 2nd Edition ed. London: Sage, 2001.
34. Furusten S. *Institutional Theory and Organizational Change*. Cheltenham, UK: Edward Elgar, 2013.
35. Yin RK. *Case study research: design and methods*. 3rd ed. Thousand Oaks, California: SAGE, 2003.
36. Øvretveit J, Gustafson D. Evaluation of quality improvement programmes. Quality and Safety in Health Care 2002;**11**(3):270-75.
37. Ramsay AIG, Turner S, Cavell G, et al. Governing patient safety: lessons learned from a mixed methods evaluation of implementing a ward-level medication safety scorecard in two English NHS hospitals. BMJ Quality & Safety 2013;**23**:136-46.
38. Benn J, Burnett S, Parand A, Pinto A, Iskander S, Vincent C. Studying large-scale programmes to improve patient safety in whole care systems: Challenges for research. Soc. Sci. Med. 2009;**69**(12):1767-76.
39. Hyysalo S. *Health technology development and use: from practice-bound imagination to evolving impacts*. New York: Routledge, 2010.
40. Greenhalgh T, Swinglehurst D. Studying technology use as social practice: the untapped potential of ethnography. BMC Med. 2011;**9**(1):45.
41. Dainty KN, Golden BR, Hannam R, et al. A realist evaluation of value-based care delivery in home care: The influence of actors, autonomy and accountability. Soc. Sci. Med. 2018;**206**:100-09.
42. Pollock N, Williams R. *Software and organisations: The biography of the enterprise-wide system or how SAP conquered the world*. New York: Routledge, 2008.
43. Pollock N, Williams R. e-Infrastructures: How Do We Know and Understand Them? Strategic Ethnography and the Biography of Artefacts. Computer Supported Cooperative Work (CSCW) 2010;**19**(6):521-56.
44. Hammersley M, Atkinson P. *Ethnography: principles in practice*. London: Routledge, 1995.
45. Waring J. Adaptive regulation or governmentality: patient safety and the changing regulation of medicine. Sociol. Health Illn. 2007;**29**(2):163-79.
46. Goodwin D, Pope C, Mort M, Smith A. Access, boundaries and their effects: legitimate participation in anaesthesia. Sociol. Health Illn. 2005;**27**(6):855-71.
47. Pawson R. Theorizing the Interview. The British Journal of Sociology 1996;**47**(2):295-314.
48. Harrison DG, Efford ND, Fisher QJ, Ruddle RA. PETMiner—A Visual Analysis Tool for Petrophysical Properties of Core Sample Data. IEEE transactions on visualization and computer graphics 2017;**24**(5):1728-41.

For peer review only - http://bmjopen.bmj.com/site/about/guidelines.xhtml

49. Holden RJ, Karsh B-T. The Technology Acceptance Model: Its past and its future in health care. Journal of Biomedical Informatics 2010;**43**(1):159-72.
50. Lee K, Jung SY, Hwang H, et al. A novel concept for integrating and delivering health information using a comprehensive digital dashboard: An analysis of healthcare professionals' intention to adopt a new system and the trend of its real usage. Int. J. Med. Inform. 2017;**97**:98-108.
51. Maxwell JA. *A realist approach for qualitative research*. London: SAGE Publications, 2012.
52. Hu Y. Stepped wedge, natural experiments and interrupted time series analysis designs. In: Richards DA, Hallberg IR, eds. Complex interventions in health: An overview of research methods. Abingdon: Routledge, 2015:200-12.
53. Handley MA, Lyles CR, McCulloch C, Cattamanchi A. Selecting and Improving Quasi-Experimental Designs in Effectiveness and Implementation Research. Annu. Rev. Public Health 2018;**39**(1):5-25.
54. Simms AD, Baxter PD, Cattle BA, et al. An assessment of composite measures of hospital performance and associated mortality for patients with acute myocardial infarction. Analysis of individual hospital performance and outcome for the National Institute for Cardiovascular Outcomes Research (NICOR). European Heart Journal: Acute Cardiovascular Care 2013;**2**(1):9-18.
55. Morris JV, Ramnarayan P, Parslow RC, Fleming SJ. Outcomes for Children Receiving Noninvasive Ventilation as the First-Line Mode of Mechanical Ventilation at Intensive Care Admission: A Propensity Score-Matched Cohort Study. Crit. Care Med. 2017.
56. Cochrane Effective Practice and Organisation of Care Group (EPOC). What study designs should be inlcuded in an EPOC review and what should they be called? Oslo: Norwegian Knowledge Centre for the Health Services, 2013.
57. R Core Team. R: A language and environment for statistical computing. Vienna, Austria: R Foundation for Statistical Computing, 2014.
58. Bugge C, Williams B, Hagen S, et al. A process for Decision-making after Pilot and feasibility Trials (ADePT): development following a feasibility study of a complex intervention for pelvic organ prolapse. Trials 2013;**14**(1):353.
59. Thabane L, Ma J, Chu R, et al. A tutorial on pilot studies: the what, why and how. BMC Med. Res. Methodol. 2010;**10**(1):1-10.

For peer review only

For peer review only - http://bmjopen.bmj.com/site/about/guidelines.xhtml

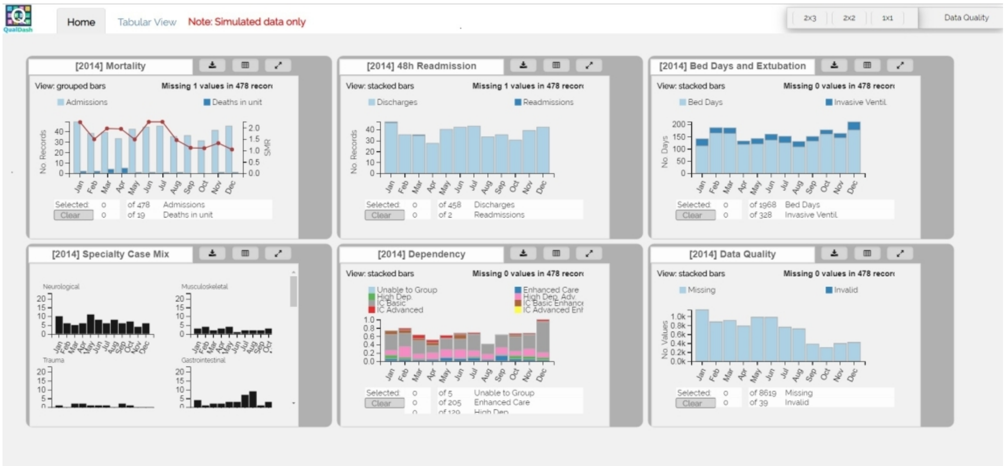

Fig. 1: Prototype of main dashboard view for the Paediatric Intensive Care Audit Network (using simulated data).

161x74mm (300 x 300 DPI)

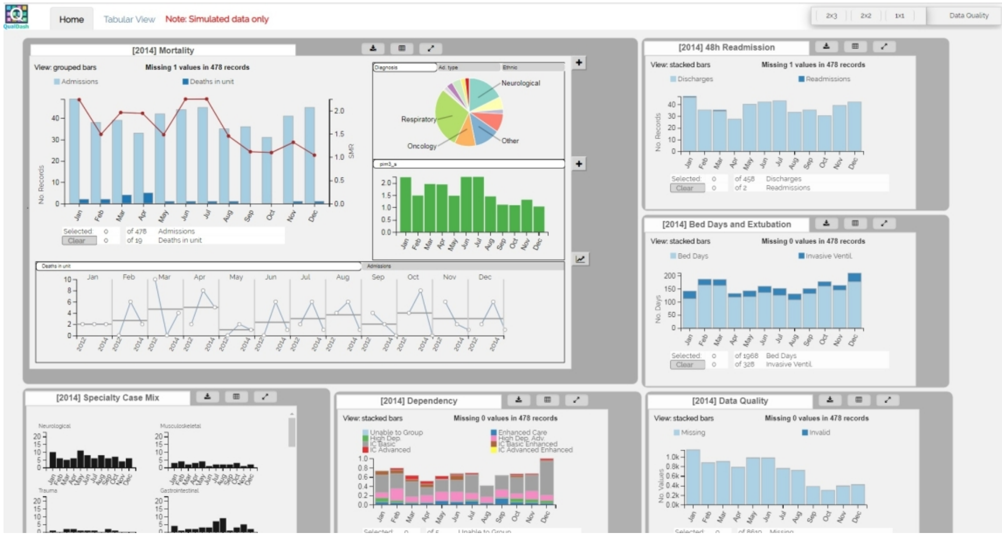

Fig. 2: Prototype of main dashboard view for the Paediatric Intensive Care Audit Network with the Mortality QualCard expanded (using simulated data).

161x85mm (300 x 300 DPI)

RAMESES II reporting standards for realist evaluations

How, in what contexts, and why do quality dashboards lead to improvements in care quality in acute hospitals? Protocol for a realist feasibility evaluation			Reported in document Y/N/NA	Page(s) in document	Comment
1		In the title, identify the document as a realist evaluation	Y	1	
SUMMARY OR ABSTRACT					
2		Journal articles will usually require an abstract, while reports and other forms of publication will usually benefit from a short summary. The abstract or summary should include brief details on: the policy, programme or initiative under evaluation; programme setting; purpose of the evaluation; evaluation question(s) and/or objective(s); evaluation strategy; data collection, documentation and analysis methods; key findings and conclusions<br><br>Where journals require it and the nature of the study is appropriate, brief details of respondents to the	Y	3	

For peer review only

		evaluation and recruitment and sampling processes may also be included<br><br>Sufficient detail should be provided to identify that a realist approach was used and that realist programme theory was developed and/or refined			
INTRODUCTION					
3	Rationale for evaluation	Explain the purpose of the evaluation and the implications for its focus and design	Y	7	
4	Programme theory	Describe the initial programme theory (or theories) that underpin the programme, policy or initiative	Y	8 and Additional file 2	Placed in body of article, rather than Introduction, as more appropriate for protocol
5	Evaluation questions, objectives and focus	State the evaluation question(s) and specify the objectives for the evaluation. Describe whether and how the programme theory was used to define the scope and focus of the evaluation	Y	7	
6	Ethical approval	State whether the realist evaluation required and has gained ethical approval from the relevant	Y	15	

For peer review only

		authorities, providing details as appropriate. If ethical approval was deemed unnecessary, explain why			
METHODS					
7	Rationale for using realist evaluation	Explain why a realist evaluation approach was chosen and (if relevant) adapted	Y	7-8	
8	Environment surrounding the evaluation	Describe the environment in which the evaluation took place	Y	8-9	
9	Describe the programme policy, initiative or product evaluated	Provide relevant details on the programme, policy or initiative evaluated	Y	5-7	Description of intervention placed in Introduction as this seemed more appropriate in providing the context for the protocol
10	Describe and justify the evaluation design	A description and justification of the evaluation design (i.e. the account of what was planned, done and why) should be included, at least in summary form or as an appendix, in the document which presents the main findings. If this is not done, the omission should be justified and a reference or link to the evaluation	Y	7-14	

For peer review only

		design given. It may also be useful to publish or make freely available (e.g. online on a website) any original evaluation design document or protocol, where they exist			
11	Data collection methods	Describe and justify the data collection methods – which ones were used, why and how they fed into developing, supporting, refuting or refining programme theory<br>Provide details of the steps taken to enhance the trustworthiness of data collection and documentation	Y	9-11	
12	Recruitment process and sampling strategy	Describe how respondents to the evaluation were recruited or engaged and how the sample contributed to the development, support, refutation or refinement of programme theory	Y	8-9	Sampling of sites, rather than individuals, is described; Recruitment will be described when reporting the results of the study
13	Data analysis	Describe in detail how data were analysed. This section should include information on the constructs that were identified, the process of	Y	11-12, 13-14	

For peer review only

		analysis, how the programme theory was further developed, supported, refuted and refined, and (where relevant) how analysis changed as the evaluation unfolded			
RESULTS					
14	Details of participants	Report (if applicable) who took part in the evaluation, the details of the data they provided and how the data was used to develop, support, refute or refine programme theory	NA		Protocol so no results to report
15	Main findings	Present the key findings, linking them to contexts, mechanisms and outcome configurations. Show how they were used to further develop, test or refine the programme theory	NA		Protocol so no results to report
DISCUSSION					
16	Summary of findings	Summarise the main findings with attention to the evaluation questions, purpose of the evaluation, programme theory and intended audience	NA		Protocol so no results to report
17	Strengths, limitations and future directions	Discuss both the strengths of the evaluation and its limitations. These should include (but need not be	4		Strengths and limitations of study design

For peer review only

		limited to): (1) consideration of all the steps in the evaluation processes; and (2) comment on the adequacy, trustworthiness and value of the explanatory insights which emerged<br><br>In many evaluations, there will be an expectation to provide guidance on future directions for the programme, policy or initiative, its implementation and/or design. The particular implications arising from the realist nature of the findings should be reflected in these discussions			
18	Comparison with existing literature	Where appropriate, compare and contrast the evaluation's findings with the existing literature on similar programmes, policies or initiatives	NA		Protocol so no results to compare with existing literature
19	Conclusion and recommendations	List the main conclusions that are justified by the analyses of the data. If appropriate, offer recommendations consistent with a realist approach	NA		Protocol so no results on which to base recommendations
20	Funding and conflict of interest	State the funding source (if any) for the evaluation, the role played by the	Y	16	

For peer review only

		funder (if any) and any conflicts of interests of the evaluators			

For peer review only

How, in what contexts, and why do quality dashboards lead to improvements in care quality in acute hospitals? Protocol for a realist feasibility evaluation (Randell et al.)

Additional file 2

#	Context	+	Mechanism: Resource	Mechanism: Response	=	Outcome
**1.**	Teams previously constrained in their ability to use NCA data for monitoring service performance because data not considered to be timely, accurate, and/or complete	+	QualDash offers easy access to key metrics	Teams are able to see whether the data displayed are timely, accurate, and/or complete and, where they are not, adjust their data collection processes in order to benefit from QualDash	=	Improvement in data quality in terms of timeliness, accuracy, and completeness – as data quality improves, use of QualDash increases
				Teams use QualDash to embed NCA data within their monitoring processes e.g. in clinical governance meetings where data is presented visually via screens.	=	Increased routine use of NCA data in performance monitoring, providing opportunities for its use in quality improvement
**2.**	Teams previously using NCA data to monitor service performance routinely by extracting raw data and producing reports for review in meetings and by individuals	+	QualDash visualises key metrics in ways that clearly show whether service performance is within an expected range and provides functions to interrogate that data	Teams use QualDash to facilitate their existing processes for monitoring service performance using NCA data	=	Reduced time spent in accessing, and preparing visualisations of, NCA data

For peer review only

For peer review only - http://bmjopen.bmj.com/site/about/guidelines.xhtml

#	Context	+	Mechanism		=	Outcome
			**Resource**	**Response**		
**3.**	Teams who want to use NCA data but were previously constrained by data quality and existing systems did not provide functions to easily access and interact with the data	**+**	QualDash provides functions that enable users to interact with NCA data and explore relationships between variables	Teams will use these functions to interrogate anomalies in the data, which will help them to understand what has impacted performance, thereby enabling them to identify appropriate strategies for improving performance	**=**	Introduction of QI initiatives in relation to metrics that teams consider important and where performance is not in line with expectations<br><br>Over time, improvement in metrics that QI initiatives target
**4.**	Performance in key metrics, such as the Best Practice Tariff, is in line with expectations<br><br>Relevant audit/IT support staff have time and willingness to support use of QualDash	**+**	QualDash offers teams the ability to quickly and easily add new QualCards (within NCA parameters)	Teams add new QualCards to be able to monitor and interrogate metrics they have chosen as important	**=**	Introduction of QI initiatives in relation to metrics shown on new QualCards when performance is not in line with expectations<br><br>Over time, improvement in metrics that QI initiatives target

For peer review only - http://bmjopen.bmj.com/site/about/guidelines.xhtml

#	Context	+	Mechanism		=	Outcome
			Resource	Response		
**5.**	Teams who previously did not, or were not able to, monitor key metrics routinely<br><br>Performance is not in line with expectations in key metrics<br><br>Teams are resourced to make practice changes	**+**	QualDash provides quick and easy access to key metrics	Teams will become aware of discrepancies between performance and targets in key metrics, which they will take action to address	**=**	Introduction of QI initiatives in relation to key metrics<br><br>Over time, improvement in those metrics
**6.**	Teams are asked to produce reports and recommendations for managers and other groups about service performance, e.g. at the time of publication of NCA annual report	**+**	QualDash offers easy access to NCA data and visualisations that can be exported into reports	Teams will use QualDash to produce performance reports requested by other groups	**=**	Reduced time spent in report preparation<br><br>Increased use of NCA data at divisional and corporate levels via outputs produced by QualDash<br><br>Over time, use of QualDash at divisional and/or corporate levels, due to increased awareness of NCA data

For peer review only - http://bmjopen.bmj.com/site/about/guidelines.xhtml

#	Context	+	Mechanism		=	Outcome
			**Resource**	**Response**		
**7.**	Teams receive data requests from service managers	**+**	QualDash can be easily accessed via the web by multiple users	Service managers will use QualDash to access the information they need quickly and easily	**=**	Streamlines the use of NCA data for clinical managers<br><br>Reduced time spent by audit support staff/clinical team in producing data reports for managers
**8.**	Teams need to evidence their performance to managers and other groups in order to support a case for practice change e.g. in business meetings with managers or in the NCA annual report summary	**+**	QualDash visualises performance metrics, which can also be exported into reports and presentations	Teams will use these functions to evidence service performance, in order to convince other Trust groups that change is needed	**=**	Other Trust groups, who are able to offer additional resource to teams, are convinced of the need for change based on the evidence provided. However, this is likely to be where those outputs are clearly associated with Trust priorities, e.g. relating to Trust reputation or avoiding penalties/gaining incentives.

For peer review only

How, in what contexts, and why do quality dashboards lead to improvements in care quality in acute hospitals? Protocol for a realist feasibility evaluation (Randell et al.)
Additional file 2

The average CMOC for patients on a ward will be averaged for each month, so that there are 36 observations clustered within each of 15 hospitals. Taking the intra-class correlation to be 0.15, this yields a design effect of 6.25. Hence the effective number of observations is 15*36/6.25=86.4. Using Cohen's approach to sample size calculation means an effect size of 0.17 can be estimated with 80% given that there are six parameters in the model (including the coefficient for QualDash). Converting this to the percentage of variation that can be explained by the model, this yields 20.5%. Translating this back to CMOC, currently 49.6% of patients are discharged from hospital without missing any of the nine opportunities for care, and we would be powered at the 80% level to detect an improvement from an average of 8.33 opportunities achieved to 8.46. Thus our study has good power to detect small but meaningful clinical improvements. For PICANet, 10% of the admitted population receive non-invasive ventilation first [1]. On average there are 5.25 ventilation cases per month per hospital. With a further design effect from patients clustered within hospitals, based on the reported intraclass correlation coefficient of 0.065 giving a design effect of 1.276, the actual anticipated number of patients is 1701 giving an effective number of 213: 71 exposed to QualDash and 142 controls. This yields 80% power to detect a change from 32% to 53%.

References

1. Morris JV, Ramnarayan P, Parslow RC, Fleming SJ. Outcomes for Children Receiving Noninvasive Ventilation as the First-Line Mode of Mechanical Ventilation at Intensive Care Admission: A Propensity Score-Matched Cohort Study. Crit. Care Med. 2017.

For peer review only

BMJ Open

How, in what contexts, and why do quality dashboards lead to improvements in care quality in acute hospitals? Protocol for a realist feasibility evaluation

Journal:	*BMJ Open*
Manuscript ID	bmjopen-2019-033208.R2
Article Type:	Protocol
Date Submitted by the Author:	13-Dec-2019
Complete List of Authors:	Randell, Rebecca; University of Leeds, School of Healthcare<br>Alvarado, Natasha; University of Leeds, School of Healthcare<br>McVey, Lynn; University of Leeds, School of Healthcare<br>Greenhalgh, Joanne; University of Leeds, Sociology and Social Policy<br>West, Robert; University of Leeds, Leeds Institute of Health Sciences<br>Farrin, Amanda; University of Leeds, Clinical Trials Research Unit<br>Gale, Chris; University of Leeds<br>Parslow, Roger; University of Leeds, Leeds Institute if Genetics, Health and Therapeutics<br>Keen, Justin; University of Leeds, Leeds Institute of Health Sciences<br>Elshehaly, Mai; University of Leeds, School of Computing<br>Ruddle, Roy; University of Leeds, School of Computing<br>Lake, Julia; Leeds Teaching Hospitals NHS Trust<br>Mamas, Mamas; Royal Stoke University Hospital<br>Feltbower, Richard; University of Leeds,<br>Dowding, Dawn; University of Manchester
**Primary Subject Heading**:	Health informatics
Secondary Subject Heading:	Health services research
Keywords:	AUDIT, Health informatics < BIOTECHNOLOGY & BIOINFORMATICS, Clinical audit < HEALTH SERVICES ADMINISTRATION & MANAGEMENT, Quality in health care < HEALTH SERVICES ADMINISTRATION & MANAGEMENT, QUALITATIVE RESEARCH

SCHOLARONE™
Manuscripts

I, the Submitting Author has the right to grant and does grant on behalf of all authors of the Work (as defined in the below author licence), an exclusive licence and/or a non-exclusive licence for contributions from authors who are: i) UK Crown employees; ii) where BMJ has agreed a CC-BY licence shall apply, and/or iii) in accordance with the terms applicable for US Federal Government officers or employees acting as part of their official duties; on a worldwide, perpetual, irrevocable, royalty-free basis to BMJ Publishing Group Ltd ("BMJ") its licensees and where the relevant Journal is co-owned by BMJ to the co-owners of the Journal, to publish the Work in this journal and any other BMJ products and to exploit all rights, as set out in our licence.

The Submitting Author accepts and understands that any supply made under these terms is made by BMJ to the Submitting Author unless you are acting as an employee on behalf of your employer or a postgraduate student of an affiliated institution which is paying any applicable article publishing charge ("APC") for Open Access articles. Where the Submitting Author wishes to make the Work available on an Open Access basis (and intends to pay the relevant APC), the terms of reuse of such Open Access shall be governed by a Creative Commons licence – details of these licences and which Creative Commons licence will apply to this Work are set out in our licence referred to above.

Other than as permitted in any relevant BMJ Author's Self Archiving Policies, I confirm this Work has not been accepted for publication elsewhere, is not being considered for publication elsewhere and does not duplicate material already published. I confirm all authors consent to publication of this Work and authorise the granting of this licence.

For peer review only

Title: How, in what contexts, and why do quality dashboards lead to improvements in care quality in acute hospitals? Protocol for a realist feasibility evaluation

Authors

Corresponding author: Rebecca Randell, School of Healthcare, University of Leeds, Leeds, UK, r.randell@leeds.ac.uk

Natasha Alvarado, of Healthcare, University of Leeds, Leeds, UK, n.alvarado@leeds.ac.uk

Lynn McVey, School of Healthcare, University of Leeds, Leeds, UK, l.mcvey@leeds.ac.uk

Joanne Greenhalgh, School of Sociology and Social Policy, University of Leeds, Leeds, UK, j.greenhalgh@leeds.ac.uk

Robert West, Leeds Institute of Health Sciences, University of Leeds, Leeds, UK, r.m.west@leeds.ac.uk

Amanda Farrin, Leeds Institute of Clinical Trials Research, University of Leeds, Leeds, UK, a.j.farrin@leeds.ac.uk

Chris P. Gale, School of Medicine, University of Leeds, Leeds, UK, c.p.gale@leeds.ac.uk

Roger Parslow, School of Medicine, University of Leeds, Leeds, UK, r.c.parslow@leeds.ac.uk

Justin Keen, Leeds Institute of Health Sciences, University of Leeds, Leeds, UK, j.keen@leeds.ac.uk

Mai Elshehaly, School of Computing, University of Leeds, Leeds, UK, m.h.elshehaly@leeds.ac.uk

Roy A. Ruddle, School of Computing, University of Leeds, Leeds, UK, r.a.ruddle@leeds.ac.uk

Julia Lake, Leeds Teaching Hospitals NHS Trust, Leeds, UK, julia.lake3@nhs.net

Mamas A. Mamas, Royal Stoke Hospital, Stoke on Trent, UK, mamasmamas1@yahoo.co.uk

Richard Feltbower, School of Medicine, University of Leeds, Leeds, UK, r.g.feltbower@leeds.ac.uk

Dawn Dowding, School of Health Sciences, University of Manchester, Manchester, UK, dawn.dowding@lmanchester.ac.uk

For peer review only

For peer review only - http://bmjopen.bmj.com/site/about/guidelines.xhtml

I, the Submitting Author has the right to grant and does grant on behalf of all authors of the Work (as defined in the below author licence), an exclusive licence and/or a non-exclusive licence for contributions from authors who are: i) UK Crown employees; ii) where BMJ has agreed a CC-BY licence shall apply, and/or iii) in accordance with the terms applicable for US Federal Government officers or employees acting as part of their official duties; on a worldwide, perpetual, irrevocable, royalty-free basis to BMJ Publishing Group Ltd ("BMJ") its licensees and where the relevant Journal is co-owned by BMJ to the co-owners of the Journal, to publish the Work in BMJ Open and any other BMJ products and to exploit all rights, as set out in our licence.

The Submitting Author accepts and understands that any supply made under these terms is made by BMJ to the Submitting Author unless you are acting as an employee on behalf of your employer or a postgraduate student of an affiliated institution which is paying any applicable article publishing charge ("APC") for Open Access articles. Where the Submitting Author wishes to make the Work available on an Open Access basis (and intends to pay the relevant APC), the terms of reuse of such Open Access shall be governed by a Creative Commons licence – details of these licences and which Creative Commons licence will apply to this Work are set out in our licence referred to above.

For peer review only

ABSTRACT

Introduction: National audits are used to monitor care quality and safety and are anticipated to reduce unexplained variations in quality by stimulating quality improvement. However, variation within and between providers in the extent of engagement with national audits mean that the potential for national audit data to inform quality improvement is not being realised. This study will undertake a feasibility evaluation of QualDash, a quality dashboard designed to support clinical teams and managers to explore data from two national audits, the Myocardial Ischaemia National Audit Project (MINAP) and the Paediatric Intensive Care Audit Network (PICANet).

Methods and analysis: Realist evaluation, which involves building, testing, and refining theories of how an intervention works, provides an overall framework for this feasibility study. Realist hypotheses that describe how, in what contexts, and why QualDash is expected to provide benefit will be tested across five hospitals. A controlled interrupted time series analysis, using key MINAP and PICANet measures, will provide preliminary evidence of the impact of QualDash, while ethnographic observations and interviews over 12 months will provide initial insight into contexts and mechanisms that lead to those impacts. Feasibility outcomes include the extent to which MINAP and PICANet data are used, data completeness in the audits, and the extent to which participants perceive QualDash to be useful and express the intention to continue using it after the study period.

Ethics and dissemination: The study has been approved by University of Leeds School of Healthcare Research Ethics Committee. Study results will provide an initial understanding of how, in what contexts, and why quality dashboards lead to improvements in care quality. These will be disseminated to academic audiences, study participants, hospital IT departments, and national audits. If results show a trial is feasible, we will disseminate the QualDash software through a stepped wedge cluster randomised trial.

Trial registration: ISRCTN18289782

Keywords: Dashboard, audit and feedback, quality improvement, realist evaluation

Word count: 4,218

For peer review only - http://bmjopen.bmj.com/site/about/guidelines.xhtml

ARTICLE SUMMARY

Strengths and limitations of this study

- This study combines a controlled interrupted time series study with a qualitative multi-site case study in order to provide an initial understanding of not only whether use of a quality dashboard leads to quality improvement but also how, in what contexts, and why.
- In addition to assessing the feasibility of a trial, the study will determine the components of QualDash to be preserved in a definitive trial, appropriate outcome measures, and the contexts in which a definitive trial should be undertaken.
- The study will contribute to understanding of how realist methods can contribute to feasibility studies and the design of trials.
- Issues of data quality may be a limitation of the CITS; data completeness, and whether this changes over the course of the study, will be assessed,

INTRODUCTION

National clinical audits (NCAs), which provide comparative data on the performance of healthcare providers, are one means by which health systems around the world monitor care quality and safety. In England, a programme of over 30 NCAs is managed by the Healthcare Quality Improvement Partnership (HQIP) and all healthcare providers that contribute to delivery of the National Health Service (NHS) are required to participate. Such audits are anticipated to reduce unexplained variations in healthcare quality by stimulating quality improvement (QI) [1 2]. While there is evidence of positive impacts of NCAs [3-5], variation within and between providers in the extent to which they engage with NCAs mean the potential for NCA data to inform QI is not being realised [6 7].

Quality dashboards are a form of audit and feedback (A&F) that provide visualisations of audit data with the aim of informing QI efforts [8]. Healthcare providers are increasingly using quality dashboards. For example, quality dashboard use has been reported in Canada [9], the UK [10], and the Netherlands [11]. While quality dashboards have been shown to have positive effects on some performance indicators [9], empirical evidence regarding their impact remains limited [12].

For peer review only - http://bmjopen.bmj.com/site/about/guidelines.xhtml

QualDash

QualDash is an interactive web-based quality dashboard designed to support clinical teams and managers to explore data from two national audits, the Myocardial Ischaemia National Audit Project (MINAP) and the Paediatric Intensive Care Audit Network (PICANet), for the purpose of QI (Fig. 1). Information used to inform design of QualDash was collected through interviews with 50 clinicians and managers across five NHS Trusts (providers) and four healthcare commissioners, observations of meetings where audit data are discussed, a workshop with NCA suppliers, and two co-design workshops with clinicians and managers from one Trust.

Fig. 1: Prototype of main dashboard view for the Paediatric Intensive Care Audit Network (using simulated data).

[Figure 1 should go approximately here]

The interviews revealed that use of NCA data is largely at the clinical team level, with more limited use at divisional and corporate (Board and sub-committees that report to the Board, such as Quality and Safety Committees) levels. At all levels, a key constraint in use of NCA data for QI is lack of access to timely data; there was consensus among interviewees that data should not be more than three months old. QualDash seeks to improve access to timely data, providing users with a means to visualise the data they collect for the NCAs, without having to wait for data to be returned to them from the NCAs. There is variation between Trusts in the extent to which NCA data are used, often related to resources, which in turn impacts on timeliness of data; Trusts that make greater use of NCA data tend to have local databases from which they can generate visualisations of the data (e.g. bar charts) and audit support staff who have the time and skills to be able to generate such visualisations. In contrast, where such resources are not available, Trusts rely on the NCA annual reports, where data may be 15 months old (e.g. one annual report published in June 2017 reported data from April 2015 to March 2016). QualDash provides visualisations of key metrics, each metric being represented within a ‘QualCard’ (Fig. 2), enabling Trusts to use NCA data for QI, regardless of existing resources. QualCards for MINAP and PICANet are listed in Table 1; while there is only one set of QualCards for PICANet, for MINAP an

For peer review only

additional QualCard is provided for teaching hospitals, as discussions with sites revealed that the metrics of interest are different between teaching hospitals and District General Hospitals (DGHs). Sites are also able to create additional QualCards, to reflect local priorities.

Fig. 2: Prototype of main dashboard view for the Paediatric Intensive Care Audit Network with the Mortality QualCard expanded (using simulated data).

[Figure 2 should go approximately here]

Table 1: QualCards

	Metric
MINAP – all sites	Mortality<br>Door (arrival in Accident and Emergency) to angiogram time<br>Gold standard drugs on discharge<br>Referral for cardiac rehabilitation<br>Acute use of aspirin
MINAP – teaching hospital specific	Call (by patient/relative to emergency services) to balloon (percutaneous coronary intervention) time
PICANet – all sites	Mortality<br>48 hour unplanned readmission<br>Bed days and accidental extubation<br>Specialty case mix<br>Data quality (number of records with a missing value)<br>Patient dependency

To load new data into QualDash, NCA data are either extracted from the site's database or downloaded from the NCA website and then fed to a small script (written in R), which in turn updates the dashboard. Users can add new data as often as they want, but at a minimum they will load data into QualDash at the same time as uploading to the NCAs (typically every three months).

The benefits perceived from using QualDash may vary between sites, with under-resourced sites that previously made little use of NCA data for QI perceiving greater impact than those that already have the means to use NCA data for QI. There are also constraints on use of NCA data for QI that it may be difficult for QualDash to address. For example, in some Trusts, clinical team members perceive that relevant managers will not agree to provide the resources necessary for QI initiatives, which reduces motivation to engage with NCA data and may affect the extent to which QualDash is used. However, QualDash provides means for visualisations to be downloaded and incorporated into presentations and

For peer review only - http://bmjopen.bmj.com/site/about/guidelines.xhtml

reports, which may support clinical teams in making a stronger case for QI initiatives. Another constraint on use of NCA data for QI relates to clinicians' trust in the quality of the data. Interviews revealed variations across sites in processes for ensuring data quality. However, some interviewees also suggested that having the means to make more use of NCA data via QualDash would motivate them to improve their processes for ensuring data quality, although this will be dependent on local resources.

In this paper, we describe the methods for a realist feasibility evaluation of QualDash. The study objectives are:

1. To develop an initial understanding of how, in what contexts, and why use of QualDash leads to QI; and
2. To assess the feasibility of conducting a trial of QualDash.

As no checklists exist for reporting of realist evaluation protocols, in presenting this protocol we draw on the RAMESES II reporting standards for realist evaluations [13] (Additional file 1).

METHODS AND ANALYSIS

Study design

Use of theory is needed for design and evaluation of A&F interventions [14-16], and QI initiatives more generally [17-19]. This project draws on Realist Evaluation (RE), which involves building, testing, and refining theories about how an intervention is supposed to work [20]. These theories are expressed in the form of Context Mechanism Outcome (CMO) configurations, where C+M=O, reflecting the realist understanding that it is recipients' responses to the resources that an intervention provides (the intervention mechanisms) that determine the impact of the intervention, and such responses are highly influenced by context [21]. Consequently, RE seeks to answer not only the question of 'what works?' but 'what works for whom, in what circumstances, and why?' [22]. It is concerned with both intended and unintended outcomes. RE is recommended for studying QI [23] and has been used for studying the implementation and impact of large-scale QI programmes [24]. There is increasing interest in use of realist methods in feasibility evaluations [25-27].

We have drawn on a range of sources to develop CMO configurations which describe how, in what contexts, and why use of QualDash is anticipated to lead to QI (see Additional file 2). Data generated

For peer review only

from the interviews, observations, and workshops described above have been essential to this, as have discussions with the designers of QualDash (ME and RAR) who, drawing on their expertise in information visualisation, have their own literature-informed theories regarding why certain features of QualDash will provide benefit to users [28 29]. We have also drawn on substantive theories regarding how A&F lead to QI at the micro [30 31], meso [32], and macro level [33 34].

Data collection is designed to enable testing of the CMO configurations. Outcome data, in the form of key MINAP and PICANet measures, will be collected and analysed in a controlled interrupted time series (CITS) study, while a multi-site case study [35] will provide an initial understanding of the contexts and mechanisms that lead to those outcomes, as well as providing data on intermediate outcomes such as increased use of NCA data. A&F interventions, and QI interventions more generally, require longitudinal evaluation to allow sufficient time for staff to implement changes and incorporate them into their practice [36-38]. Similarly, evaluation of health IT (HIT) should allow time for staff to integrate the technology into their practices and evolve those practices to take advantage of the functionality offered by the technology [39]. Therefore, data will be collected over a 12 month period, from August 2019.

Public and patient involvement

A Lay Advisory Group has been established, which has contributed to the design of QualDash by reviewing the topic guide for the interviews that were conducted, providing their perspective on the findings of the interview study, and participating in the usability evaluation of QualDash. For the realist feasibility evaluation, they have provided advice on aspects to pay attention to when undertaking observations. They will contribute to analysis of a sample of the qualitative data, to provide a patient perspective. They will advise on dissemination of findings to relevant interest groups and will review outputs for comprehensibility.

Setting/context

The feasibility study will be conducted in the five NHS acute Trusts in which the interview study that informed the design of QualDash was undertaken. Three Trusts are teaching hospitals that participate in both MINAP and PICANet and have been selected to ensure variation in key outcome measures (MINAP: 30-day mortality for patients hospitalised with ST-elevation myocardial infarction; PICANet:

For peer review only - http://bmjopen.bmj.com/site/about/guidelines.xhtml

risk adjusted standardised mortality ratio). Two Trusts are DGHs that participate in MINAP but do not have a PICU and so do not participate in PICANet. These have been selected to ensure variation in the same key MINAP measure.

Multi-site case study

In the multi-site case study, data will be collected through ethnographic observation and interviews. Ethnographic methods have been argued as essential for studying implementation of QI interventions [19] and introduction of HIT [40]. Ethnography is well suited to RE because it involves observing phenomena in context, supporting understanding of how context influences the response to an intervention [41]. We will follow the Biography of Artefacts approach [42], which is concerned with capturing how particular contexts and appropriations of a technology lead to different processes and generate different outcomes, a parallel to RE's concern with contexts, mechanisms, and outcomes [43]. It involves longitudinal 'strategic ethnography' [42], where data collection is guided by a provisional understanding of the moments and locales in which a technology and associated practices evolve [43].

Data collection

In the three teaching hospitals, we will undertake a minimum of 24 periods of observation per Trust, to be split across activities related to cardiology and the PICU, and in the two DGHs we will undertake a minimum of 12 periods of observation per Trust, to be spent observing activities related to cardiology. Each period of observation will be a minimum of four hours (total n=384 hours). While researchers will return to each Trust monthly, to understand how use of QualDash changes over time, more time will be spent in the first few months following the introduction of QualDash, because this is when users are most likely to engage with and explore the affordances of QualDash and establish new practices around it, generating information with implications for system enhancement [43]. Observations will be scheduled to take place at different times of day and on different days of the week, to ensure the account of what is observed is as complete and representative as possible [44].

At each case site, an initial phase of general observation will provide an opportunity for researchers to become familiar with the setting and for those in the setting to become familiar with the presence of the researchers. Following a previous study of dashboards [10], observations will be undertaken in clinical

For peer review only - http://bmjopen.bmj.com/site/about/guidelines.xhtml

areas to understand clinical teams' working practices and capture 'corridor committees' where issues of quality and safety are discussed more informally [45]. In the PICUs, initial observations will take place on the PICU, e.g. with the researchers positioning themselves by the nurses' station, as well as observing handovers, safety huddles, and ward rounds. Because activities related to cardiology tend to be more dispersed across hospitals, researchers will first shadow clinical team members (consultant cardiologists and acute chest pain nurses) to determine where it is most appropriate to conduct subsequent observations. These initial observations will also be used to record general details of the setting that may influence QualDash use, such as staffing levels and availability of computers.

After this initial phase, observation will be guided by the CMO configurations under investigation. In addition to observing formal meetings where quality and safety are discussed, predominantly at ward level but also at divisional and corporate level, observation will involve shadowing staff members as they undertake particular activities: collection and entry of NCA data, to see if and how this changes over time; accessing and interrogating NCA data, whether using QualDash or some other means; preparation of reports and/or presentations using NCA data, again whether using QualDash or some other means. Where visualisations from QualDash are incorporated into presentations and written reports, we will follow the path of those documents, to identify staff members who may not use QualDash directly but are receiving QualDash outputs. Attention will be paid to how, in what contexts, and why QualDash and QualDash outputs are used or not, understood in the context of broader practices and use of other sources of information for monitoring care quality, and how this changes over time. We will also follow local QI initiatives, recording data on, for example, when and how the need for the QI initiative was identified, contextual factors that appear to support and constrain its introduction, how the impact of the QI initiative is monitored, and other contextual factors that appear to influence the metric that the QI initiative is targeting. Researchers will record observations in fieldnotes, which will be written up in detail as soon after data collection as possible.

Brief interviews will be undertaken opportunistically during the course of conducting observations to clarify aspects of practice that are not immediately intelligible to an observer, with participant responses recorded in fieldnotes [46]. As data collection progresses, longer semi-structured interviews will be used to discuss revisions to our CMO configurations. These will be undertaken using a particular approach

For peer review only - http://bmjopen.bmj.com/site/about/guidelines.xhtml

from RE, referred to as the teacher-learner cycle, whereby the theories under investigation are made explicit to the interviewee so that the interviewee can use their experiences to refine the researcher's understanding [47]. Being concerned with the reasoning of intervention recipients, mechanisms are often not observable [21], so these longer interviews will also provide the opportunity to explore staff reasoning about QualDash. These longer interviews will be audio recorded and transcribed verbatim.

Logfiles are widely used to evaluate visualisation tools [48]. QualDash logfiles will record information about the user (job title, etc.), data used (audit, year), overall time spent using QualDash, time spent interacting with different QualCards (including new QualCards that have been created), functionality used, and whether QualDash visualisations were downloaded. In addition to providing data regarding extent of QualDash use, how QualDash is used and by whom, and how this changes over time, information from logfiles will be used to inform qualitative data collection (e.g. asking in interviews why participants use particular QualCards and not others and the motivation behind the creation of new QualCards).

At the end of the data collection period, we will ask participants to complete a questionnaire based on the Technology Acceptance Model, using well validated items that have been used in numerous evaluations of HIT [49], including dashboards [50]. This will provide participants' perceptions of the usefulness of QualDash and data on whether they intend to continue using QualDash after the study period.

Analysis

An iterative approach to data collection and analysis will be taken, to enable: ongoing testing and refinement of the CMO configurations; gathering of further data in light of such revisions; and refinement of QualDash in response to participants' feedback. Fieldnotes and interview transcripts will be entered into NVivo 11. Narrative analysis will be undertaken to develop a 'biography' of QualDash, which will describe use of QualDash and its outputs by a range of stakeholders at different levels (clinical team, divisional, and corporate) and the interconnections between them [10]. Narrative analysis is consistent with a realist approach due to its emphasis on preserving connections within the data, thereby helping to understand causality [51]. This analysis will be supplemented with analysis of the logfiles and

For peer review only - http://bmjopen.bmj.com/site/about/guidelines.xhtml

For peer review only

questionnaire data. Findings will be compared with the CMO configurations, to determine whether they support, refute, or suggest a revision or addition to the CMO configurations.

Controlled interrupted time series study

Interrupted time series studies provide a robust method of assessing the effect of an intervention and have been used to assess effectiveness of a variety of complex interventions [52]. In a CITS, the addition of a control group enhances causal inference because the presence of seasonal trends and other potential time-varying confounders can be assessed [53]. Data will be collected across the five Trusts, with two control Trusts per intervention Trust, providing a total of 10 control Trusts. Control Trusts will be matched according to their size and outcomes pre-intervention. Having more than one control site per intervention site increases power but, as the number of control sites per intervention site increases, quality of matching decreases. Therefore, we have chosen to have two control Trusts per intervention Trust to increase power while maintaining quality of the matching.

Given the study intention to determine the feasibility of and inform the design of a trial, a range of measures will be considered. Initially, we selected two process measures, one for MINAP and one for PICANet. For MINAP, we selected the composite process measure Cumulative Missed Opportunities for Care (CMOC). This has nine components (pre-hospital ECG, acute use of aspirin, timely perfusion, referral for cardiac rehabilitation, and prescription at hospital discharge of what are considered to be the gold standard drugs – aspirin, thienopyridine inhibitor, ACE-inhibitor, HMG-CoA reductase inhibitor, and beta blockers) and is inversely associated with mortality [54]. As some of these components, such as pre-hospital ECG, are outside the direct control of the Trust, we will also explore the impact of QualDash on the individual measures that make up CMOC. On the basis of the measures that cardiology clinicians described in the interviews as being important for measuring care quality, we will also look at the percentage of patients who receive an angiogram within 72 hours from first admission to hospital, which is part of the Best Practice Tariff financial incentive scheme, and, for those hospitals that provide percutaneous coronary intervention (PCI), the proportion of patients who have a door-to-balloon time (the time from arrival at the hospital to PCI) of less than 60 minutes. Our CMO configurations (Additional file 2) suggest improvement will be seen in measures if: clinical teams perceive them as being important indicators of care and/or they relate to financial incentives;

For peer review only - http://bmjopen.bmj.com/site/about/guidelines.xhtml

performance is not in line with expectations; they perceive the measure as being within their control; and the team is resourced to introduce QI initiatives in relation to these measures.

For PICANet, we selected use of non-invasive ventilation first for patients requiring ventilation, which has been shown to be associated with reduced mortality [55]. However, this was not raised as an area of concern in our interviews with PICU clinicians. On the basis of this and two additional considerations – it would require loading additional data into QualDash which would reduce the performance of QualDash in terms of speed and it requires computation of the data, while the focus of QualDash is on visualising the data – a QualCard has not been created for this metric. Therefore, while we will still include this measure in the CITS, we do not hypothesise that it will change, unless other sources of information, such as the PICANet annual report, draw a PICU team's attention to it. However, accidental extubation and unplanned readmission within 48 hours were identified in our interviews with PICU clinicians as being important indicators of care quality, so we will include these two measures in the CITS. On the basis of our CMO configurations (Additional file 2), we would expect to see an improvement in these measures in sites where performance is not in line with expectations, if the team is resourced to introduce QI initiatives in relation to these measures.

Sample size considerations

A CITS study requires data for a minimum of three time points pre-intervention and three time points post-intervention and must also allow for any seasonal effect on the outcomes [56]. Monthly data will be obtained for 24 months pre-intervention and 12 months post-intervention. Consequently, for each intervention Trust, there will be 72 data points prior to introduction (24 for the intervention Trust and 48 for the control Trusts) and 36 data points post intervention (12 for the intervention Trust and 24 for the control Trusts). Sample size calculations were undertaken based on our two initial measures, CMOC for MINAP and use of non-invasive ventilation first for patients requiring ventilation for PICANet; full details are provided in Additional file 3.

Analysis

Monthly MINAP and PICANet data will be extracted to spreadsheets for analysis with R software [57]. For both NCAs, each outcome will be regressed upon time and the intervention. The time component

For peer review only - http://bmjopen.bmj.com/site/about/guidelines.xhtml

will include a seasonal effect (quarterly effect) and will allow for a (linear) time trend. To account for clustering of monthly observations within hospitals, a random intercept will be fitted, although a fixed effect for hospital as a sensitivity analysis will be explored. Although the intervention is abrupt, its impact may well be 'phased in' over a few months, perhaps three. The timing of the bedding in of the intervention will be reported from the multi-site case study. Then a partial effect can be considered for this period with the interaction effect stepping up in a linear fashion.

Results of the CITS analysis will be incorporated into the biography of QualDash, the analysis of the data from the multi-site case study describing how contextual factors shape the evolution of practices around QualDash and how this leads to the resulting outcome pattern.

Trial feasibility assessment and design

Our trial progression criteria are: (i) the number of people who engage with either MINAP or PICANet data (via QualDash or some other means) is the same or higher than the number of people who engaged with either MINAP or PICANet data prior to QualDash's introduction; (ii) data completeness in the national audit improves or remains the same; (iii) 50% or more of participants in the questionnaire survey perceive QualDash to be useful and express the intention to continue using it after the study period. Criteria (i) and (ii) are concerned with ensuring the intervention does not have unintended negative consequences which would affect success of the intervention. Criterion (ii) is also concerned with feasibility of outcome assessment. Criterion (iii) is concerned with acceptability and uptake of the intervention, and therefore has implications for recruitment to a trial, as well as being concerned with participants' perceptions of the impact of QualDash on care. While not formally assessed as part of the progression criteria, the impact of QualDash on care as identified in the CITS will be considered in determining whether a future trial is justified. A traffic light system will be used to determine if a trial is feasible (green), feasible with modifications to QualDash (amber), or not feasible (red) [58 59].

If the results show a trial of QualDash is feasible, we will design a stepped wedge cluster randomised trial. Data from the CITS will be used to inform the selection of NCAs to be included in the trial (MINAP and/or PICANet) and will provide information about variability of outcomes and about how long a trial intervention period would need to be. Findings from the multi-site case study will be used to inform the

For peer review only - http://bmjopen.bmj.com/site/about/guidelines.xhtml

selection of categories of user to be included in the trial and, associated with this, the level of randomisation (Trust, hospital, or ward). Using the understanding of the relationship between contexts, mechanisms, and outcomes provided by the study, we will identify QualDash components associated with mechanisms that produce the desired outcomes in order for them to be preserved in the trial, while other components can be adapted to suit the local context.

ETHICS AND DISSEMINATION

Ethics approval has been received from the University of Leeds School of Healthcare Research Ethics Committee (Approval no.HREC16-044). Written consent will be obtained from participants for interviews and for meeting observations.

Study results will provide initial understanding of how and in what contexts quality dashboards may lead to improvements in care quality. We will disseminate these results to academic audiences, study participants, hospital IT departments, and NCAs. If we progress to a trial, in addition to providing further understanding of the impact of quality dashboards on care quality, this will result in wider dissemination of the QualDash software.

Acknowledgements

This research is funded by the National Institute for Health Research (NIHR) Health Services and Delivery Research (HS&DR) Programme (project number 16/04/06). The views and opinions expressed are those of the presenter and do not necessarily reflect those of the HS&DR programme, NIHR, NHS or the Department of Health.

Data statement

The qualitative data gathered during the course of this evaluation will be kept until June 2030 and can be accessed by other researchers during this time, subject to the necessary ethical approvals being obtained. Requests for access to this data should be addressed to the corresponding author.

For peer review only

For peer review only - http://bmjopen.bmj.com/site/about/guidelines.xhtml

Authors' contributions

RR is Principal Investigator for the study. She conceived, designed, and secured funding for the study in collaboration with JG, RW, AF, CPG, RP, JK, RAR, JL, MM, and DD. NA and LM led the qualitative data collection and analysis that informed the design of QualDash and the design of evaluation. ME developed the QualDash software and contributed to the design of the evaluation. RP and RF provided data for the testing of QualDash and provided significant feedback on its design. All authors provided input into various aspects of the evaluation design and revised drafts of the protocol. RR led the writing of this protocol manuscript. All authors read and approved the final manuscript.

Funding statement

This research is funded by the National Institute for Health Research (NIHR) Health Services and Delivery Research (HS&DR) Programme (project number 16/04/06).

Competing interests statement

Chris Gale is a member of the MINAP Academic and Steering Groups. Richard Feltbower is Principal Investigator for PICANet and Roger Parslow was previously Principal Investigator for PICANet. The authors have no other competing interests to declare.

Additional files

Additional file 1: Checklist of RAMESES II reporting standards for realist evaluations (PDF)

Additional file 2: Context Mechanism Outcome configurations to be tested in the realist feasibility evaluation (PDF)

Additional file 3: Sample size calculations for controlled interrupted time series (PDF)

REFERENCES

1. Phekoo KJ, Clements J, Bell D. National Clinical Audit Quality Assessment - Overview of the self-assessment survey: "audit of audits". London: Healthcare Quality Improvement Partnership, 2014.
2. Miller T, Leatherman S. The National Quality Forum: a'me-too'or a breakthrough in quality measurement and reporting? Health Aff. (Millwood) 1999;**18**(6):233-37.
3. Birkhead JS, Walker L, Pearson M, Weston C, Cunningham AD, Rickards AF. Improving care for patients with acute coronary syndromes: initial results from the National Audit of Myocardial Infarction Project (MINAP). Heart 2004;**90**(9):1004-09.

For peer review only - http://bmjopen.bmj.com/site/about/guidelines.xhtml

4. Bridgewater B, Grayson AD, Brooks N, et al. Has the publication of cardiac surgery outcome data been associated with changes in practice in northwest England: an analysis of 25 730 patients undergoing CABG surgery under 30 surgeons over eight years. Heart 2007;**93**(6):744-48.

5. Neuburger J, Currie C, Wakeman R, et al. The Impact of a National Clinician-led Audit Initiative on Care and Mortality after Hip Fracture in England: An External Evaluation using Time Trends in Non-audit Data. Med. Care 2015;**53**(8):686-91.

6. Allwood D. Engaging Clinicians in Quality Improvement through National Clinical Audit. London: Healthcare Quality Improvement Partnership, 2014.

7. Taylor A, Neuburger J, Walker K, Cromwell D, Groene O. How is feedback from national clinical audits used? Views from English National Health Service trust audit leads. J. Health Serv. Res. Policy 2016;**21**(2):91-100.

8. Pauwels K, Ambler T, Clark BH, et al. Dashboards as a Service Why, What, How, and What Research Is Needed? Journal of Service Research 2009;**12**(2):175-89.

9. Weiss D, Dunn SI, Sprague AE, et al. Effect of a population-level performance dashboard intervention on maternal-newborn outcomes: an interrupted time series study. BMJ Quality & Safety 2018;**27**(6):425-36.

10. Keen J, Nicklin E, Long A, et al. Quality and safety between ward and board: a biography of artefacts study. Health Services and Delivery Research 2018;**6**(22).

11. Weggelaar-Jansen AMJWM, Broekharst DSE, de Bruijne M. Developing a hospital-wide quality and safety dashboard: a qualitative research study. BMJ Quality & Safety 2018.

12. Dowding D, Randell R, Gardner P, et al. Dashboards for improving patient care: Review of the literature. International Journal of Medical Informatics 2015;**84**(2):87-100.

13. Wong G, Westhorp G, Manzano A, Greenhalgh J, Jagosh J, Greenhalgh T. RAMESES II reporting standards for realist evaluations. BMC Med. 2016;**14**(1):96.

14. Foy R, Eccles MP, Jamtvedt G, Young J, Grimshaw JM, Baker R. What do we know about how to do audit and feedback? Pitfalls in applying evidence from a systematic review. BMC Health Serv. Res. 2005;**5**:50.

15. Ivers NM, Grimshaw JM, Jamtvedt G, et al. Growing Literature, Stagnant Science? Systematic Review, Meta-Regression and Cumulative Analysis of Audit and Feedback Interventions in Health Care. J. Gen. Intern. Med. 2014;**29**(11):1534-41.

16. Ivers NM, Sales A, Colquhoun H, et al. No more 'business as usual' with audit and feedback interventions: towards an agenda for a reinvigorated intervention. Implementation Science 2014;**9**(1):1-8.

17. Grol RPTM, Bosch MC, Hulscher MEJL, Eccles MP, Wensing M. Planning and Studying Improvement in Patient Care: The Use of Theoretical Perspectives. Milbank Q. 2007;**85**(1):93-138.

18. Davidoff F, Dixon-Woods M, Leviton L, Michie S. Demystifying theory and its use in improvement. BMJ Quality & Safety 2015.

19. Dixon-Woods M, Bosk CL, Aveling EL, Goeschel CA, Pronovost PJ. Explaining Michigan: Developing an Ex Post Theory of a Quality Improvement Program. Milbank Q. 2011;**89**(2):167-205.

20. Pawson R, Tilley N. *Realistic Evaluation*. London: SAGE Publications, 1997.

21. Dalkin S, Greenhalgh J, Jones D, Cunningham B, Lhussier M. What's in a mechanism? Development of a key concept in realist evaluation. Implementation Science 2015;**10**(1):49.

22. Pawson R. *The science of evaluation: a realist manifesto*. London: SAGE, 2013.

23. Robert G, Fulop N. The role of context in successful improvement. Perspectives on context. A selection of essays considering the role of context in successful quality improvement. London: Health Foundation 2014;**31**.

24. Greenhalgh T, Humphrey C, Hughes J, Macfarlane F, Butler C, Pawson R. How Do You Modernize a Health Service? A Realist Evaluation of Whole-Scale Transformation in London. Milbank Q. 2009;**87**(2):391-416.

For peer review only

For peer review only - http://bmjopen.bmj.com/site/about/guidelines.xhtml

25. Fletcher A, Jamal F, Moore G, Evans RE, Murphy S, Bonell C. Realist complex intervention science: Applying realist principles across all phases of the Medical Research Council framework for developing and evaluating complex interventions. Evaluation 2016;**22**(3):286-303.
26. Brand SL, Quinn C, Pearson M, et al. Building programme theory to develop more adaptable and scalable complex interventions: Realist formative process evaluation prior to full trial. Evaluation 2019;**25**(2):149-70.
27. Randell R, Honey S, Hindmarsh J, et al. A realist process evaluation of robot-assisted surgery: integration into routine practice and impacts on communication, collaboration and decision-making. Health Services and Delivery Research 2017;**5**(20).
28. Andrienko N, Andrienko G. *Exploratory analysis of spatial and temporal data: a systematic approach*: Springer Science & Business Media, 2006.
29. Brehmer M, Munzner T. A multi-level typology of abstract visualization tasks. IEEE transactions on visualization and computer graphics 2013;**19**(12):2376-85.
30. Hysong SJ, Best RG, Pugh JA. Audit and feedback and clinical practice guideline adherence: Making feedback actionable. Implementation Science 2006;**1**(1):1-10.
31. Sapyta J, Riemer M, Bickman L. Feedback to clinicians: Theory, research, and practice. J. Clin. Psychol. 2005;**61**(2):145-53.
32. Van Helden GJ, Tillema S. In Search of a Benchmarking Theory for the Public Sector. Financial Accountability & Management 2005;**21**(3):337-61.
33. Scott RW. *Institutions and Organizations*. 2nd Edition ed. London: Sage, 2001.
34. Furusten S. *Institutional Theory and Organizational Change*. Cheltenham, UK: Edward Elgar, 2013.
35. Yin RK. *Case study research: design and methods*. 3rd ed. Thousand Oaks, California: SAGE, 2003.
36. Øvretveit J, Gustafson D. Evaluation of quality improvement programmes. Quality and Safety in Health Care 2002;**11**(3):270-75.
37. Ramsay AIG, Turner S, Cavell G, et al. Governing patient safety: lessons learned from a mixed methods evaluation of implementing a ward-level medication safety scorecard in two English NHS hospitals. BMJ Quality & Safety 2013;**23**:136-46.
38. Benn J, Burnett S, Parand A, Pinto A, Iskander S, Vincent C. Studying large-scale programmes to improve patient safety in whole care systems: Challenges for research. Soc. Sci. Med. 2009;**69**(12):1767-76.
39. Hyysalo S. *Health technology development and use: from practice-bound imagination to evolving impacts*. New York: Routledge, 2010.
40. Greenhalgh T, Swinglehurst D. Studying technology use as social practice: the untapped potential of ethnography. BMC Med. 2011;**9**(1):45.
41. Dainty KN, Golden BR, Hannam R, et al. A realist evaluation of value-based care delivery in home care: The influence of actors, autonomy and accountability. Soc. Sci. Med. 2018;**206**:100-09.
42. Pollock N, Williams R. *Software and organisations: The biography of the enterprise-wide system or how SAP conquered the world*. New York: Routledge, 2008.
43. Pollock N, Williams R. e-Infrastructures: How Do We Know and Understand Them? Strategic Ethnography and the Biography of Artefacts. Computer Supported Cooperative Work (CSCW) 2010;**19**(6):521-56.
44. Hammersley M, Atkinson P. *Ethnography: principles in practice*. London: Routledge, 1995.
45. Waring J. Adaptive regulation or governmentality: patient safety and the changing regulation of medicine. Sociol. Health Illn. 2007;**29**(2):163-79.
46. Goodwin D, Pope C, Mort M, Smith A. Access, boundaries and their effects: legitimate participation in anaesthesia. Sociol. Health Illn. 2005;**27**(6):855-71.
47. Pawson R. Theorizing the Interview. The British Journal of Sociology 1996;**47**(2):295-314.
48. Harrison DG, Efford ND, Fisher QJ, Ruddle RA. PETMiner—A Visual Analysis Tool for Petrophysical Properties of Core Sample Data. IEEE transactions on visualization and computer graphics 2017;**24**(5):1728-41.

For peer review only - http://bmjopen.bmj.com/site/about/guidelines.xhtml

49. Holden RJ, Karsh B-T. The Technology Acceptance Model: Its past and its future in health care. Journal of Biomedical Informatics 2010;**43**(1):159-72.
50. Lee K, Jung SY, Hwang H, et al. A novel concept for integrating and delivering health information using a comprehensive digital dashboard: An analysis of healthcare professionals' intention to adopt a new system and the trend of its real usage. Int. J. Med. Inform. 2017;**97**:98-108.
51. Maxwell JA. *A realist approach for qualitative research*. London: SAGE Publications, 2012.
52. Hu Y. Stepped wedge, natural experiments and interrupted time series analysis designs. In: Richards DA, Hallberg IR, eds. Complex interventions in health: An overview of research methods. Abingdon: Routledge, 2015:200-12.
53. Handley MA, Lyles CR, McCulloch C, Cattamanchi A. Selecting and Improving Quasi-Experimental Designs in Effectiveness and Implementation Research. Annu. Rev. Public Health 2018;**39**(1):5-25.
54. Simms AD, Baxter PD, Cattle BA, et al. An assessment of composite measures of hospital performance and associated mortality for patients with acute myocardial infarction. Analysis of individual hospital performance and outcome for the National Institute for Cardiovascular Outcomes Research (NICOR). European Heart Journal: Acute Cardiovascular Care 2013;**2**(1):9-18.
55. Morris JV, Ramnarayan P, Parslow RC, Fleming SJ. Outcomes for Children Receiving Noninvasive Ventilation as the First-Line Mode of Mechanical Ventilation at Intensive Care Admission: A Propensity Score-Matched Cohort Study. Crit. Care Med. 2017.
56. Cochrane Effective Practice and Organisation of Care Group (EPOC). What study designs should be inlcuded in an EPOC review and what should they be called? Oslo: Norwegian Knowledge Centre for the Health Services, 2013.
57. R Core Team. R: A language and environment for statistical computing. Vienna, Austria: R Foundation for Statistical Computing, 2014.
58. Bugge C, Williams B, Hagen S, et al. A process for Decision-making after Pilot and feasibility Trials (ADePT): development following a feasibility study of a complex intervention for pelvic organ prolapse. Trials 2013;**14**(1):353.
59. Thabane L, Ma J, Chu R, et al. A tutorial on pilot studies: the what, why and how. BMC Med. Res. Methodol. 2010;**10**(1):1-10.

For peer review only

For peer review only - http://bmjopen.bmj.com/site/about/guidelines.xhtml

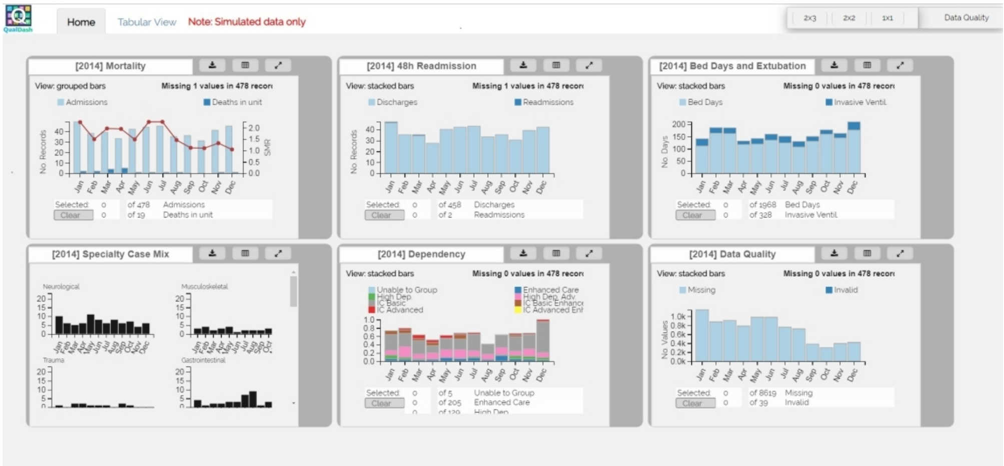

Fig. 1: Prototype of main dashboard view for the Paediatric Intensive Care Audit Network (using simulated data).

161x74mm (300 x 300 DPI)

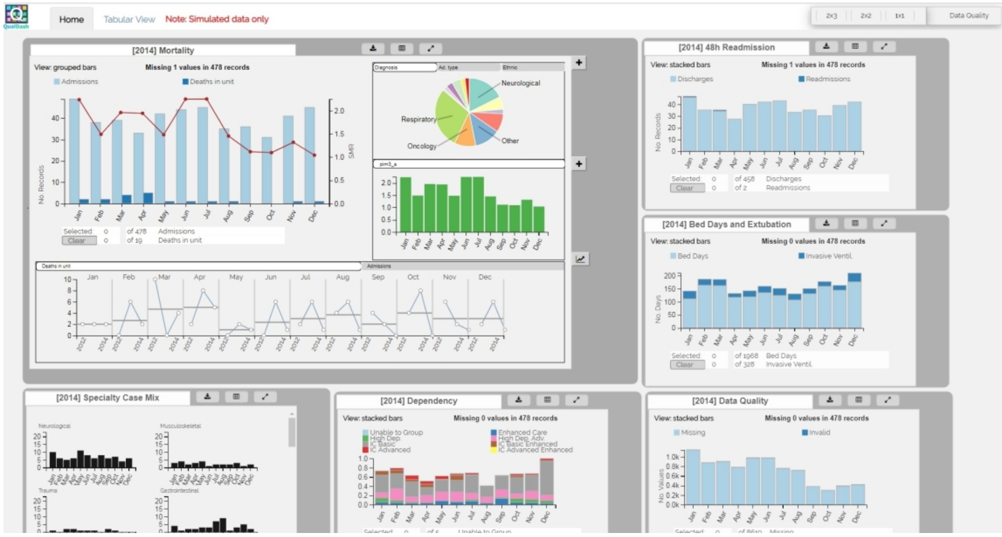

Fig. 2: Prototype of main dashboard view for the Paediatric Intensive Care Audit Network with the Mortality QualCard expanded (using simulated data).

161x85mm (300 x 300 DPI)

RAMESES II reporting standards for realist evaluations

How, in what contexts, and why do quality dashboards lead to improvements in care quality in acute hospitals? Protocol for a realist feasibility evaluation			Reported in document Y/N/NA	Page(s) in document	Comment
1		In the title, identify the document as a realist evaluation	Y	1	
SUMMARY OR ABSTRACT					
2		Journal articles will usually require an abstract, while reports and other forms of publication will usually benefit from a short summary. The abstract or summary should include brief details on: the policy, programme or initiative under evaluation; programme setting; purpose of the evaluation; evaluation question(s) and/or objective(s); evaluation strategy; data collection, documentation and analysis methods; key findings and conclusions<br><br>Where journals require it and the nature of the study is appropriate, brief details of respondents to the	Y	3	

For peer review only

		evaluation and recruitment and sampling processes may also be included<br><br>Sufficient detail should be provided to identify that a realist approach was used and that realist programme theory was developed and/or refined			
INTRODUCTION					
3	Rationale for evaluation	Explain the purpose of the evaluation and the implications for its focus and design	Y	7	
4	Programme theory	Describe the initial programme theory (or theories) that underpin the programme, policy or initiative	Y	8 and Additional file 2	Placed in body of article, rather than Introduction, as more appropriate for protocol
5	Evaluation questions, objectives and focus	State the evaluation question(s) and specify the objectives for the evaluation. Describe whether and how the programme theory was used to define the scope and focus of the evaluation	Y	7	
6	Ethical approval	State whether the realist evaluation required and has gained ethical approval from the relevant	Y	15	

For peer review only

		authorities, providing details as appropriate. If ethical approval was deemed unnecessary, explain why			
METHODS					
7	Rationale for using realist evaluation	Explain why a realist evaluation approach was chosen and (if relevant) adapted	Y	7-8	
8	Environment surrounding the evaluation	Describe the environment in which the evaluation took place	Y	8-9	
9	Describe the programme policy, initiative or product evaluated	Provide relevant details on the programme, policy or initiative evaluated	Y	5-7	Description of intervention placed in Introduction as this seemed more appropriate in providing the context for the protocol
10	Describe and justify the evaluation design	A description and justification of the evaluation design (i.e. the account of what was planned, done and why) should be included, at least in summary form or as an appendix, in the document which presents the main findings. If this is not done, the omission should be justified and a reference or link to the evaluation	Y	7-14	

For peer review only

		design given. It may also be useful to publish or make freely available (e.g. online on a website) any original evaluation design document or protocol, where they exist			
11	Data collection methods	Describe and justify the data collection methods – which ones were used, why and how they fed into developing, supporting, refuting or refining programme theory<br><br>Provide details of the steps taken to enhance the trustworthiness of data collection and documentation	Y	9-11	
12	Recruitment process and sampling strategy	Describe how respondents to the evaluation were recruited or engaged and how the sample contributed to the development, support, refutation or refinement of programme theory	Y	8-9	Sampling of sites, rather than individuals, is described; Recruitment will be described when reporting the results of the study
13	Data analysis	Describe in detail how data were analysed. This section should include information on the constructs that were identified, the process of	Y	11-12, 13-14	

For peer review only

		analysis, how the programme theory was further developed, supported, refuted and refined, and (where relevant) how analysis changed as the evaluation unfolded			
RESULTS					
14	Details of participants	Report (if applicable) who took part in the evaluation, the details of the data they provided and how the data was used to develop, support, refute or refine programme theory	NA		Protocol so no results to report
15	Main findings	Present the key findings, linking them to contexts, mechanisms and outcome configurations. Show how they were used to further develop, test or refine the programme theory	NA		Protocol so no results to report
DISCUSSION					
16	Summary of findings	Summarise the main findings with attention to the evaluation questions, purpose of the evaluation, programme theory and intended audience	NA		Protocol so no results to report
17	Strengths, limitations and future directions	Discuss both the strengths of the evaluation and its limitations. These should include (but need not be	4		Strengths and limitations of study design

For peer review only

		limited to): (1) consideration of all the steps in the evaluation processes; and (2) comment on the adequacy, trustworthiness and value of the explanatory insights which emerged<br><br>In many evaluations, there will be an expectation to provide guidance on future directions for the programme, policy or initiative, its implementation and/or design. The particular implications arising from the realist nature of the findings should be reflected in these discussions			
18	Comparison with existing literature	Where appropriate, compare and contrast the evaluation's findings with the existing literature on similar programmes, policies or initiatives	NA		Protocol so no results to compare with existing literature
19	Conclusion and recommendations	List the main conclusions that are justified by the analyses of the data. If appropriate, offer recommendations consistent with a realist approach	NA		Protocol so no results on which to base recommendations
20	Funding and conflict of interest	State the funding source (if any) for the evaluation, the role played by the	Y	16	

For peer review only

		funder (if any) and any conflicts of interests of the evaluators			

For peer review only

How, in what contexts, and why do quality dashboards lead to improvements in care quality in acute hospitals? Protocol for a realist feasibility evaluation (Randell et al.)

Additional file 2

#	Context	+	Mechanism		=	Outcome
			Resource	Response		
**1.**	Teams previously constrained in their ability to use NCA data for monitoring service performance because data not considered to be timely, accurate, and/or complete	+	QualDash offers easy access to key metrics	Teams are able to see whether the data displayed are timely, accurate, and/or complete and, where they are not, adjust their data collection processes in order to benefit from QualDash	=	Improvement in data quality in terms of timeliness, accuracy, and completeness – as data quality improves, use of QualDash increases
				Teams use QualDash to embed NCA data within their monitoring processes e.g. in clinical governance meetings where data is presented visually via screens.	=	Increased routine use of NCA data in performance monitoring, providing opportunities for its use in quality improvement
**2.**	Teams previously using NCA data to monitor service performance routinely by extracting raw data and producing reports for review in meetings and by individuals	+	QualDash visualises key metrics in ways that clearly show whether service performance is within an expected range and provides functions to interrogate that data	Teams use QualDash to facilitate their existing processes for monitoring service performance using NCA data	=	Reduced time spent in accessing, and preparing visualisations of, NCA data

For peer review only - http://bmjopen.bmj.com/site/about/guidelines.xhtml

#	Context	+	Mechanism		=	Outcome
			**Resource**	**Response**		
**3.**	Teams who want to use NCA data but were previously constrained by data quality and existing systems did not provide functions to easily access and interact with the data	**+**	QualDash provides functions that enable users to interact with NCA data and explore relationships between variables	Teams will use these functions to interrogate anomalies in the data, which will help them to understand what has impacted performance, thereby enabling them to identify appropriate strategies for improving performance	**=**	Introduction of QI initiatives in relation to metrics that teams consider important and where performance is not in line with expectations<br><br>Over time, improvement in metrics that QI initiatives target
**4.**	Performance in key metrics, such as the Best Practice Tariff, is in line with expectations<br><br>Relevant audit/IT support staff have time and willingness to support use of QualDash	**+**	QualDash offers teams the ability to quickly and easily add new QualCards (within NCA parameters)	Teams add new QualCards to be able to monitor and interrogate metrics they have chosen as important	**=**	Introduction of QI initiatives in relation to metrics shown on new QualCards when performance is not in line with expectations<br><br>Over time, improvement in metrics that QI initiatives target

For peer review only

#	Context	+	Mechanism		=	Outcome
			Resource	Response		
**5.**	Teams who previously did not, or were not able to, monitor key metrics routinely<br><br>Performance is not in line with expectations in key metrics<br><br>Teams are resourced to make practice changes	**+**	QualDash provides quick and easy access to key metrics	Teams will become aware of discrepancies between performance and targets in key metrics, which they will take action to address	**=**	Introduction of QI initiatives in relation to key metrics<br><br>Over time, improvement in those metrics
**6.**	Teams are asked to produce reports and recommendations for managers and other groups about service performance, e.g. at the time of publication of NCA annual report	**+**	QualDash offers easy access to NCA data and visualisations that can be exported into reports	Teams will use QualDash to produce performance reports requested by other groups	**=**	Reduced time spent in report preparation<br><br>Increased use of NCA data at divisional and corporate levels via outputs produced by QualDash<br><br>Over time, use of QualDash at divisional and/or corporate levels, due to increased awareness of NCA data

For peer review only - http://bmjopen.bmj.com/site/about/guidelines.xhtml

For peer review only

#	Context	+	Mechanism: Resource	Mechanism: Response	=	Outcome
**7.**	Teams receive data requests from service managers	**+**	QualDash can be easily accessed via the web by multiple users	Service managers will use QualDash to access the information they need quickly and easily	**=**	Streamlines the use of NCA data for clinical managers<br><br>Reduced time spent by audit support staff/clinical team in producing data reports for managers
**8.**	Teams need to evidence their performance to managers and other groups in order to support a case for practice change e.g. in business meetings with managers or in the NCA annual report summary	**+**	QualDash visualises performance metrics, which can also be exported into reports and presentations	Teams will use these functions to evidence service performance, in order to convince other Trust groups that change is needed	**=**	Other Trust groups, who are able to offer additional resource to teams, are convinced of the need for change based on the evidence provided. However, this is likely to be where those outputs are clearly associated with Trust priorities, e.g. relating to Trust reputation or avoiding penalties/gaining incentives.

For peer review only

How, in what contexts, and why do quality dashboards lead to improvements in care quality in acute hospitals? Protocol for a realist feasibility evaluation (Randell et al.)

Additional file 3

The average CMOC for patients on a ward will be averaged for each month, so that there are 36 observations clustered within each of 15 hospitals. Taking the intra-class correlation to be 0.15, this yields a design effect of 6.25. Hence the effective number of observations is 15*36/6.25=86.4. Using Cohen's approach to sample size calculation means an effect size of 0.17 can be estimated with 80% given that there are six parameters in the model (including the coefficient for QualDash). Converting this to the percentage of variation that can be explained by the model, this yields 20.5%. Translating this back to CMOC, currently 49.6% of patients are discharged from hospital without missing any of the nine opportunities for care, and we would be powered at the 80% level to detect an improvement from an average of 8.33 opportunities achieved to 8.46. Thus our study has good power to detect small but meaningful clinical improvements. For PICANet, 10% of the admitted population receive non-invasive ventilation first [1]. On average there are 5.25 ventilation cases per month per hospital. With a further design effect from patients clustered within hospitals, based on the reported intraclass correlation coefficient of 0.065 giving a design effect of 1.276, the actual anticipated number of patients is 1701 giving an effective number of 213: 71 exposed to QualDash and 142 controls. This yields 80% power to detect a change from 32% to 53%.

References

1. Morris JV, Ramnarayan P, Parslow RC, Fleming SJ. Outcomes for Children Receiving Noninvasive Ventilation as the First-Line Mode of Mechanical Ventilation at Intensive Care Admission: A Propensity Score-Matched Cohort Study. Crit. Care Med. 2017.

For peer review only

BMJ Open

How, in what contexts, and why do quality dashboards lead to improvements in care quality in acute hospitals? Protocol for a realist feasibility evaluation

Journal:	*BMJ Open*
Manuscript ID	bmjopen-2019-033208.R3
Article Type:	Protocol
Date Submitted by the Author:	20-Jan-2020
Complete List of Authors:	Randell, Rebecca; University of Bradford, Faculty of Health Studies<br>Alvarado, Natasha; University of Leeds, School of Healthcare<br>McVey, Lynn; University of Leeds, School of Healthcare<br>Greenhalgh, Joanne; University of Leeds, Sociology and Social Policy<br>West, Robert; University of Leeds, Leeds Institute of Health Sciences<br>Farrin, Amanda; University of Leeds, Clinical Trials Research Unit<br>Gale, Chris; University of Leeds<br>Parslow, Roger; University of Leeds, Leeds Institute if Genetics, Health and Therapeutics<br>Keen, Justin; University of Leeds, Leeds Institute of Health Sciences<br>Elshehaly, Mai; University of Leeds, School of Computing<br>Ruddle, Roy; University of Leeds, School of Computing<br>Lake, Julia; Leeds Teaching Hospitals NHS Trust<br>Mamas, Mamas; Royal Stoke University Hospital<br>Feltbower, Richard; University of Leeds,<br>Dowding, Dawn; University of Manchester
**Primary Subject Heading**:	Health informatics
Secondary Subject Heading:	Health services research
Keywords:	AUDIT, Health informatics < BIOTECHNOLOGY & BIOINFORMATICS, Clinical audit < HEALTH SERVICES ADMINISTRATION & MANAGEMENT, Quality in health care < HEALTH SERVICES ADMINISTRATION & MANAGEMENT, QUALITATIVE RESEARCH

SCHOLARONE™
Manuscripts

I, the Submitting Author has the right to grant and does grant on behalf of all authors of the Work (as defined in the below author licence), an exclusive licence and/or a non-exclusive licence for contributions from authors who are: i) UK Crown employees; ii) where BMJ has agreed a CC-BY licence shall apply, and/or iii) in accordance with the terms applicable for US Federal Government officers or employees acting as part of their official duties; on a worldwide, perpetual, irrevocable, royalty-free basis to BMJ Publishing Group Ltd ("BMJ") its licensees and where the relevant Journal is co-owned by BMJ to the co-owners of the Journal, to publish the Work in this journal and any other BMJ products and to exploit all rights, as set out in our licence.

The Submitting Author accepts and understands that any supply made under these terms is made by BMJ to the Submitting Author unless you are acting as an employee on behalf of your employer or a postgraduate student of an affiliated institution which is paying any applicable article publishing charge ("APC") for Open Access articles. Where the Submitting Author wishes to make the Work available on an Open Access basis (and intends to pay the relevant APC), the terms of reuse of such Open Access shall be governed by a Creative Commons licence – details of these licences and which Creative Commons licence will apply to this Work are set out in our licence referred to above.

Other than as permitted in any relevant BMJ Author's Self Archiving Policies, I confirm this Work has not been accepted for publication elsewhere, is not being considered for publication elsewhere and does not duplicate material already published. I confirm all authors consent to publication of this Work and authorise the granting of this licence.

For peer review only

Title: How, in what contexts, and why do quality dashboards lead to improvements in care quality in acute hospitals? Protocol for a realist feasibility evaluation

Authors

Corresponding author: Rebecca Randell, Faculty of Health Studies, University of Bradford, Bradford, UK, r.randell@bradford.ac.uk

Natasha Alvarado, of Healthcare, University of Leeds, Leeds, UK, n.alvarado@leeds.ac.uk

Lynn McVey, School of Healthcare, University of Leeds, Leeds, UK, l.mcvey@leeds.ac.uk

Joanne Greenhalgh, School of Sociology and Social Policy, University of Leeds, Leeds, UK, j.greenhalgh@leeds.ac.uk

Robert West, Leeds Institute of Health Sciences, University of Leeds, Leeds, UK, r.m.west@leeds.ac.uk

Amanda Farrin, Leeds Institute of Clinical Trials Research, University of Leeds, Leeds, UK, a.j.farrin@leeds.ac.uk

Chris P. Gale, School of Medicine, University of Leeds, Leeds, UK, c.p.gale@leeds.ac.uk

Roger Parslow, School of Medicine, University of Leeds, Leeds, UK, r.c.parslow@leeds.ac.uk

Justin Keen, Leeds Institute of Health Sciences, University of Leeds, Leeds, UK, j.keen@leeds.ac.uk

Mai Elshehaly, School of Computing, University of Leeds, Leeds, UK, m.h.elshehaly@leeds.ac.uk

Roy A. Ruddle, School of Computing, University of Leeds, Leeds, UK, r.a.ruddle@leeds.ac.uk

Julia Lake, Leeds Teaching Hospitals NHS Trust, Leeds, UK, julia.lake3@nhs.net

Mamas A. Mamas, Royal Stoke Hospital, Stoke on Trent, UK, mamasmamas1@yahoo.co.uk

Richard Feltbower, School of Medicine, University of Leeds, Leeds, UK, r.g.feltbower@leeds.ac.uk

Dawn Dowding, School of Health Sciences, University of Manchester, Manchester, UK, dawn.dowding@lmanchester.ac.uk

For peer review only

For peer review only - http://bmjopen.bmj.com/site/about/guidelines.xhtml

I, the Submitting Author has the right to grant and does grant on behalf of all authors of the Work (as defined in the below author licence), an exclusive licence and/or a non-exclusive licence for contributions from authors who are: i) UK Crown employees; ii) where BMJ has agreed a CC-BY licence shall apply, and/or iii) in accordance with the terms applicable for US Federal Government officers or employees acting as part of their official duties; on a worldwide, perpetual, irrevocable, royalty-free basis to BMJ Publishing Group Ltd ("BMJ") its licensees and where the relevant Journal is co-owned by BMJ to the co-owners of the Journal, to publish the Work in BMJ Open and any other BMJ products and to exploit all rights, as set out in our licence.

The Submitting Author accepts and understands that any supply made under these terms is made by BMJ to the Submitting Author unless you are acting as an employee on behalf of your employer or a postgraduate student of an affiliated institution which is paying any applicable article publishing charge ("APC") for Open Access articles. Where the Submitting Author wishes to make the Work available on an Open Access basis (and intends to pay the relevant APC), the terms of reuse of such Open Access shall be governed by a Creative Commons licence – details of these licences and which Creative Commons licence will apply to this Work are set out in our licence referred to above.

For peer review only

ABSTRACT

Introduction: National audits are used to monitor care quality and safety and are anticipated to reduce unexplained variations in quality by stimulating quality improvement. However, variation within and between providers in the extent of engagement with national audits mean that the potential for national audit data to inform quality improvement is not being realised. This study will undertake a feasibility evaluation of QualDash, a quality dashboard designed to support clinical teams and managers to explore data from two national audits, the Myocardial Ischaemia National Audit Project (MINAP) and the Paediatric Intensive Care Audit Network (PICANet).

Methods and analysis: Realist evaluation, which involves building, testing, and refining theories of how an intervention works, provides an overall framework for this feasibility study. Realist hypotheses that describe how, in what contexts, and why QualDash is expected to provide benefit will be tested across five hospitals. A controlled interrupted time series analysis, using key MINAP and PICANet measures, will provide preliminary evidence of the impact of QualDash, while ethnographic observations and interviews over 12 months will provide initial insight into contexts and mechanisms that lead to those impacts. Feasibility outcomes include the extent to which MINAP and PICANet data are used, data completeness in the audits, and the extent to which participants perceive QualDash to be useful and express the intention to continue using it after the study period.

Ethics and dissemination: The study has been approved by University of Leeds School of Healthcare Research Ethics Committee. Study results will provide an initial understanding of how, in what contexts, and why quality dashboards lead to improvements in care quality. These will be disseminated to academic audiences, study participants, hospital IT departments, and national audits. If results show a trial is feasible, we will disseminate the QualDash software through a stepped wedge cluster randomised trial.

Trial registration: ISRCTN18289782

Keywords: Dashboard, audit and feedback, quality improvement, realist evaluation

Word count: 4,218

For peer review only

ARTICLE SUMMARY

Strengths and limitations of this study

- This study will assess the feasibility of a trial of QualDash, a quality dashboard; if a trial is feasible, the findings will be used to inform the design of the definitive trial, determining the components of QualDash to be preserved, appropriate outcome measures, and the contexts in which the trial should be undertaken.
- Through a controlled interrupted time series study and qualitative multi-site case study, the study will also provide an initial understanding of whether use of a quality dashboard leads to quality improvement, how, in what contexts, and why.
- The study will contribute to understanding of how realist methods can contribute to feasibility studies and the design of trials.
- Issues of data quality may be a limitation of the controlled interrupted time series study; data completeness, and whether this changes over the course of the study, will be assessed.

INTRODUCTION

National clinical audits (NCAs), which provide comparative data on the performance of healthcare providers, are one means by which health systems around the world monitor care quality and safety. In England, a programme of over 30 NCAs is managed by the Healthcare Quality Improvement Partnership (HQIP) and all healthcare providers that contribute to delivery of the National Health Service (NHS) are required to participate. Such audits are anticipated to reduce unexplained variations in healthcare quality by stimulating quality improvement (QI) [1 2]. While there is evidence of positive impacts of NCAs [3-5], variation within and between providers in the extent to which they engage with NCAs mean the potential for NCA data to inform QI is not being realised [6 7].

Quality dashboards are a form of audit and feedback (A&F) that provide visualisations of audit data with the aim of informing QI efforts [8]. Healthcare providers are increasingly using quality dashboards. For example, quality dashboard use has been reported in Canada [9], the UK [10], and the Netherlands [11]. While quality dashboards have been shown to have positive effects on some performance indicators [9], empirical evidence regarding their impact remains limited [12].

For peer review only

QualDash

QualDash is an interactive web-based quality dashboard designed to support clinical teams and managers to explore data from two national audits, the Myocardial Ischaemia National Audit Project (MINAP) and the Paediatric Intensive Care Audit Network (PICANet), for the purpose of QI (Fig. 1). Information used to inform design of QualDash was collected through interviews with 50 clinicians and managers across five NHS Trusts (providers) and four healthcare commissioners, observations of meetings where audit data are discussed, a workshop with NCA suppliers, and two co-design workshops with clinicians and managers from one Trust.

Fig. 1: Prototype of main dashboard view for the Paediatric Intensive Care Audit Network (using simulated data).

[Figure 1 should go approximately here]

The interviews revealed that use of NCA data is largely at the clinical team level, with more limited use at divisional and corporate (Board and sub-committees that report to the Board, such as Quality and Safety Committees) levels. At all levels, a key constraint in use of NCA data for QI is lack of access to timely data; there was consensus among interviewees that data should not be more than three months old. QualDash seeks to improve access to timely data, providing users with a means to visualise the data they collect for the NCAs, without having to wait for data to be returned to them from the NCAs. There is variation between Trusts in the extent to which NCA data are used, often related to resources, which in turn impacts on timeliness of data; Trusts that make greater use of NCA data tend to have local databases from which they can generate visualisations of the data (e.g. bar charts) and audit support staff who have the time and skills to be able to generate such visualisations. In contrast, where such resources are not available, Trusts rely on the NCA annual reports, where data may be 15 months old (e.g. one annual report published in June 2017 reported data from April 2015 to March 2016). QualDash provides visualisations of key metrics, each metric being represented within a 'QualCard' (Fig. 2), enabling Trusts to use NCA data for QI, regardless of existing resources. QualCards for MINAP

For peer review only - http://bmjopen.bmj.com/site/about/guidelines.xhtml

For peer review only

and PICANet are listed in Table 1; while there is only one set of QualCards for PICANet, for MINAP an additional QualCard is provided for teaching hospitals, as discussions with sites revealed that the metrics of interest are different between teaching hospitals and District General Hospitals (DGHs). Sites are also able to create additional QualCards, to reflect local priorities.

Fig. 2: Prototype of main dashboard view for the Paediatric Intensive Care Audit Network with the Mortality QualCard expanded (using simulated data).

[Figure 2 should go approximately here]

Table 1: QualCards

	Metric
MINAP – all sites	Mortality<br>Door (arrival in Accident and Emergency) to angiogram time<br>Gold standard drugs on discharge<br>Referral for cardiac rehabilitation<br>Acute use of aspirin
MINAP – teaching hospital specific	Call (by patient/relative to emergency services) to balloon (percutaneous coronary intervention) time
PICANet – all sites	Mortality<br>48 hour unplanned readmission<br>Bed days and accidental extubation<br>Specialty case mix<br>Data quality (number of records with a missing value)<br>Patient dependency

To load new data into QualDash, NCA data are either extracted from the site's database or downloaded from the NCA website and then fed to a small script (written in R), which in turn updates the dashboard. Users can add new data as often as they want, but at a minimum they will load data into QualDash at the same time as uploading to the NCAs (typically every three months).

The benefits perceived from using QualDash may vary between sites, with under-resourced sites that previously made little use of NCA data for QI perceiving greater impact than those that already have the means to use NCA data for QI. There are also constraints on use of NCA data for QI that it may be difficult for QualDash to address. For example, in some Trusts, clinical team members perceive that relevant managers will not agree to provide the resources necessary for QI initiatives, which reduces motivation to engage with NCA data and may affect the extent to which QualDash is used. However,

For peer review only - http://bmjopen.bmj.com/site/about/guidelines.xhtml

QualDash provides means for visualisations to be downloaded and incorporated into presentations and reports, which may support clinical teams in making a stronger case for QI initiatives. Another constraint on use of NCA data for QI relates to clinicians' trust in the quality of the data. Interviews revealed variations across sites in processes for ensuring data quality. However, some interviewees also suggested that having the means to make more use of NCA data via QualDash would motivate them to improve their processes for ensuring data quality, although this will be dependent on local resources.

In this paper, we describe the methods for a realist feasibility evaluation of QualDash. The study objectives are:

1. To develop an initial understanding of how, in what contexts, and why use of QualDash leads to QI; and
2. To assess the feasibility of conducting a trial of QualDash.

As no checklists exist for reporting of realist evaluation protocols, in presenting this protocol we draw on the RAMESES II reporting standards for realist evaluations [13] (Additional file 1).

METHODS AND ANALYSIS

Study design

Use of theory is needed for design and evaluation of A&F interventions [14-16], and QI initiatives more generally [17-19]. This project draws on Realist Evaluation (RE), which involves building, testing, and refining theories about how an intervention is supposed to work [20]. These theories are expressed in the form of Context Mechanism Outcome (CMO) configurations, where C+M=O, reflecting the realist understanding that it is recipients' responses to the resources that an intervention provides (the intervention mechanisms) that determine the impact of the intervention, and such responses are highly influenced by context [21]. Consequently, RE seeks to answer not only the question of 'what works?' but 'what works for whom, in what circumstances, and why?' [22]. It is concerned with both intended and unintended outcomes. RE is recommended for studying QI [23] and has been used for studying the implementation and impact of large-scale QI programmes [24]. There is increasing interest in use of realist methods in feasibility evaluations [25-27].

For peer review only - http://bmjopen.bmj.com/site/about/guidelines.xhtml

We have drawn on a range of sources to develop CMO configurations which describe how, in what contexts, and why use of QualDash is anticipated to lead to QI (see Additional file 2). Data generated from the interviews, observations, and workshops described above have been essential to this, as have discussions with the designers of QualDash (ME and RAR) who, drawing on their expertise in information visualisation, have their own literature-informed theories regarding why certain features of QualDash will provide benefit to users [28 29]. We have also drawn on substantive theories regarding how A&F lead to QI at the micro [30 31], meso [32], and macro level [33 34].

Data collection is designed to enable testing of the CMO configurations. Outcome data, in the form of key MINAP and PICANet measures, will be collected and analysed in a controlled interrupted time series (CITS) study, while a multi-site case study [35] will provide an initial understanding of the contexts and mechanisms that lead to those outcomes, as well as providing data on intermediate outcomes such as increased use of NCA data. A&F interventions, and QI interventions more generally, require longitudinal evaluation to allow sufficient time for staff to implement changes and incorporate them into their practice [36-38]. Similarly, evaluation of health IT (HIT) should allow time for staff to integrate the technology into their practices and evolve those practices to take advantage of the functionality offered by the technology [39]. Therefore, data will be collected over a 12 month period, from August 2019.

Public and patient involvement

A Lay Advisory Group has been established, which has contributed to the design of QualDash by reviewing the topic guide for the interviews that were conducted, providing their perspective on the findings of the interview study, and participating in the usability evaluation of QualDash. For the realist feasibility evaluation, they have provided advice on aspects to pay attention to when undertaking observations. They will contribute to analysis of a sample of the qualitative data, to provide a patient perspective. They will advise on dissemination of findings to relevant interest groups and will review outputs for comprehensibility.

Setting/context

The feasibility study will be conducted in the five NHS acute Trusts in which the interview study that informed the design of QualDash was undertaken. Three Trusts are teaching hospitals that participate

For peer review only - http://bmjopen.bmj.com/site/about/guidelines.xhtml

in both MINAP and PICANet and have been selected to ensure variation in key outcome measures (MINAP: 30-day mortality for patients hospitalised with ST-elevation myocardial infarction; PICANet: risk adjusted standardised mortality ratio). Two Trusts are DGHs that participate in MINAP but do not have a PICU and so do not participate in PICANet. These have been selected to ensure variation in the same key MINAP measure.

Multi-site case study

In the multi-site case study, data will be collected through ethnographic observation and interviews. Ethnographic methods have been argued as essential for studying implementation of QI interventions [19] and introduction of HIT [40]. Ethnography is well suited to RE because it involves observing phenomena in context, supporting understanding of how context influences the response to an intervention [41]. We will follow the Biography of Artefacts approach [42], which is concerned with capturing how particular contexts and appropriations of a technology lead to different processes and generate different outcomes, a parallel to RE’s concern with contexts, mechanisms, and outcomes [43]. It involves longitudinal ‘strategic ethnography’ [42], where data collection is guided by a provisional understanding of the moments and locales in which a technology and associated practices evolve [43].

Data collection

In the three teaching hospitals, we will undertake a minimum of 24 periods of observation per Trust, to be split across activities related to cardiology and the PICU, and in the two DGHs we will undertake a minimum of 12 periods of observation per Trust, to be spent observing activities related to cardiology. Each period of observation will be a minimum of four hours (total n=384 hours). While researchers will return to each Trust monthly, to understand how use of QualDash changes over time, more time will be spent in the first few months following the introduction of QualDash, because this is when users are most likely to engage with and explore the affordances of QualDash and establish new practices around it, generating information with implications for system enhancement [43]. Observations will be scheduled to take place at different times of day and on different days of the week, to ensure the account of what is observed is as complete and representative as possible [44].

For peer review only - http://bmjopen.bmj.com/site/about/guidelines.xhtml

At each case site, an initial phase of general observation will provide an opportunity for researchers to become familiar with the setting and for those in the setting to become familiar with the presence of the researchers. Following a previous study of dashboards [10], observations will be undertaken in clinical areas to understand clinical teams' working practices and capture 'corridor committees' where issues of quality and safety are discussed more informally [45]. In the PICUs, initial observations will take place on the PICU, e.g. with the researchers positioning themselves by the nurses' station, as well as observing handovers, safety huddles, and ward rounds. Because activities related to cardiology tend to be more dispersed across hospitals, researchers will first shadow clinical team members (consultant cardiologists and acute chest pain nurses) to determine where it is most appropriate to conduct subsequent observations. These initial observations will also be used to record general details of the setting that may influence QualDash use, such as staffing levels and availability of computers.

After this initial phase, observation will be guided by the CMO configurations under investigation. In addition to observing formal meetings where quality and safety are discussed, predominantly at ward level but also at divisional and corporate level, observation will involve shadowing staff members as they undertake particular activities: collection and entry of NCA data, to see if and how this changes over time; accessing and interrogating NCA data, whether using QualDash or some other means; preparation of reports and/or presentations using NCA data, again whether using QualDash or some other means. Where visualisations from QualDash are incorporated into presentations and written reports, we will follow the path of those documents, to identify staff members who may not use QualDash directly but are receiving QualDash outputs. Attention will be paid to how, in what contexts, and why QualDash and QualDash outputs are used or not, understood in the context of broader practices and use of other sources of information for monitoring care quality, and how this changes over time. We will also follow local QI initiatives, recording data on, for example, when and how the need for the QI initiative was identified, contextual factors that appear to support and constrain its introduction, how the impact of the QI initiative is monitored, and other contextual factors that appear to influence the metric that the QI initiative is targeting. Researchers will record observations in fieldnotes, which will be written up in detail as soon after data collection as possible.

For peer review only - http://bmjopen.bmj.com/site/about/guidelines.xhtml

For peer review only

Brief interviews will be undertaken opportunistically during the course of conducting observations to clarify aspects of practice that are not immediately intelligible to an observer, with participant responses recorded in fieldnotes [46]. As data collection progresses, longer semi-structured interviews will be used to discuss revisions to our CMO configurations. These will be undertaken using a particular approach from RE, referred to as the teacher-learner cycle, whereby the theories under investigation are made explicit to the interviewee so that the interviewee can use their experiences to refine the researcher's understanding [47]. Being concerned with the reasoning of intervention recipients, mechanisms are often not observable [21], so these longer interviews will also provide the opportunity to explore staff reasoning about QualDash. These longer interviews will be audio recorded and transcribed verbatim.

Logfiles are widely used to evaluate visualisation tools [48]. QualDash logfiles will record information about the user (job title, etc.), data used (audit, year), overall time spent using QualDash, time spent interacting with different QualCards (including new QualCards that have been created), functionality used, and whether QualDash visualisations were downloaded. In addition to providing data regarding extent of QualDash use, how QualDash is used and by whom, and how this changes over time, information from logfiles will be used to inform qualitative data collection (e.g. asking in interviews why participants use particular QualCards and not others and the motivation behind the creation of new QualCards).

At the end of the data collection period, we will ask participants to complete a questionnaire based on the Technology Acceptance Model, using well validated items that have been used in numerous evaluations of HIT [49], including dashboards [50]. This will provide participants' perceptions of the usefulness of QualDash and data on whether they intend to continue using QualDash after the study period.

Analysis

An iterative approach to data collection and analysis will be taken, to enable: ongoing testing and refinement of the CMO configurations; gathering of further data in light of such revisions; and refinement of QualDash in response to participants' feedback. Fieldnotes and interview transcripts will be entered into NVivo 11. Narrative analysis will be undertaken to develop a 'biography' of QualDash, which will

For peer review only - http://bmjopen.bmj.com/site/about/guidelines.xhtml

For peer review only

describe use of QualDash and its outputs by a range of stakeholders at different levels (clinical team, divisional, and corporate) and the interconnections between them [10]. Narrative analysis is consistent with a realist approach due to its emphasis on preserving connections within the data, thereby helping to understand causality [51]. This analysis will be supplemented with analysis of the logfiles and questionnaire data. Findings will be compared with the CMO configurations, to determine whether they support, refute, or suggest a revision or addition to the CMO configurations.

Controlled interrupted time series study

Interrupted time series studies provide a robust method of assessing the effect of an intervention and have been used to assess effectiveness of a variety of complex interventions [52]. In a CITS, the addition of a control group enhances causal inference because the presence of seasonal trends and other potential time-varying confounders can be assessed [53]. Data will be collected across the five Trusts, with two control Trusts per intervention Trust, providing a total of 10 control Trusts. Control Trusts will be matched according to their size and outcomes pre-intervention. Having more than one control site per intervention site increases power but, as the number of control sites per intervention site increases, quality of matching decreases. Therefore, we have chosen to have two control Trusts per intervention Trust to increase power while maintaining quality of the matching.

Given the study intention to determine the feasibility of and inform the design of a trial, a range of measures will be considered. Initially, we selected two process measures, one for MINAP and one for PICANet. For MINAP, we selected the composite process measure Cumulative Missed Opportunities for Care (CMOC). This has nine components (pre-hospital ECG, acute use of aspirin, timely perfusion, referral for cardiac rehabilitation, and prescription at hospital discharge of what are considered to be the gold standard drugs – aspirin, thienopyridine inhibitor, ACE-inhibitor, HMG-CoA reductase inhibitor, and beta blockers) and is inversely associated with mortality [54]. As some of these components, such as pre-hospital ECG, are outside the direct control of the Trust, we will also explore the impact of QualDash on the individual measures that make up CMOC. On the basis of the measures that cardiology clinicians described in the interviews as being important for measuring care quality, we will also look at the percentage of patients who receive an angiogram within 72 hours from first admission to hospital, which is part of the Best Practice Tariff financial incentive scheme, and, for those hospitals

For peer review only - http://bmjopen.bmj.com/site/about/guidelines.xhtml

that provide percutaneous coronary intervention (PCI), the proportion of patients who have a door-to-balloon time (the time from arrival at the hospital to PCI) of less than 60 minutes. Our CMO configurations (Additional file 2) suggest improvement will be seen in measures if: clinical teams perceive them as being important indicators of care and/or they relate to financial incentives; performance is not in line with expectations; they perceive the measure as being within their control; and the team is resourced to introduce QI initiatives in relation to these measures.

For PICANet, we selected use of non-invasive ventilation first for patients requiring ventilation, which has been shown to be associated with reduced mortality [55]. However, this was not raised as an area of concern in our interviews with PICU clinicians. On the basis of this and two additional considerations – it would require loading additional data into QualDash which would reduce the performance of QualDash in terms of speed and it requires computation of the data, while the focus of QualDash is on visualising the data – a QualCard has not been created for this metric. Therefore, while we will still include this measure in the CITS, we do not hypothesise that it will change, unless other sources of information, such as the PICANet annual report, draw a PICU team's attention to it. However, accidental extubation and unplanned readmission within 48 hours were identified in our interviews with PICU clinicians as being important indicators of care quality, so we will include these two measures in the CITS. On the basis of our CMO configurations (Additional file 2), we would expect to see an improvement in these measures in sites where performance is not in line with expectations, if the team is resourced to introduce QI initiatives in relation to these measures.

Sample size considerations

For peer review only - http://bmjopen.bmj.com/site/about/guidelines.xhtml

A CITS study requires data for a minimum of three time points pre-intervention and three time points post-intervention and must also allow for any seasonal effect on the outcomes [56]. Monthly data will be obtained for 24 months pre-intervention and 12 months post-intervention. Consequently, for each intervention Trust, there will be 72 data points prior to introduction (24 for the intervention Trust and 48 for the control Trusts) and 36 data points post intervention (12 for the intervention Trust and 24 for the control Trusts). Sample size calculations were undertaken based on our two initial measures, CMOC for MINAP and use of non-invasive ventilation first for patients requiring ventilation for PICANet; full details are provided in Additional file 3.

Analysis

Monthly MINAP and PICANet data will be extracted to spreadsheets for analysis with R software [57]. For both NCAs, each outcome will be regressed upon time and the intervention. The time component will include a seasonal effect (quarterly effect) and will allow for a (linear) time trend. To account for clustering of monthly observations within hospitals, a random intercept will be fitted, although a fixed effect for hospital as a sensitivity analysis will be explored. Although the intervention is abrupt, its impact may well be 'phased in' over a few months, perhaps three. The timing of the bedding in of the intervention will be reported from the multi-site case study. Then a partial effect can be considered for this period with the interaction effect stepping up in a linear fashion.

Results of the CITS analysis will be incorporated into the biography of QualDash, the analysis of the data from the multi-site case study describing how contextual factors shape the evolution of practices around QualDash and how this leads to the resulting outcome pattern.

Trial feasibility assessment and design

Our trial progression criteria are: (i) the number of people who engage with either MINAP or PICANet data (via QualDash or some other means) is the same or higher than the number of people who engaged with either MINAP or PICANet data prior to QualDash's introduction; (ii) data completeness in the national audit improves or remains the same; (iii) 50% or more of participants in the questionnaire survey perceive QualDash to be useful and express the intention to continue using it after the study period. Criteria (i) and (ii) are concerned with ensuring the intervention does not have unintended negative consequences

For peer review only - http://bmjopen.bmj.com/site/about/guidelines.xhtml

which would affect success of the intervention. Criterion (ii) is also concerned with feasibility of outcome assessment. Criterion (iii) is concerned with acceptability and uptake of the intervention, and therefore has implications for recruitment to a trial, as well as being concerned with participants' perceptions of the impact of QualDash on care. While not formally assessed as part of the progression criteria, the impact of QualDash on care as identified in the CITS will be considered in determining whether a future trial is justified. A traffic light system will be used to determine if a trial is feasible (green), feasible with modifications to QualDash (amber), or not feasible (red) [58 59].

If the results show a trial of QualDash is feasible, we will design a stepped wedge cluster randomised trial. Data from the CITS will be used to inform the selection of NCAs to be included in the trial (MINAP and/or PICANet) and will provide information about variability of outcomes and about how long a trial intervention period would need to be. Findings from the multi-site case study will be used to inform the selection of categories of user to be included in the trial and, associated with this, the level of randomisation (Trust, hospital, or ward). Using the understanding of the relationship between contexts, mechanisms, and outcomes provided by the study, we will identify QualDash components associated with mechanisms that produce the desired outcomes in order for them to be preserved in the trial, while other components can be adapted to suit the local context.

ETHICS AND DISSEMINATION

Ethics approval has been received from the University of Leeds School of Healthcare Research Ethics Committee (Approval no.HREC16-044). Written consent will be obtained from participants for interviews and for meeting observations.

Study results will provide initial understanding of how and in what contexts quality dashboards may lead to improvements in care quality. We will disseminate these results to academic audiences, study participants, hospital IT departments, and NCAs. If we progress to a trial, in addition to providing further understanding of the impact of quality dashboards on care quality, this will result in wider dissemination of the QualDash software.

Acknowledgements

For peer review only

This research is funded by the National Institute for Health Research (NIHR) Health Services and Delivery Research (HS&DR) Programme (project number 16/04/06). The views and opinions expressed are those of the presenter and do not necessarily reflect those of the HS&DR programme, NIHR, NHS or the Department of Health.

Data statement

The qualitative data gathered during the course of this evaluation will be kept until June 2030 and can be accessed by other researchers during this time, subject to the necessary ethical approvals being obtained. Requests for access to this data should be addressed to the corresponding author.

For peer review only

Authors' contributions

RR is Principal Investigator for the study. She conceived, designed, and secured funding for the study in collaboration with JG, RW, AF, CPG, RP, JK, RAR, JL, MM, and DD. NA and LM led the qualitative data collection and analysis that informed the design of QualDash and the design of evaluation. ME developed the QualDash software and contributed to the design of the evaluation. RP and RF provided data for the testing of QualDash and provided significant feedback on its design. All authors provided input into various aspects of the evaluation design and revised drafts of the protocol. RR led the writing of this protocol manuscript. All authors read and approved the final manuscript.

Funding statement

This research is funded by the National Institute for Health Research (NIHR) Health Services and Delivery Research (HS&DR) Programme (project number 16/04/06).

Competing interests statement

Chris Gale is a member of the MINAP Academic and Steering Groups. Richard Feltbower is Principal Investigator for PICANet and Roger Parslow was previously Principal Investigator for PICANet. The authors have no other competing interests to declare.

Additional files

Additional file 1: Checklist of RAMESES II reporting standards for realist evaluations (PDF)

Additional file 2: Context Mechanism Outcome configurations to be tested in the realist feasibility evaluation (PDF)

Additional file 3: Sample size calculations for controlled interrupted time series (PDF)

REFERENCES

1. Phekoo KJ, Clements J, Bell D. National Clinical Audit Quality Assessment - Overview of the self-assessment survey: "audit of audits". London: Healthcare Quality Improvement Partnership, 2014.
2. Miller T, Leatherman S. The National Quality Forum: a'me-too'or a breakthrough in quality measurement and reporting? Health Aff. (Millwood) 1999;**18**(6):233-37.
3. Birkhead JS, Walker L, Pearson M, Weston C, Cunningham AD, Rickards AF. Improving care for patients with acute coronary syndromes: initial results from the National Audit of Myocardial Infarction Project (MINAP). Heart 2004;**90**(9):1004-09.

For peer review only - http://bmjopen.bmj.com/site/about/guidelines.xhtml

For peer review only

4. Bridgewater B, Grayson AD, Brooks N, et al. Has the publication of cardiac surgery outcome data been associated with changes in practice in northwest England: an analysis of 25 730 patients undergoing CABG surgery under 30 surgeons over eight years. Heart 2007;**93**(6):744-48.
5. Neuburger J, Currie C, Wakeman R, et al. The Impact of a National Clinician-led Audit Initiative on Care and Mortality after Hip Fracture in England: An External Evaluation using Time Trends in Non-audit Data. Med. Care 2015;**53**(8):686-91.
6. Allwood D. Engaging Clinicians in Quality Improvement through National Clinical Audit. London: Healthcare Quality Improvement Partnership, 2014.
7. Taylor A, Neuburger J, Walker K, Cromwell D, Groene O. How is feedback from national clinical audits used? Views from English National Health Service trust audit leads. J. Health Serv. Res. Policy 2016;**21**(2):91-100.
8. Pauwels K, Ambler T, Clark BH, et al. Dashboards as a Service Why, What, How, and What Research Is Needed? Journal of Service Research 2009;**12**(2):175-89.
9. Weiss D, Dunn SI, Sprague AE, et al. Effect of a population-level performance dashboard intervention on maternal-newborn outcomes: an interrupted time series study. BMJ Quality & Safety 2018;**27**(6):425-36.
10. Keen J, Nicklin E, Long A, et al. Quality and safety between ward and board: a biography of artefacts study. Health Services and Delivery Research 2018;**6**(22).
11. Weggelaar-Jansen AMJWM, Broekharst DSE, de Bruijne M. Developing a hospital-wide quality and safety dashboard: a qualitative research study. BMJ Quality & Safety 2018.
12. Dowding D, Randell R, Gardner P, et al. Dashboards for improving patient care: Review of the literature. International Journal of Medical Informatics 2015;**84**(2):87-100.
13. Wong G, Westhorp G, Manzano A, Greenhalgh J, Jagosh J, Greenhalgh T. RAMESES II reporting standards for realist evaluations. BMC Med. 2016;**14**(1):96.
14. Foy R, Eccles MP, Jamtvedt G, Young J, Grimshaw JM, Baker R. What do we know about how to do audit and feedback? Pitfalls in applying evidence from a systematic review. BMC Health Serv. Res. 2005;**5**:50.
15. Ivers NM, Grimshaw JM, Jamtvedt G, et al. Growing Literature, Stagnant Science? Systematic Review, Meta-Regression and Cumulative Analysis of Audit and Feedback Interventions in Health Care. J. Gen. Intern. Med. 2014;**29**(11):1534-41.
16. Ivers NM, Sales A, Colquhoun H, et al. No more 'business as usual' with audit and feedback interventions: towards an agenda for a reinvigorated intervention. Implementation Science 2014;**9**(1):1-8.
17. Grol RPTM, Bosch MC, Hulscher MEJL, Eccles MP, Wensing M. Planning and Studying Improvement in Patient Care: The Use of Theoretical Perspectives. Milbank Q. 2007;**85**(1):93-138.
18. Davidoff F, Dixon-Woods M, Leviton L, Michie S. Demystifying theory and its use in improvement. BMJ Quality & Safety 2015.
19. Dixon-Woods M, Bosk CL, Aveling EL, Goeschel CA, Pronovost PJ. Explaining Michigan: Developing an Ex Post Theory of a Quality Improvement Program. Milbank Q. 2011;**89**(2):167-205.
20. Pawson R, Tilley N. *Realistic Evaluation*. London: SAGE Publications, 1997.
21. Dalkin S, Greenhalgh J, Jones D, Cunningham B, Lhussier M. What's in a mechanism? Development of a key concept in realist evaluation. Implementation Science 2015;**10**(1):49.
22. Pawson R. *The science of evaluation: a realist manifesto*. London: SAGE, 2013.
23. Robert G, Fulop N. The role of context in successful improvement. Perspectives on context. A selection of essays considering the role of context in successful quality improvement. London: Health Foundation 2014;**31**.
24. Greenhalgh T, Humphrey C, Hughes J, Macfarlane F, Butler C, Pawson R. How Do You Modernize a Health Service? A Realist Evaluation of Whole-Scale Transformation in London. Milbank Q. 2009;**87**(2):391-416.

For peer review only - http://bmjopen.bmj.com/site/about/guidelines.xhtml

25. Fletcher A, Jamal F, Moore G, Evans RE, Murphy S, Bonell C. Realist complex intervention science: Applying realist principles across all phases of the Medical Research Council framework for developing and evaluating complex interventions. Evaluation 2016;**22**(3):286-303.
26. Brand SL, Quinn C, Pearson M, et al. Building programme theory to develop more adaptable and scalable complex interventions: Realist formative process evaluation prior to full trial. Evaluation 2019;**25**(2):149-70.
27. Randell R, Honey S, Hindmarsh J, et al. A realist process evaluation of robot-assisted surgery: integration into routine practice and impacts on communication, collaboration and decision-making. Health Services and Delivery Research 2017;**5**(20).
28. Andrienko N, Andrienko G. *Exploratory analysis of spatial and temporal data: a systematic approach*: Springer Science & Business Media, 2006.
29. Brehmer M, Munzner T. A multi-level typology of abstract visualization tasks. IEEE transactions on visualization and computer graphics 2013;**19**(12):2376-85.
30. Hysong SJ, Best RG, Pugh JA. Audit and feedback and clinical practice guideline adherence: Making feedback actionable. Implementation Science 2006;**1**(1):1-10.
31. Sapyta J, Riemer M, Bickman L. Feedback to clinicians: Theory, research, and practice. J. Clin. Psychol. 2005;**61**(2):145-53.
32. Van Helden GJ, Tillema S. In Search of a Benchmarking Theory for the Public Sector. Financial Accountability & Management 2005;**21**(3):337-61.
33. Scott RW. *Institutions and Organizations*. 2nd Edition ed. London: Sage, 2001.
34. Furusten S. *Institutional Theory and Organizational Change*. Cheltenham, UK: Edward Elgar, 2013.
35. Yin RK. *Case study research: design and methods*. 3rd ed. Thousand Oaks, California: SAGE, 2003.
36. Øvretveit J, Gustafson D. Evaluation of quality improvement programmes. Quality and Safety in Health Care 2002;**11**(3):270-75.
37. Ramsay AIG, Turner S, Cavell G, et al. Governing patient safety: lessons learned from a mixed methods evaluation of implementing a ward-level medication safety scorecard in two English NHS hospitals. BMJ Quality & Safety 2013;**23**:136-46.
38. Benn J, Burnett S, Parand A, Pinto A, Iskander S, Vincent C. Studying large-scale programmes to improve patient safety in whole care systems: Challenges for research. Soc. Sci. Med. 2009;**69**(12):1767-76.
39. Hyysalo S. *Health technology development and use: from practice-bound imagination to evolving impacts*. New York: Routledge, 2010.
40. Greenhalgh T, Swinglehurst D. Studying technology use as social practice: the untapped potential of ethnography. BMC Med. 2011;**9**(1):45.
41. Dainty KN, Golden BR, Hannam R, et al. A realist evaluation of value-based care delivery in home care: The influence of actors, autonomy and accountability. Soc. Sci. Med. 2018;**206**:100-09.
42. Pollock N, Williams R. *Software and organisations: The biography of the enterprise-wide system or how SAP conquered the world*. New York: Routledge, 2008.
43. Pollock N, Williams R. e-Infrastructures: How Do We Know and Understand Them? Strategic Ethnography and the Biography of Artefacts. Computer Supported Cooperative Work (CSCW) 2010;**19**(6):521-56.
44. Hammersley M, Atkinson P. *Ethnography: principles in practice*. London: Routledge, 1995.
45. Waring J. Adaptive regulation or governmentality: patient safety and the changing regulation of medicine. Sociol. Health Illn. 2007;**29**(2):163-79.
46. Goodwin D, Pope C, Mort M, Smith A. Access, boundaries and their effects: legitimate participation in anaesthesia. Sociol. Health Illn. 2005;**27**(6):855-71.
47. Pawson R. Theorizing the Interview. The British Journal of Sociology 1996;**47**(2):295-314.
48. Harrison DG, Efford ND, Fisher QJ, Ruddle RA. PETMiner—A Visual Analysis Tool for Petrophysical Properties of Core Sample Data. IEEE transactions on visualization and computer graphics 2017;**24**(5):1728-41.

For peer review only - http://bmjopen.bmj.com/site/about/guidelines.xhtml

49. Holden RJ, Karsh B-T. The Technology Acceptance Model: Its past and its future in health care. Journal of Biomedical Informatics 2010;**43**(1):159-72.
50. Lee K, Jung SY, Hwang H, et al. A novel concept for integrating and delivering health information using a comprehensive digital dashboard: An analysis of healthcare professionals' intention to adopt a new system and the trend of its real usage. Int. J. Med. Inform. 2017;**97**:98-108.
51. Maxwell JA. *A realist approach for qualitative research*. London: SAGE Publications, 2012.
52. Hu Y. Stepped wedge, natural experiments and interrupted time series analysis designs. In: Richards DA, Hallberg IR, eds. Complex interventions in health: An overview of research methods. Abingdon: Routledge, 2015:200-12.
53. Handley MA, Lyles CR, McCulloch C, Cattamanchi A. Selecting and Improving Quasi-Experimental Designs in Effectiveness and Implementation Research. Annu. Rev. Public Health 2018;**39**(1):5-25.
54. Simms AD, Baxter PD, Cattle BA, et al. An assessment of composite measures of hospital performance and associated mortality for patients with acute myocardial infarction. Analysis of individual hospital performance and outcome for the National Institute for Cardiovascular Outcomes Research (NICOR). European Heart Journal: Acute Cardiovascular Care 2013;**2**(1):9-18.
55. Morris JV, Ramnarayan P, Parslow RC, Fleming SJ. Outcomes for Children Receiving Noninvasive Ventilation as the First-Line Mode of Mechanical Ventilation at Intensive Care Admission: A Propensity Score-Matched Cohort Study. Crit. Care Med. 2017.
56. Cochrane Effective Practice and Organisation of Care Group (EPOC). What study designs should be inlcuded in an EPOC review and what should they be called? Oslo: Norwegian Knowledge Centre for the Health Services, 2013.
57. R Core Team. R: A language and environment for statistical computing. Vienna, Austria: R Foundation for Statistical Computing, 2014.
58. Bugge C, Williams B, Hagen S, et al. A process for Decision-making after Pilot and feasibility Trials (ADePT): development following a feasibility study of a complex intervention for pelvic organ prolapse. Trials 2013;**14**(1):353.
59. Thabane L, Ma J, Chu R, et al. A tutorial on pilot studies: the what, why and how. BMC Med. Res. Methodol. 2010;**10**(1):1-10.

For peer review only

For peer review only - http://bmjopen.bmj.com/site/about/guidelines.xhtml

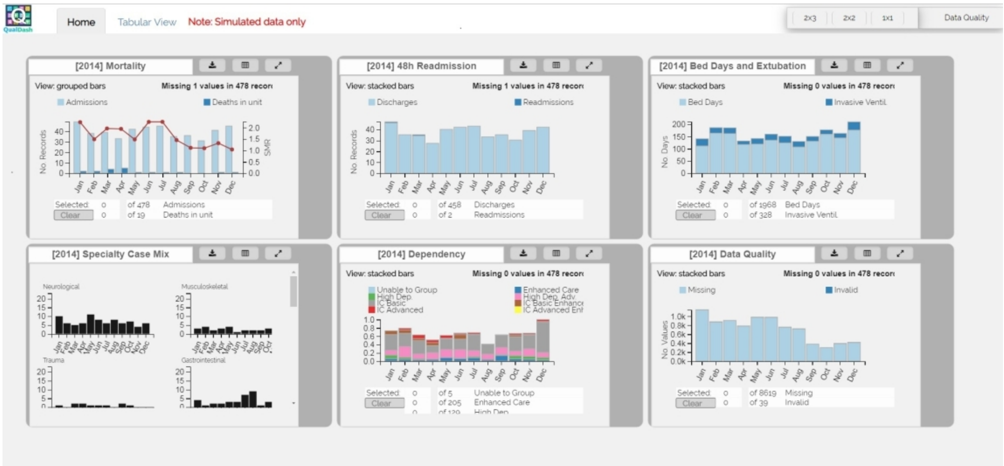

Fig. 1: Prototype of main dashboard view for the Paediatric Intensive Care Audit Network (using simulated data).

161x74mm (300 x 300 DPI)

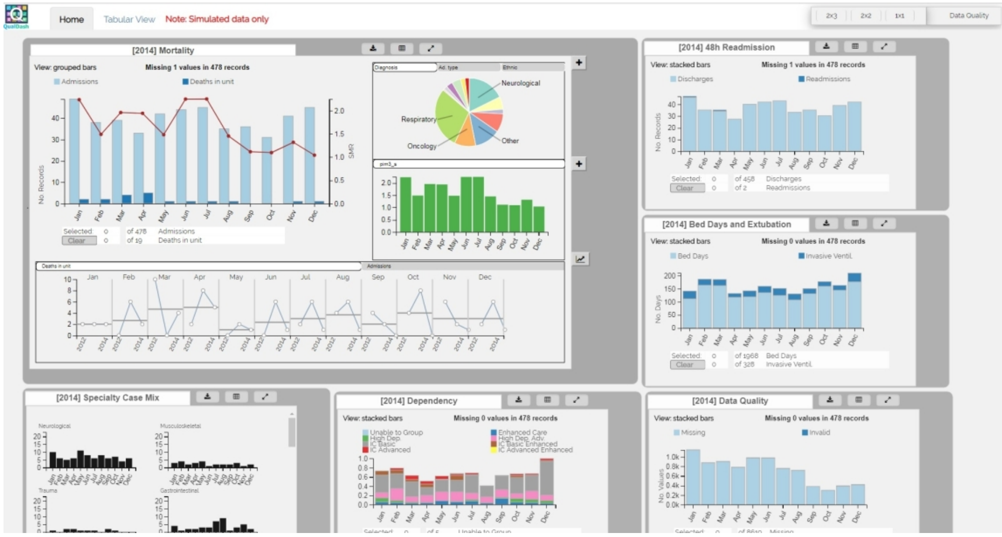

Fig. 2: Prototype of main dashboard view for the Paediatric Intensive Care Audit Network with the Mortality QualCard expanded (using simulated data).

161x85mm (300 x 300 DPI)

RAMESES II reporting standards for realist evaluations

**How, in what contexts, and why do quality dashboards lead to improvements in care quality in acute hospitals? Protocol for a realist feasibility evaluation**			**Reported in document Y/N/NA**	**Page(s) in document**	**Comment**
1		In the title, identify the document as a realist evaluation	Y	1	
SUMMARY OR ABSTRACT					
2		Journal articles will usually require an abstract, while reports and other forms of publication will usually benefit from a short summary. The abstract or summary should include brief details on: the policy, programme or initiative under evaluation; programme setting; purpose of the evaluation; evaluation question(s) and/or objective(s); evaluation strategy; data collection, documentation and analysis methods; key findings and conclusions<br><br>Where journals require it and the nature of the study is appropriate, brief details of respondents to the	Y	3	

For peer review only

		evaluation and recruitment and sampling processes may also be included<br><br>Sufficient detail should be provided to identify that a realist approach was used and that realist programme theory was developed and/or refined			
INTRODUCTION					
3	Rationale for evaluation	Explain the purpose of the evaluation and the implications for its focus and design	Y	7	
4	Programme theory	Describe the initial programme theory (or theories) that underpin the programme, policy or initiative	Y	8 and Additional file 2	Placed in body of article, rather than Introduction, as more appropriate for protocol
5	Evaluation questions, objectives and focus	State the evaluation question(s) and specify the objectives for the evaluation. Describe whether and how the programme theory was used to define the scope and focus of the evaluation	Y	7	
6	Ethical approval	State whether the realist evaluation required and has gained ethical approval from the relevant	Y	15	

		authorities, providing details as appropriate. If ethical approval was deemed unnecessary, explain why			
METHODS					
7	Rationale for using realist evaluation	Explain why a realist evaluation approach was chosen and (if relevant) adapted	Y	7-8	
8	Environment surrounding the evaluation	Describe the environment in which the evaluation took place	Y	8-9	
9	Describe the programme policy, initiative or product evaluated	Provide relevant details on the programme, policy or initiative evaluated	Y	5-7	Description of intervention placed in Introduction as this seemed more appropriate in providing the context for the protocol
10	Describe and justify the evaluation design	A description and justification of the evaluation design (i.e. the account of what was planned, done and why) should be included, at least in summary form or as an appendix, in the document which presents the main findings. If this is not done, the omission should be justified and a reference or link to the evaluation	Y	7-14	

For peer review only

		design given. It may also be useful to publish or make freely available (e.g. online on a website) any original evaluation design document or protocol, where they exist			
11	Data collection methods	Describe and justify the data collection methods – which ones were used, why and how they fed into developing, supporting, refuting or refining programme theory<br><br>Provide details of the steps taken to enhance the trustworthiness of data collection and documentation	Y	9-11	
12	Recruitment process and sampling strategy	Describe how respondents to the evaluation were recruited or engaged and how the sample contributed to the development, support, refutation or refinement of programme theory	Y	8-9	Sampling of sites, rather than individuals, is described; Recruitment will be described when reporting the results of the study
13	Data analysis	Describe in detail how data were analysed. This section should include information on the constructs that were identified, the process of	Y	11-12, 13-14	

For peer review only

		analysis, how the programme theory was further developed, supported, refuted and refined, and (where relevant) how analysis changed as the evaluation unfolded			
RESULTS					
14	Details of participants	Report (if applicable) who took part in the evaluation, the details of the data they provided and how the data was used to develop, support, refute or refine programme theory	NA		Protocol so no results to report
15	Main findings	Present the key findings, linking them to contexts, mechanisms and outcome configurations. Show how they were used to further develop, test or refine the programme theory	NA		Protocol so no results to report
DISCUSSION					
16	Summary of findings	Summarise the main findings with attention to the evaluation questions, purpose of the evaluation, programme theory and intended audience	NA		Protocol so no results to report
17	Strengths, limitations and future directions	Discuss both the strengths of the evaluation and its limitations. These should include (but need not be	4		Strengths and limitations of study design

For peer review only

		limited to): (1) consideration of all the steps in the evaluation processes; and (2) comment on the adequacy, trustworthiness and value of the explanatory insights which emerged<br><br>In many evaluations, there will be an expectation to provide guidance on future directions for the programme, policy or initiative, its implementation and/or design. The particular implications arising from the realist nature of the findings should be reflected in these discussions			
18	Comparison with existing literature	Where appropriate, compare and contrast the evaluation's findings with the existing literature on similar programmes, policies or initiatives	NA		Protocol so no results to compare with existing literature
19	Conclusion and recommendations	List the main conclusions that are justified by the analyses of the data. If appropriate, offer recommendations consistent with a realist approach	NA		Protocol so no results on which to base recommendations
20	Funding and conflict of interest	State the funding source (if any) for the evaluation, the role played by the	Y	16	

For peer review only

		funder (if any) and any conflicts of interests of the evaluators			

For peer review only

How, in what contexts, and why do quality dashboards lead to improvements in care quality in acute hospitals? Protocol for a realist feasibility evaluation (Randell et al.)

Additional file 2

#	Context	+	Mechanism: Resource	Mechanism: Response	=	Outcome
**1.**	Teams previously constrained in their ability to use NCA data for monitoring service performance because data not considered to be timely, accurate, and/or complete	+	QualDash offers easy access to key metrics	Teams are able to see whether the data displayed are timely, accurate, and/or complete and, where they are not, adjust their data collection processes in order to benefit from QualDash	=	Improvement in data quality in terms of timeliness, accuracy, and completeness – as data quality improves, use of QualDash increases
				Teams use QualDash to embed NCA data within their monitoring processes e.g. in clinical governance meetings where data is presented visually via screens.	=	Increased routine use of NCA data in performance monitoring, providing opportunities for its use in quality improvement
**2.**	Teams previously using NCA data to monitor service performance routinely by extracting raw data and producing reports for review in meetings and by individuals	+	QualDash visualises key metrics in ways that clearly show whether service performance is within an expected range and provides functions to interrogate that data	Teams use QualDash to facilitate their existing processes for monitoring service performance using NCA data	=	Reduced time spent in accessing, and preparing visualisations of, NCA data

For peer review only - http://bmjopen.bmj.com/site/about/guidelines.xhtml

#	Context	+	Mechanism		=	Outcome
			Resource	Response		
**3.**	Teams who want to use NCA data but were previously constrained by data quality and existing systems did not provide functions to easily access and interact with the data	**+**	QualDash provides functions that enable users to interact with NCA data and explore relationships between variables	Teams will use these functions to interrogate anomalies in the data, which will help them to understand what has impacted performance, thereby enabling them to identify appropriate strategies for improving performance	**=**	Introduction of QI initiatives in relation to metrics that teams consider important and where performance is not in line with expectations<br><br>Over time, improvement in metrics that QI initiatives target
**4.**	Performance in key metrics, such as the Best Practice Tariff, is in line with expectations<br><br>Relevant audit/IT support staff have time and willingness to support use of QualDash	**+**	QualDash offers teams the ability to quickly and easily add new QualCards (within NCA parameters)	Teams add new QualCards to be able to monitor and interrogate metrics they have chosen as important	**=**	Introduction of QI initiatives in relation to metrics shown on new QualCards when performance is not in line with expectations<br><br>Over time, improvement in metrics that QI initiatives target

For peer review only - http://bmjopen.bmj.com/site/about/guidelines.xhtml

#	Context	+	Mechanism: Resource	Mechanism: Response	=	Outcome
**5.**	Teams who previously did not, or were not able to, monitor key metrics routinely<br><br>Performance is not in line with expectations in key metrics<br><br>Teams are resourced to make practice changes	**+**	QualDash provides quick and easy access to key metrics	Teams will become aware of discrepancies between performance and targets in key metrics, which they will take action to address	**=**	Introduction of QI initiatives in relation to key metrics<br><br>Over time, improvement in those metrics
**6.**	Teams are asked to produce reports and recommendations for managers and other groups about service performance, e.g. at the time of publication of NCA annual report	**+**	QualDash offers easy access to NCA data and visualisations that can be exported into reports	Teams will use QualDash to produce performance reports requested by other groups	**=**	Reduced time spent in report preparation<br><br>Increased use of NCA data at divisional and corporate levels via outputs produced by QualDash<br><br>Over time, use of QualDash at divisional and/or corporate levels, due to increased awareness of NCA data

For peer review only

#	Context	+	Mechanism		=	Outcome
			**Resource**	**Response**		
**7.**	Teams receive data requests from service managers	**+**	QualDash can be easily accessed via the web by multiple users	Service managers will use QualDash to access the information they need quickly and easily	**=**	Streamlines the use of NCA data for clinical managers<br><br>Reduced time spent by audit support staff/clinical team in producing data reports for managers
**8.**	Teams need to evidence their performance to managers and other groups in order to support a case for practice change e.g. in business meetings with managers or in the NCA annual report summary	**+**	QualDash visualises performance metrics, which can also be exported into reports and presentations	Teams will use these functions to evidence service performance, in order to convince other Trust groups that change is needed	**=**	Other Trust groups, who are able to offer additional resource to teams, are convinced of the need for change based on the evidence provided. However, this is likely to be where those outputs are clearly associated with Trust priorities, e.g. relating to Trust reputation or avoiding penalties/gaining incentives.

For peer review only

How, in what contexts, and why do quality dashboards lead to improvements in care quality in acute hospitals? Protocol for a realist feasibility evaluation (Randell et al.)
Additional file 3

The average CMOC for patients on a ward will be averaged for each month, so that there are 36 observations clustered within each of 15 hospitals. Taking the intra-class correlation to be 0.15, this yields a design effect of 6.25. Hence the effective number of observations is 15*36/6.25=86.4. Using Cohen's approach to sample size calculation means an effect size of 0.17 can be estimated with 80% given that there are six parameters in the model (including the coefficient for QualDash). Converting this to the percentage of variation that can be explained by the model, this yields 20.5%. Translating this back to CMOC, currently 49.6% of patients are discharged from hospital without missing any of the nine opportunities for care, and we would be powered at the 80% level to detect an improvement from an average of 8.33 opportunities achieved to 8.46. Thus our study has good power to detect small but meaningful clinical improvements. For PICANet, 10% of the admitted population receive non-invasive ventilation first [1]. On average there are 5.25 ventilation cases per month per hospital. With a further design effect from patients clustered within hospitals, based on the reported intraclass correlation coefficient of 0.065 giving a design effect of 1.276, the actual anticipated number of patients is 1701 giving an effective number of 213: 71 exposed to QualDash and 142 controls. This yields 80% power to detect a change from 32% to 53%.

References

1. Morris JV, Ramnarayan P, Parslow RC, Fleming SJ. Outcomes for Children Receiving Noninvasive Ventilation as the First-Line Mode of Mechanical Ventilation at Intensive Care Admission: A Propensity Score-Matched Cohort Study. Crit. Care Med. 2017.

For peer review only
